# Complexation of molecular clips containing fragments of diphenylglycoluril and benzocrown ethers with paraquat and its derivatives

**DOI:** 10.3762/bjoc.13.203

**Published:** 2017-10-04

**Authors:** Leonid S Kikot', Catherine Yu Kulygina, Alexander Yu Lyapunov, Svetlana V Shishkina, Roman I Zubatyuk, Tatiana Yu Bogaschenko, Tatiana I Kirichenko

**Affiliations:** 1 Department of Fine Organic Synthesis, A.V. Bogatsky Physico-Chemical Institute, National Academy of Sciences of Ukraine, Lustdorfskaya doroga 86, Odesa 65080, Ukraine; 2 Department of X-ray Diffraction Studies and Quantum Chemistry, SSI ‘‘Institute for Single Crystals’’, National Academy of Sciences of Ukraine, Nauky Ave. 60, Kharkiv 61001, Ukraine; 3 Department of Inorganic Chemistry, V.N. Karazin Kharkiv National University, 4 Svobody Sq., 61122, Kharkiv, Ukraine

**Keywords:** complexation, crown ethers, "host–guest chemistry", molecular clips, paraquat

## Abstract

The complexation of molecular clips containing fragments of diphenylglycoluril and benzocrown ethers with paraquat and its derivatives has been studied both in solution and in the solid state. In this paper we studied the influence of the crown ether ring size and the nature of the substituents at the nitrogen atoms of the paraquat derivatives on the composition and stability of these complexes.

## Introduction

After the first report on the synthesis of crown ethers and their complexation properties made by Pedersen in 1967, "host–guest chemistry" attracted great attention [1]. In subsequent years, various types of crown compounds have been obtained, their complexation with metal ions, ammonium, and alkylammonium salts has been extensively studied. After Stoddart and co-workers in 1987 [2] reported the complexation of crown ethers with paraquat and diquat for the first time, these compounds have become the most commonly used models in the design of various systems such as host–guest and supramolecular assemblies based on crown ethers [3]. The development of these studies led to the preparation of rotaxanes and catenanes, and to the elaboration of the principles of molecular machines, for which J. Fraser Stoddart [4–6], Jean-Pierre Sauvage [7–8] and Bernard L. Feringa [9–10] received the Nobel Prize in chemistry in 2016. In addition to Stoddart, the groups of Gibson [11], Huang [12], Chiu [13], Balzani [14] and Loeb [15] have achieved great success in this field. However, flexible structures and the relatively similar nature of crown ether complexation may limit, to a certain extent, their further use. Therefore, the search for new receptors based on crown ethers differing in the structure of the intramolecular cavity and in the type of complexation, is still relevant. Among the large variety of synthetic receptors, so-called molecular tweezers and clips are of interest [16–17]. These are a particular case of U-shaped hosts, highly reorganized receptors with rigid cleft and convergent binding sites. These compounds are disposed to selective complexation with a wide range of guests. The formation of complexes in this case occurs due to fixation of the substrate molecule between the sidewalls of the molecular clips containing donor centers, similar to the operating principle of mechanical clips. The most promising for the interaction with paraquat derivatives are molecular tweezers and clips containing fragments of crown ethers, although such examples are not numerous. Nolte et al. showed that the introduction of a glycoluril moiety into bis(paraphenylene)-34-crown-10 leads to changes in the paraquat complex structure and an increase in its stability from 730 M^−1^ to 20000 M^−1^ in acetone-*d*_6_ [18]. Chen and co-workers obtained receptors with different combinations of triptycene, pentiptycene and crown ether fragments, and their complexation with a variety of guests has been studied [19–25]. Recently, they have obtained new molecular tweezers containing a tetraphenylene group as rigid fragment and two dibenzo-24-crown-8 fragments as side arms, and their complexation with paraquat derivatives have been investigated [26].

**Figure 1:**
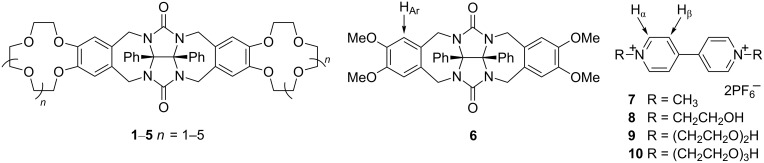
Chemical structures of hosts **1**–**6** and guests **7**–**10**.

Previously, we obtained molecular clips containing diphenylglycoluril and benzocrown ether fragments **1**–**5**, and studied their complexation with alkali metal [27–28]. In this report, we discuss the interaction of given molecular clips and the model clip based on veratrole **6** with paraquat (**7**) and its analogues **8**–**10** (Figure 1).

## Results and Discussion

### Complexation studies

**Figure 2:**
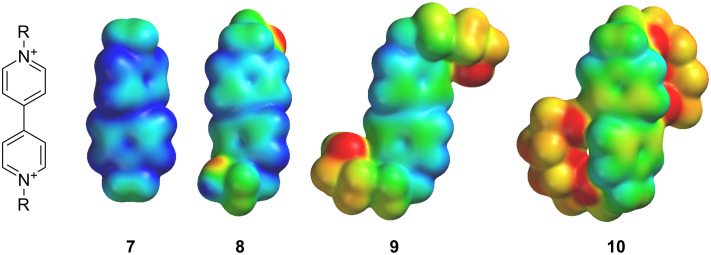
HF/6-311+G** calculated 3D molecular electrostatic potential of the guests **7**–**10**. The color code spans from 138 (red) to 177 kcal/mol (blue).

Paraquat (**7**) and its derivatives are among the most studied electron-acceptor guests that can form stable inclusion complexes with electron-donating molecules. Stabilization of such inclusion complexes is mainly realized due to π–π stacking and C–H∙∙∙X (X = N, O, F ...) interactions. The stability of inclusion complexes based on molecular clips and paraquat derivatives depends on two factors: 1) the value of the positive charge on the dipyridinium fragment of paraquat derivatives, the distribution of which is well described by the molecular electrostatic potential (MEP, Figure 2); 2) the number of oxygen atoms of the polyether chain involved in complex stabilization due to intermolecular C–H···О bonds.

For qualitative estimation of the complexing properties of the obtained molecular clips **1**–**6**, we used the approach approved earlier and described in our previous studies [29–30].

FAB–MS is known as a versatile method to detect supramolecular complexes from solutions after transferring them into the gas phase [31–32].

The solutions containing equimolar amounts of clips **1**–**5** and paraquat (**7**) in 3-nitrobenzyl alcohol were subjected to mass spectral analysis. Ion peaks resulting from the loss of the hexafluorophosphate anion from 1:1 complexes of the appropriate molecular clip with paraquat (**7**) have been observed in the mass spectra (Figures S22–S44, Supporting Information File 1). A similar spectral pattern is also observed for other complexes of the clips **1**–**5** with paraquat derivatives **8**–**10**. This is typical for most pseudorotaxanes and catenanes and an evidence of the fairly stable host–guest inclusion complexes [33–34].

**Figure 3:**
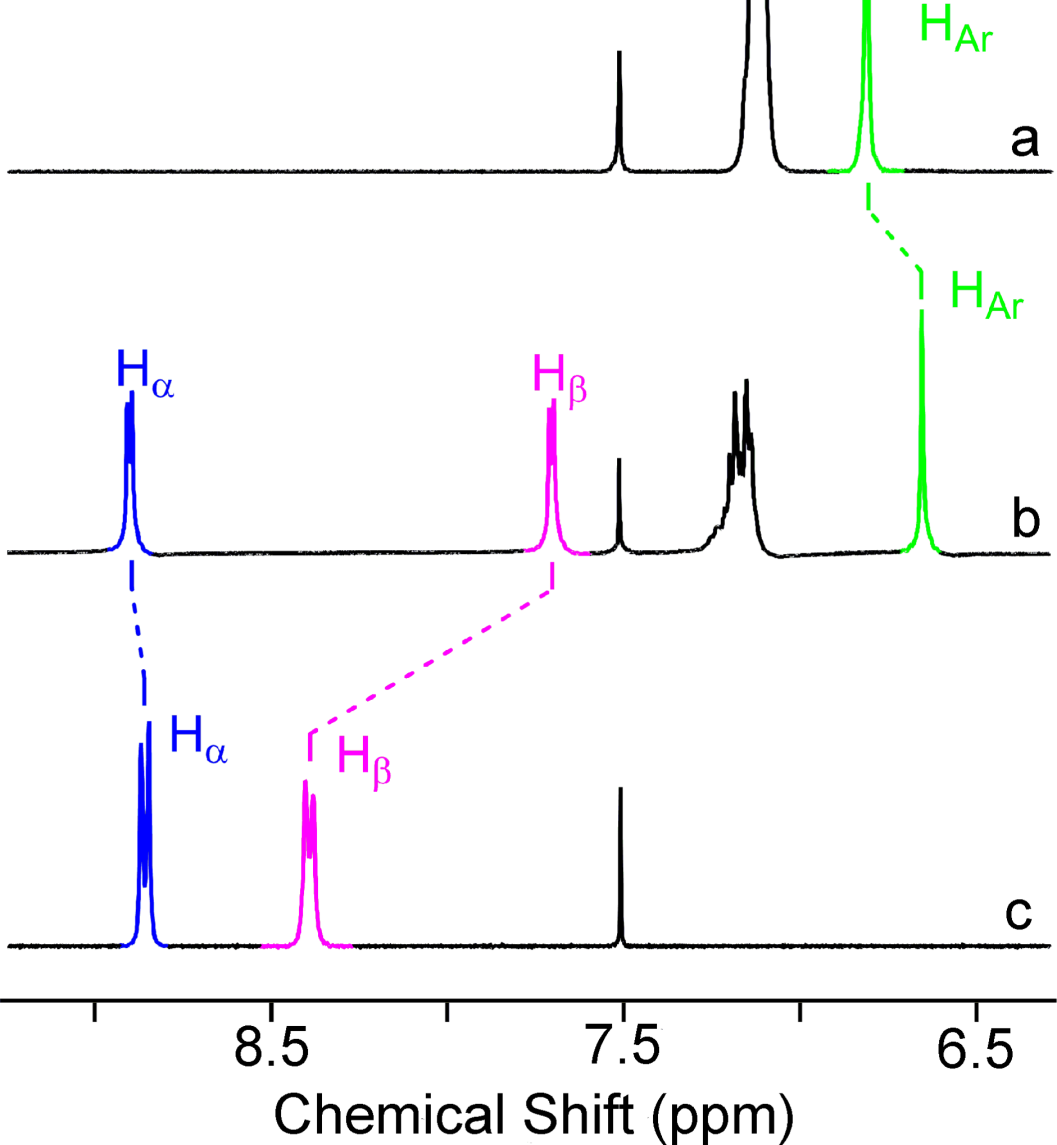
Partial ^1^H NMR spectra (300 MHz, CD_3_CN/CDCl_3_ 4:3, v/v) of (a) free host **3**, (b) **3** and 1.0 equiv of **7**, and (c) free guest **7**.

The ability of the obtained clips **1**–**5** to act as receptors for the guests **7**–**10** in solution was also investigated by ^1^H NMR spectroscopy. In the ^1^H NMR spectra of equimolar mixtures of the clips **1**–**5** with paraquat (**7**), significant upfield shifts of Н_β_ aromatic proton signals of paraquat (**7**, compared to their position in the spectra of the individual compound) have been observed as a result of their shielding by aromatic fragments of clip (Figure 3, Table 1).

**Table 1:** Induced chemical shifts (Δδ) of aromatic protons of guests **7**–**10** (H_α_ and H_β_) in the ^1^H NMR spectra of their equimolar mixtures with clips **1**–**6**.

	Clip							

Guest	**7**		**8**		**9**		**10**	

	H_α_	H_β_	H_α_	H_β_	H_α_	H_β_	H_α_	H_β_

**6**	−0.04	−0.06	−0.04	−0.09	−0.02	−0.01	n.d.^a^	n.d.
**1**	−0.16	−0.28	−0.10	−0.29	−0.07	−0.41	−0.10	−0.28
**2**	−0.16	−0.37	−0.12	−0.31	−0.05	−0.52	−0.10	−0.36
**3**	0.05	−0.70	−0.06	−0.49	0.07	−0.71	−0.01	−0.59
**4**	0.05	−0.63	−0.02	−0.52	0.08	−0.66	n.d.	n.d.
**5**	0.08	−0.37	−0.05	−0.49	0.07	−0.68	n.d.	n.d.

^a^n.d. = not determined.

Signals of the Н_α_ protons of the guests **7**–**10** are shifted insignificantly. There are upfield shifts for clips **1**, **2** and **6** and downfield shifts for clips **3**–**5** (Table 1) [19–20,26]. Protons of the C_6_H_2_ groups of the clips are shifted upfield as a result of their shielding by the aromatic fragments of paraquat. In addition, changes in the signal positions of some protons in the polyether chain (CH_2_O) as well as signals of methyl and methylene protons of the guests have been revealed (Figures S1–S21, Supporting Information File 1).

**Figure 4:**
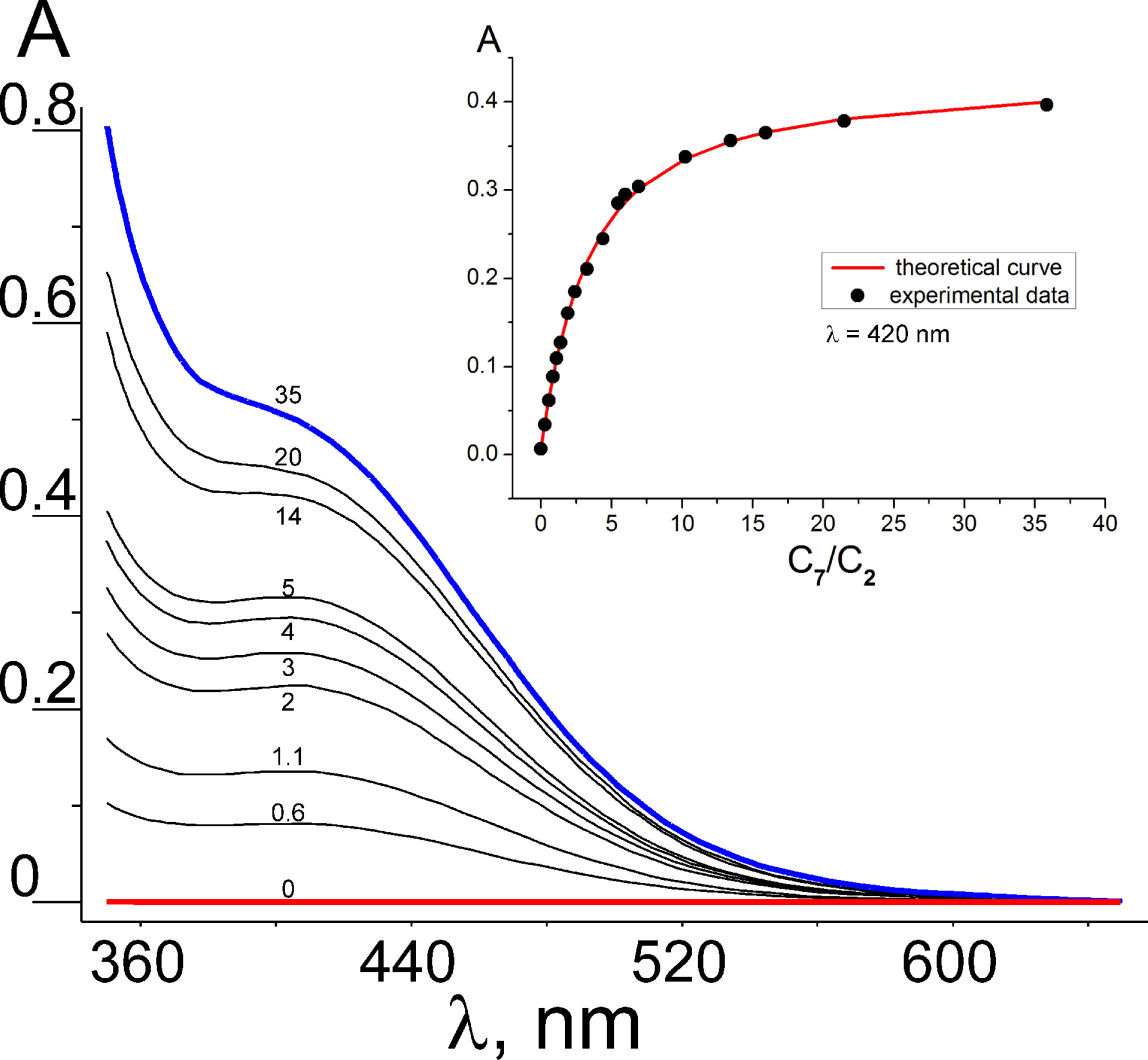
The changes observed in the UV–vis spectra during the titration of clip **2** with paraquat (**7**) in acetonitrile at 20 °C. The labels near each plot correspond to the equivalents of paraquat (**7**) added.

The analysis of UV–vis spectra could also provide qualitative information on the formation of inclusion complexes in solution. The addition of guests **7**–**10** to a solution of clips **1**–**5** turns the colourless solution to yellow, and a new wide absorption band (370–560 nm) appears. The new band was assigned to a charge-transfer complex formed by the π-donor aromatic fragments of clips **1**–**5** and the π-acceptor dipyridinium fragment of paraquat derivatives **7**–**10** located in the pseudo cavity of the clip [20]. The intensity of this band increases with the raise of the molar ratio of paraquat:clip. The observed spectral changes in the visible region of the spectrum were used to determine the stability constants of formed complexes by spectrophotometric titration. The titration was carried out in acetonitrile at 20 °C by the method of molar ratios while maintaining the levels of molecular clips **1**–**5** constant and systematically varying the concentration of paraquat derivatives **7**–**10**. The obtained experimental data were processed by the non-linear method of least squares using the SIRKO program [35]. The values of the complexes extinctions and their stability constants were herewith adjustable parameters (Figures S46–S64, Supporting Information File 1). Typical changes in the UV–vis spectra during the titration of clip **2** with paraquat (**7**) are presented in Figure 4.

**Figure 5:**
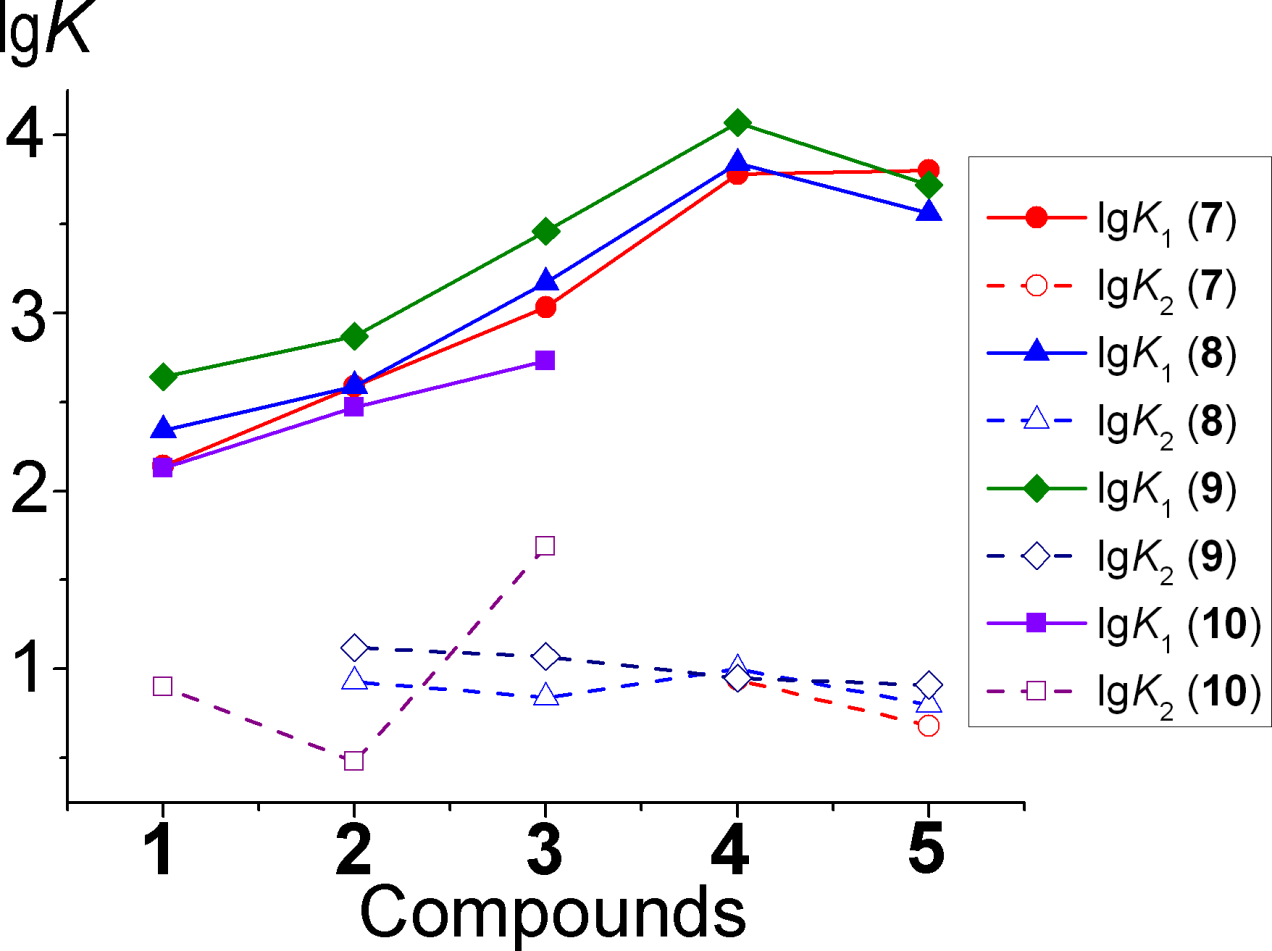
Stability constant dependence for the complexes (lg*K*) of molecular clips **1**–**5** with guests **7**–**10** on the size of crown ether cycles in acetonitrile at 20 °C.

The data obtained for molecular clips **1**–**3** are best described by titration curves corresponding to 1:1, and for clips **4** and **5** – to 1:1 and 1:2 complexes. Stability constants of the complexes are presented in Figure 5 and Table 2.

**Table 2:** Complex stability constants of molecular clips **1**–**5** with guests **7**–**10** in acetonitrile at 20 °C.

	**7**		**8**		**9**		**10**	

	lg*K*_1_	lg*K*_2_	lg*K*_1_	lg*K*_2_	lg*K*_1_	lg*K*_2_	lg*K*_1_	lg*K*_2_

**1**	2.14 ± 0.01	–	2.34 ± 0.01	–	2.64 ± 0.01	–	2.13 ± 0.01	0.90 ± 0.01
**2**	2.59 ± 0.01	–	2.59 ± 0.01	0.93 ± 0.01	2.87 ± 0.01	1.12 ± 0.01	2.47 ± 0.01	0.48 ± 0.01
**3**	3.03 ± 0.01	–	3.17 ± 0.01	0.84 ± 0.01	3.46 ± 0.01	1.07 ± 0.01	2.73 ± 0.01	1.69 ± 0.02
**4**	3.78 ± 0.03	0.94 ± 0.06	3.84 ± 0.04	1.00 ± 0.04	4.07 ± 0.05	0.95 ± 0.04	n.d.^a^	n.d.
**5**	3.80 ± 0.03	0.68 ± 0.05	3.56 ± 0.02	0.80 ± 0.04	3.72 ± 0.02	0.91 ± 0.02	n.d.	n.d.

^a^n.d. = not determined.

On the average, the stability of clip complexes with paraquat (**7**) is increased by ≈0.6 kcal/mol with increasing of the polyether macrocycle size by one oxyethylene fragment reaching a maximum for the clip **4** (Table 3). In the interaction with paraquat, the following moieties may be involved: the glycoluril fragment (hydrogen bonds involving the oxygen atoms of the carbonyl groups), the catechol part of the crown ether fragments (π–π stacking interactions) and the polyether chains of benzocrown ethers (C–H···О interactions). The first two subunits are identical for all molecular clips. The number of polyether links varies with alteration of the crown ether ring size. To assess the contributions of the glycoluril fragment and the aromatic side walls to the complex stabilities of molecular clips with paraquat we have determined the complexation constant of the clip **6** with paraquat (**7**), which was: lg*K* = 1.46 ± 0.01 (−ΔG = 1.96 ± 0.02 kcal/mol). The complex of clip **6** with paraquat (**7**) may be stabilized through π–π stacking interactions of the electron-deficient aromatic rings of paraquat and the electron donating veratrol fragments of the clips, as well as through hydrogen bonds between the H_α_, H_β_, and CH_3_ protons of paraquat with oxygen atoms of carbonyl groups in glycoluril fragment.

The contribution of the polyether chains to the stabilization of the complex with 1:1 composition, in the first approximation, may be considered as the difference between the complexation energy of the clips with the crown ether moieties **1**–**5** and the complexation energy of clip **6** with paraquat (**7**, Table 3).

**Table 3:** Contribution of polyether oxygen atoms of molecular clips **1**–**5** to the binding of paraquat.

	**6**	**1**	**2**	**3**	**4**	**5**

−Δ*G*^a^, kcal/mol	1.96	2.87	3.47	4.06	5.07	5.09
−ΔΔ*G*^b^, kcal/mol	–	0.91	1.51	2.10	3.11	3.13

^a^Δ*G* = free energy of complexation corresponding to a 1:1 complex. ^b^ΔΔ*G* = Δ*G***_1_**_-_**_6_** − Δ*G***_6_**.

Therefore, the glycoluril fragment makes the predominant contribution to the complex stability of clips **1** and **2** with paraquat (**7**), whereas the polyether chain – to that of the complexes of the clips **3**–**5** with paraquat (**7**). The data obtained show that the clips **4** and **5** are maximum pre-organized to form a 1:1 complex with paraquat compared to other clips with benzocrown ether moieties.

### X-ray crystallography

**Figure 6:**
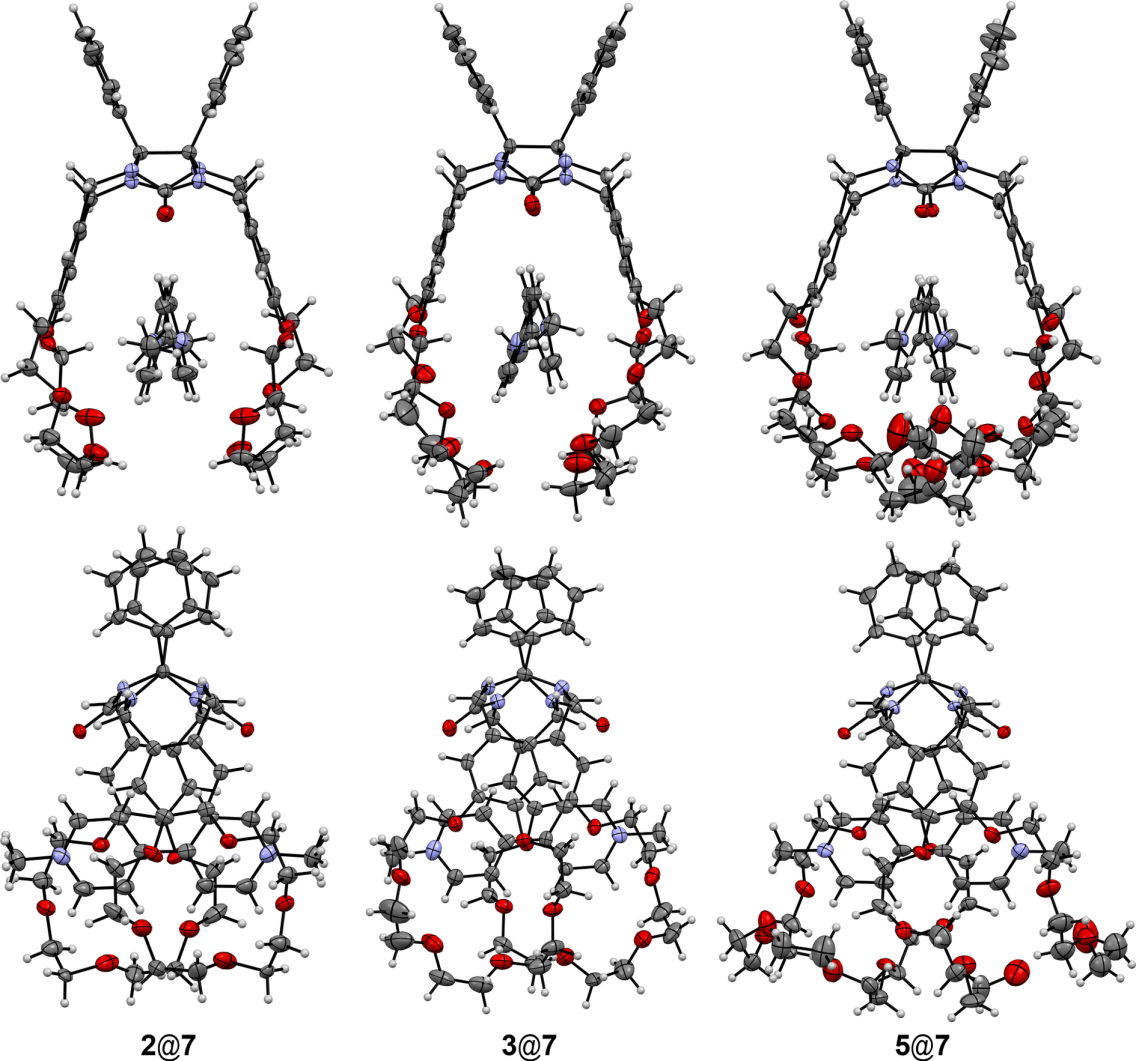
Molecular structures of complexes of clips **3**, **2** and **5** with paraquat (**7**). Anions and solvate molecules are omitted for clarity.

The structures of the complexes of clips **2**, **3** and **5** with paraquat (**7**) have been studied by X-ray crystallography. All these clips form inclusion complexes with a 1:1 ratio where the paraquat molecule is located within the pseudo-cavity formed by two crown ether fragments (Figure 6).

The complexes are stabilized by π–π intermolecular stacking interactions between the electron-donating aromatic fragments of the clips and the electron-deficient dipyridinium fragments of paraquat. The distances between the centroids of these fragments are 4.07 Å for **2**, 3.91, 4.08 Å for **3** and 4.02, 4.06 Å for **5** that is typical for such type of interactions. The complexes are also stabilized by the C–H···O interactions between the aromatic dipyridinium protons and the oxygen atoms of the polyether macrocycle or the oxygen atoms of the carbonyl groups (the H···O distances vary in the range of 2.33–2.89 Å). Additionally the host–guest structures are stabilized by weak C–H···π interactions of the hydrogen atoms of the methylene groups and dipyridinium fragments of paraquat (the corresponding H···π distances are ≈2.84 Å).

**Figure 7:**
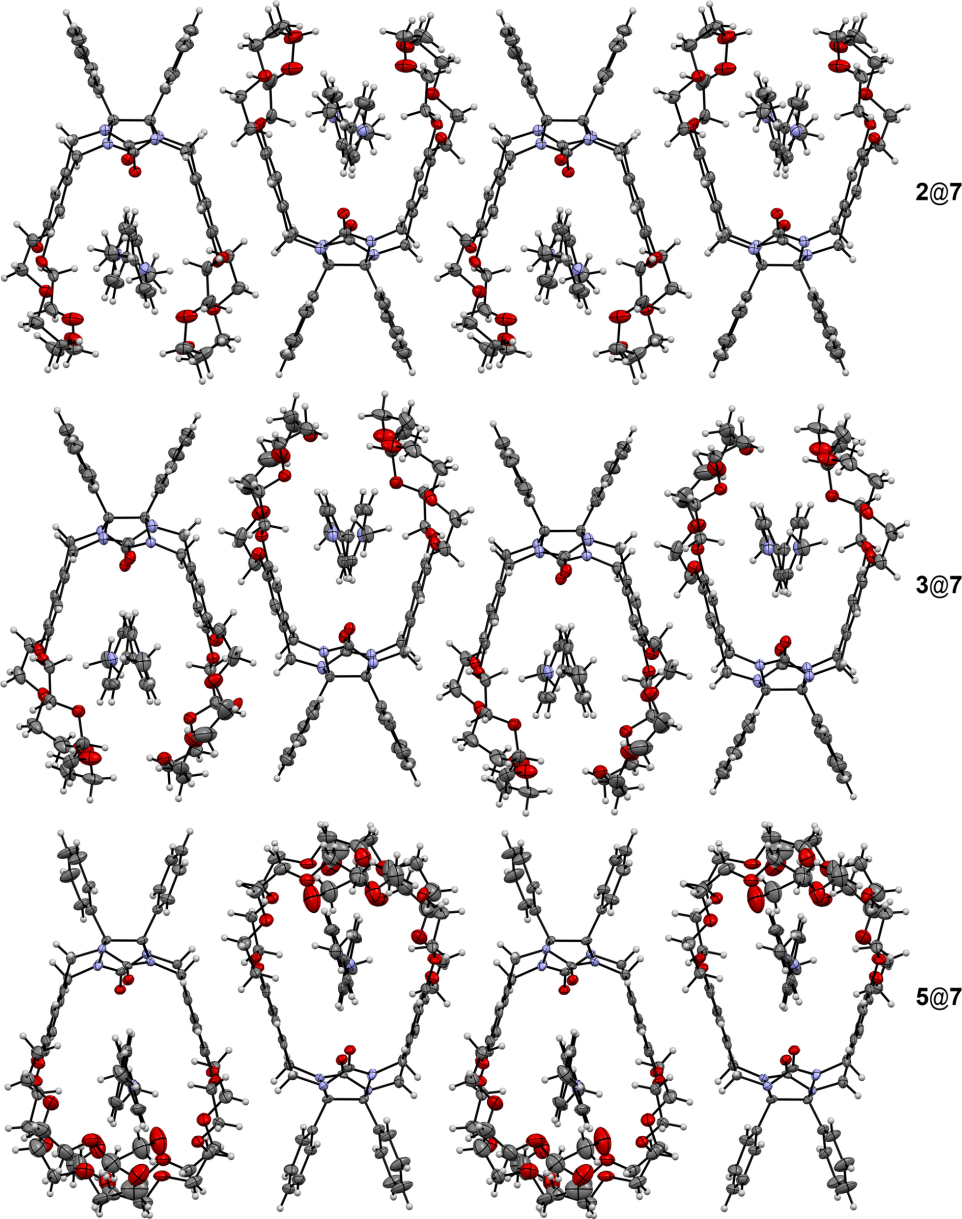
Crystal packing of molecules in complexes of clips **2**, **3** and **5** with paraquat (**7**). Anions and solvate molecules are omitted for clarity.

In the crystal phase molecules form infinite rows in such way that the neighboring molecules are turned to each other on 180° within the row (Figure 7).

Within the row molecules are arranged by π–π stacking interactions between the side aromatic fragments of the clips with a centroid–centroid distance in the range of 3.51–3.71 Å. There are acetonitrile solvate molecules and hexafluorophosphate anions between neighboring rows.

Quantum chemical calculations show that the substitution of methyl groups in the molecule of paraquat by oxyethylene linker results in a decrease of the positive charge on the dipyridinium fragment (Figure 2) that, in turn, should lead to a reduction in the stability of the inclusion complexes of **8**–**10** with molecular clips **1**–**5** by weakening of π–π stacking and C–H···O interactions [36]. At the same time, the presence of terminal hydroxy groups in compounds **8**–**10** may increase the stability of complexes by forming additional hydrogen bonds between molecular clips and hydroxy groups of the substituted dipyridinium salts [37–40].

The stability constants of clips **1**–**5** and paraquat derivatives **7**–**10** in acetonitrile are shown in Figure 5 and Table 2. For the most complexes of clips **1**–**5** with paraquat derivatives **7**–**10** stability constants *K*_2_ have been found to vary in the range of 7–10 except for **3**@**10** with *K*_2_ equal to 49. Substitution of the methyl group in the **7** by ethylene or diethylene glycol fragments leads to an increase in stability of the complexes except **5**@**8** and **5**@**9** (Figure 5). The complex stabilities of the clips **1**–**3** with paraquat derivative **10** are similar compared to complexes with paraquat (**7**) (**1**@**10**) or slightly lower (**2**@**10**, **3**@**10**).

Since a decrease of the positive charge on the dipyridinium fragments (Figure 2) was observed for paraquat derivatives **8** and **9**, it would be logical to assume that the increase of the complex stability is caused by the formation of additional hydrogen bonds involving the substituents on the nitrogen atoms, including terminal hydroxy groups. The fall in complex stability of clips **1**–**3** with **10** probably occurs due to the fact that longer oxyethylene chains form less energetically favorable hydrogen bonds. The experimental data obtained on the stability of molecular clips **1**–**5** with paraquat (**7**) in solution is well correlated with the data for these complexes in the crystalline state revealed by quantum chemical calculations [41] except complexes with paraquat derivative **10** (Table 4).

**Table 4:** The host–guest interaction energies are estimated by quantum chemical calculations (b97-D3/def2-tzvp method) taking BSSE correction into account. Geometries of the complexes have been obtained from X-ray data.

Complex	**2**@**7**	**2**@**8**	**2**@**9**	**2**@**10**	**3**@**7**	**3**@**8**	**3**@**9**	**3**@**10**	**5**@**7**

−*E*_int_, kcal/mol	26.20	29.98	40.84	32.72	26.97	39.37	40.48	41.38	43.79

The calculated data show that the main contribution to the stabilization of the complexes in the crystalline state is made by dispersive interactions (Table S2, Supporting Information File 1). Since the constant stability values of the complexes **6**@**8**–**6**@**10** were below the detection threshold of the method used, we were unable to determine the contribution of the crown ether fragments to the stability of the complexes with paraquats **8**–**10**. Nevertheless, we have demonstrated qualitatively the complex formation of **6**@**8**–**6**@**10** by FAB–MS (Figures S43-S45, Supporting Information File 1).

**Figure 8:**
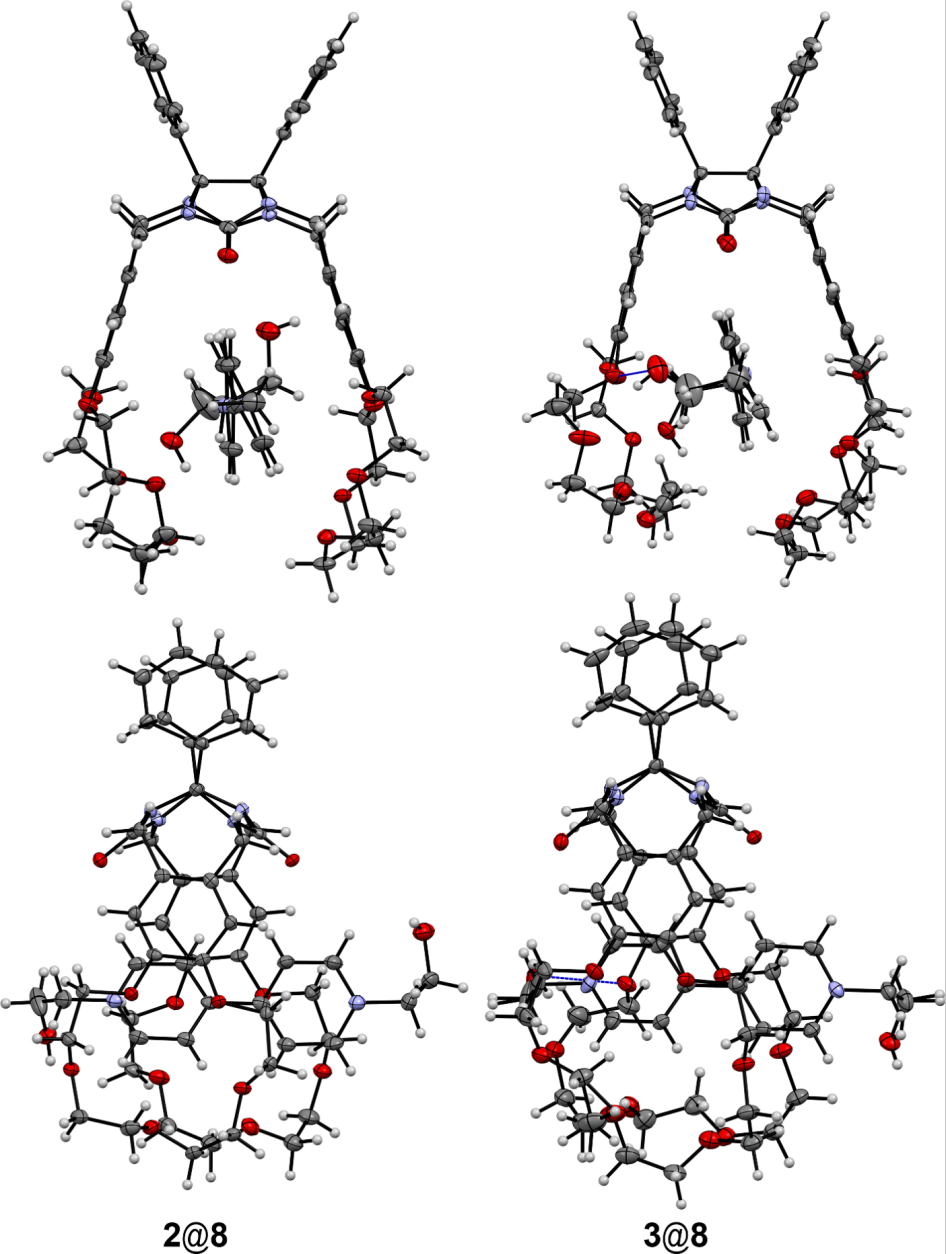
Molecular structures of complexes **2**@**8** and **3**@**8**. The hydrogen bonds are shown by blue lines. Anions and solvate molecules are omitted for clarity.

**Figure 9:**
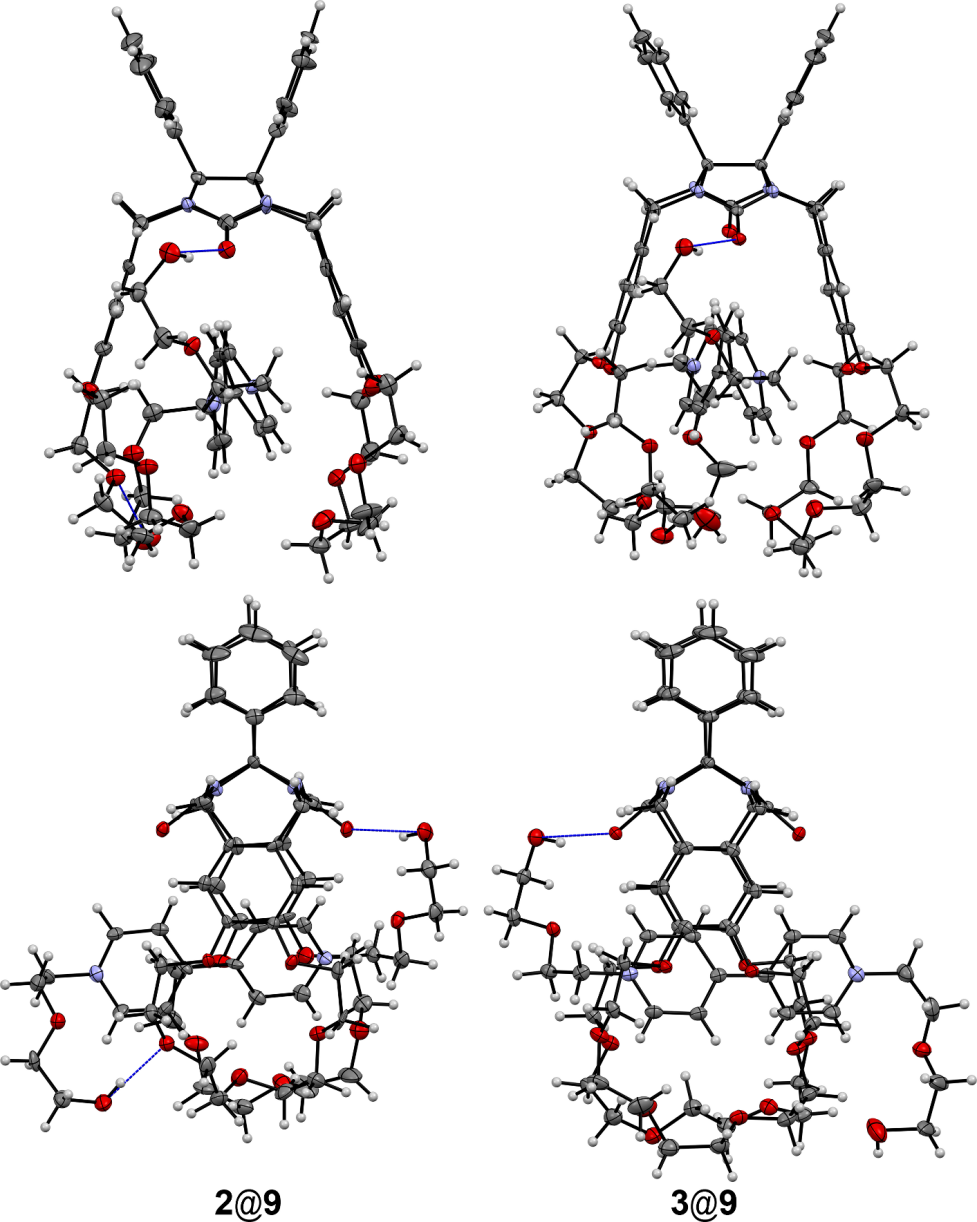
Molecular structures of complexes **2**@**9** and **3**@**9**. The hydrogen bonds are represented by blue lines. Anions and solvate molecules are omitted for clarity.

There are no hydrogen bonds between the protons of the terminal hydroxy groups and oxygen atoms in the complex of clip **2** with guest **8** in the crystals. Meanwhile the hydrogen bond between one hydroxy group of cation **8**^2+^ and an oxygen atom of the polyether chain of clip **3** has been observed. The inclusion complexes of clips **2** and **3** with cations **8**^2+^ and **9**^2+^ have 1:1 composition in the crystal phase (Figure 8 and Figure 9).

Similar to complexes with paraquat (**7**), the host–guest complexes **2**@**8** and **3**@**8** are stabilized by π–π stacking, C–H···O and C–H···π interactions. So far as the structures of these complexes are very similar the main attention will be paid to some differences only. The presence of the terminal hydroxy groups creates the capability of additional stabilization of the complexes of the clips with paraquat derivatives due to the formation of the hydrogen bonds. Indeed, such an hydrogen bond is observed between one hydroxy group of cation **8**^2+^ and the polyether oxygen atom of clip **3** (О–H···O, O···O, О–H···O, 2.14, 2.97 Å, 170.2°). But a similar hydrogen bond is absent in the complex **2**@**8**. One hydrogen bond is formed by the hydroxy group of **9** and the carbonyl oxygen atom of the diphenylglycoluril fragment of the clip (О–H···O, O···O, О–H···O, 2.02, 2.85 Å, 170.1° for **2** and 2.06, 2.86 Å, 161.3° for **3**) in the complexes **2**@**9** and **3**@**9**. The other hydroxy group of **9** participates in the formation of the hydrogen bond with the polyether oxygen atom of clip **2** within this complex while the intermolecular hydrogen bond between such a hydroxy group and the polyether oxygen atom of the clip belonging to the neighboring complex was found in the crystals of **3**@**9**. In the crystal phase these complexes form the infinite rows that consist of alternate molecules inverted to each other as it was found for the complexes of clips with paraquat (**7**).

**Figure 10:**
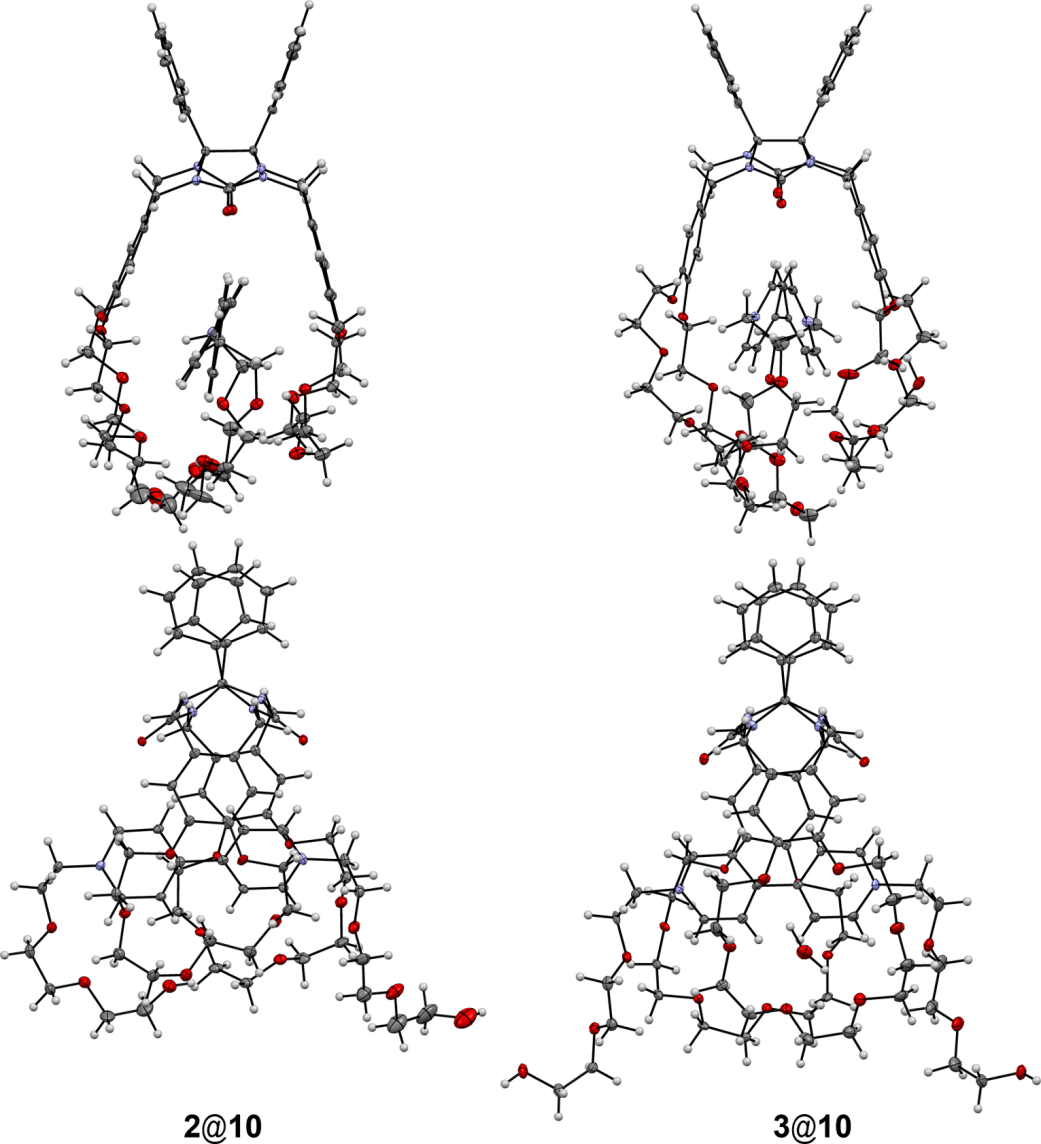
Molecular structures of complexes **2**@**10** and **3**@**10**. Anions and solvate molecules are omitted for clarity.

The structures of the complexes **2**@**10** and **3**@**10** have essential distinctions (Figure 10).

For example, the intramolecular hydrogen bonds with participation of the carbonyl oxygen atom which are typical for complexes **2**@**9** and **3**@**9** are not formed. In complex **2**@**10** one hydroxy group forms an intramolecular hydrogen bond with a polyether oxygen atom of the clip (О–H···O, O···O, О–H···O, 2.29, 2.99 Å, 141°). The oxygen atom of the second hydroxy group acts as proton acceptor and forms the weak C–H···О hydrogen bonds with CH_2_ and H_α_ hydrogen atoms of **10** belonging to the neighboring complex (C–H···O, C···O, C–H···O, 2.59, 3.27 Å, 126.3°; 2.67, 3.30 Å, 124.5°).

The crystal packing is similar to the one described above for other complexes. The π–π stacking interactions between the side aromatic fragments have the major contribution in the mutual disposition of studied complexes. A lot of hydrogen bonds have been found between neighboring complexes or between complexes and solvate molecules and counterions.

In the case of complex **3**@**10** both crown-ether cavities are occupied by water molecules. Each of these water molecules form two strong hydrogen bonds with polyether oxygen atoms (О–H···O, O···O, О–H···O, 1.87, 2.95 Å, 179.1°; 2.00, 3.08 Å, 179.5°; 1.94, 3.02 Å, 178.9°; 1.82, 2.90 Å, 179.4°). Moreover, some of the water molecules participate as a proton acceptor in the formation of the C–H···О hydrogen bonds with the methylene hydrogen atoms of the glycoluril fragment of neighboring molecules (C–H···O, C···O, C–H···O, 2.34, 3.25 Å, 155°; 2.59, 3.45 Å, 149°; 2.56, 3.26 Å, 130°). The hydroxy groups of **10** participate in the intermolecular interactions with the carbonyl oxygen atoms of neighboring molecules only forming two hydrogen bonds (О–H···O, O···O, О–H···O, 2.51, 2.77 Å, 99.1° and 1.98, 2.80 Å, 174.1°). As a result, the crystal packing of this complex differs from the described above. The molecules within the infinite rows are bonded by the hydrogen bonds rather than π–π stacking interactions.

## Conclusion

Thus, the data obtained allows us to conclude that molecular clips based on diphenylglycoluril and benzocrown ethers form inclusion complexes with paraquat (**7**) preferably of 1:1 composition. The stability constants with paraquat (**7**) rise with the increase of the polyether cycle size. The maximum complementarity of the pseudo cavity for binding of paraquat (**7**) was observed for clips based on benzo-21-crown-7 and benzo-24-crown-8. The introduction of substituents with terminal OH groups on the nitrogen atoms of paraquat usually increases the stability of complexes formed, which is caused by their additional stabilization due to hydrogen bonds between the terminal hydroxy groups and oxygen atoms of the polyether chains, and the carbonyl groups of the molecular clips. In the solid state, the studied clips form inclusion complexes of 1:1 composition.

## Supporting Information

File 1 Experimental section, complete X-ray data, ^1^H NMR and FAB–MS spectra are provided.
