# Supplementary material for: Complexation of molecular clips containing fragments of diphenylglycoluril and benzocrown ethers with paraquat and its derivatives
Source: Beilstein J Org Chem. 2017 Oct 4;13:2056–67. doi: 10.3762/bjoc.13.203 (PMC5647707; doi:10.3762/bjoc.13.203)
Supplement: File 1 — Experimental section, complete X-ray data, 1H NMR and FAB–MS spectra are provided. [file Beilstein_J_Org_Chem-13-2056-s001.pdf]

Supporting Information

for

Complexation of molecular clips containing fragments of diphenylglycoluril and benzocrown ethers with paraquat and its derivatives

Leonid S. Kikot'[1], Catherine Yu. Kulygina[1], Alexander Yu. Lyapunov[1], Svetlana V. Shishkina[2,3], Roman I. Zubatyuk[2], Tatiana Yu. Bogaschenko[1] and Tatiana I. Kirichenko[1]*

Address: [1]Department of Fine Organic Synthesis, A.V. Bogatsky Physico-Chemical Institute, National Academy of Sciences of Ukraine, Lustdorfskaya doroga 86, Odesa 65080, Ukraine, [2]Department of X-ray Diffraction Studies and Quantum Chemistry, SSI ‘‘Institute for Single Crystals’’, National Academy of Sciences of Ukraine, Nauky Ave. 60, Kharkiv 61001, Ukraine and [3]Department of Inorganic Chemistry, V.N. Karazin Kharkiv National University, 4 Svobody Sq., 61122, Kharkiv, Ukraine

Email: Tatiana I. Kirichenko - Ti.kirichenko@ukr.net

* Corresponding author

Experimental section, complete X-ray data, ^{1}H NMR and FAB–MS spectra are provided

General

UV–vis absorption spectra were obtained using a Carl Zeiss Specord M40 spectrophotometer. ^{1}H NMR spectra were obtained on a Varian VXR-300 (300 MHz) spectrometer. All chemical shifts are quoted in ppm on the δ scale with TMS as an internal standard. Mass spectra were obtained on VG 70-70EQ (FAB-MS, Xe, 8 kV, m-nitrobenzyl alcohol matrix) spectrometer. Molecular clips **1**–**5** [S1], **6** [S2] and guests **7**–**10** [S3-S5] were prepared as described.

UV–Vis titration experiments

A solution of molecular clip **1**–**6** (concentration about 1×10^{-4}–1×10^{-3} M) in acetonitrile was treated with increasing amounts of guests **7**–**10** solution (concentration about 1×10^{-3}–1×10^{-2} M) containing proper molecular clip of the same concentration at 20 °C. The host concentration was maintained constant and the molar ratio of guest increased with respect to the host over the range 0.1:1–40:1 during the titration. The absorbance measurements were carried out at four wavelengths, at which spectral changes were the most notable (360–530 nm) simultaneously, and sets of the obtained experimental values (4 × 18 points) were used for joint computer processing. The data were processed with the nonlinear least squares fitting SIRKO [S6] software.

X-ray crystallography

Crystals of studied complexes suitable for X-ray diffraction have been grown using isopropyl ether diffusion into the solution of their equimolar mixture in acetonitrile. X-Ray diffraction studies have been performed on an automatic “Xcalibur-3” diffractometer (graphite monochromated Mo Kα radiation, CCD-detector, ω-scanning). The structures were solved by direct methods using the SHELXTL package [S7]. The restrictions on

the bond lengths in the disordered fragments (C_{Ar}–C_{Ar} 1.38 Å) were applied for the compounds **2@7**, **2@8**, **3@7**, **3@8**, **3@9**, **5@7**. Positions of the hydrogen atoms were located from electron density difference maps and refined by the "riding" model with $U_{iso} = nU_{eq}$ of the carrier atom (n = 1.5 for methyl and hydroxy groups and n = 1.2 for other hydrogen atoms). The crystallographic data and experimental parameters are listed in Table S1. Final atomic coordinates, geometrical parameters and crystallographic data have been deposited with the Cambridge Crystallographic Data Centre, 11 Union Road, Cambridge, CB2 1EZ, UK (fax: +44 1223 336033; e-mail: deposit@ccdc.cam.ac.uk). The deposition numbers are given in Table S1.

Quantum chemical calculations

The estimation of the interaction energies within the studied complexes was performed using quantum chemical calculations. The geometries of complexes have been obtained from the X-ray data and have not been optimized additionally. The interaction energy values were calculated using the B97-D3/Def2-TZVP density functional method [S8-S10] and corrected for basis set superposition error by counterpoise method [S11]. DFT calculations were performed with GAUSSIAN09 program [S12].

References

S1. Bogaschenko, T. Yu.; Lyapunov, A. Yu.; Kikot', L. S.; Mazepa, A. V.; Botoshansky, M. M.; Fonari, M. S.; Kirichenko, T. I. *Tetrahedron* **2012**, *68*, 4757–4764. doi: 10.1016/j.tet.2012.04.009

S2. Kikot', L. S.; Lyapunov, A. Yu.; Zubatyuk, R. I.; Shishkin, O. V.; Kirichenko T. I. *Synlett* **2012**, *23*, 1897-1900. doi: 10.1055/s-0032-1316569

S3. Shen, Y. X.; Engen, P. T.; Berg, M. A. G.; Merola, J. S.; Gibson H. W. *Macromolecules*, **1992**, *25*, 2786–2788. doi: 10.1021/ma00036a037

S4. Ashton, P. R.; Ballardini, R.; Balzani, V.; Bělohradský, M.; Gandolfi, M. T.; Philp, D.; Prodi, L.; Raymo, F. M.; Reddington, M. V.; Spencer, N.; Stoddart, J. F.; Venturi, M.; Williams, D. J. *J. Am. Chem. Soc.*, **1996**, *118*, 4931–4951. doi: 10.1021/ja954334d

S5. Mirzoian, A.; Kaifer, A. E. *Chem. Eur. J.*, **1997**, *3*, 1052–1058. doi: 10.1002/chem.19970030711

S6. Vetrogon, V. I.; Lukyanenko, N. G.; Schwing-Weill, M.-J.; Arnaud-Neu, F. *Talanta* **1994**, *41*, 2105–2112. doi: 10.1016/0039-9140(94)00187-1

S7. Sheldrick, G. M. *Acta Crystallogr.*, *Sect. A: Found. Adv.* **2008**, *64*, 112–122. doi: 10.1107/S0108767307043930

S8. Grimme, S. *J. Comput. Chem.***2006**, *27*, 1787–1799. doi: 10.1002/jcc.20495

S9. Grimme, S.; Ehrlich, S.; Goerigk, L. *J. Comput. Chem.* **2011**, *32*, 1456–1465. doi: 10.1002/jcc.21759

S10. Grimme, S.; Antony, J.; Ehrlich, S.; Krieg, H. *J. Chem. Phys.* **2010**, *132*, 154104-1–154104-19. doi: 10.1063/1.3382344

S11. Boys, S. F.; Bernardi, F. *Mol. Phys.***1970**, *19*, 553–566. doi: 10.1080/00268977000101561

S12. *Gaussian 09*, revision B.01; Gaussian, Inc.: Wallingford CT, 2010.

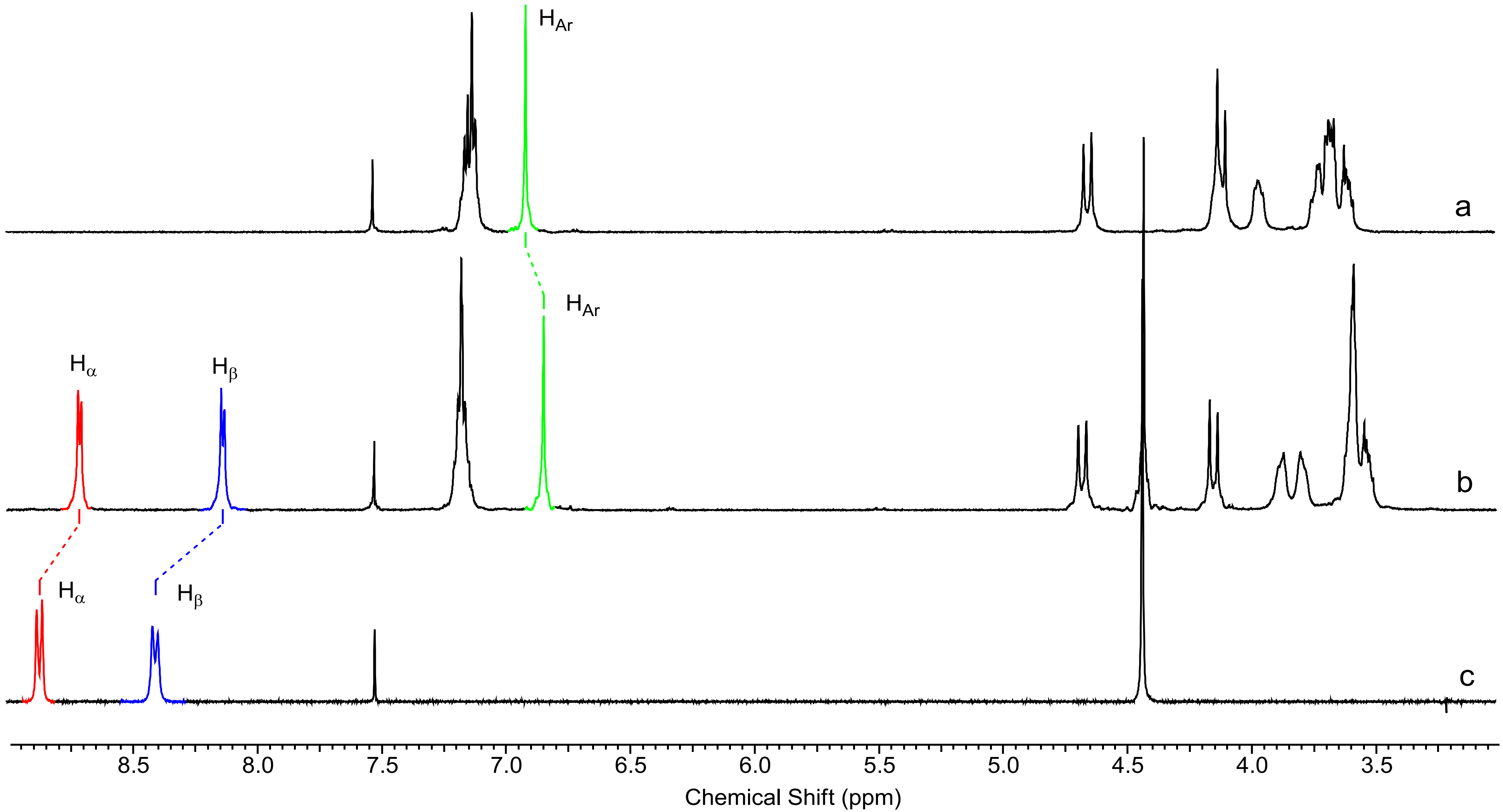

Figure S1: ^{1}H NMR spectra (300 MHz, CD_3CN:$CDCl_3$. 4:3, v/v) of (a) free host **1**, (b) mixture of **1** and **7** (1:1), (c) free guest **7**.

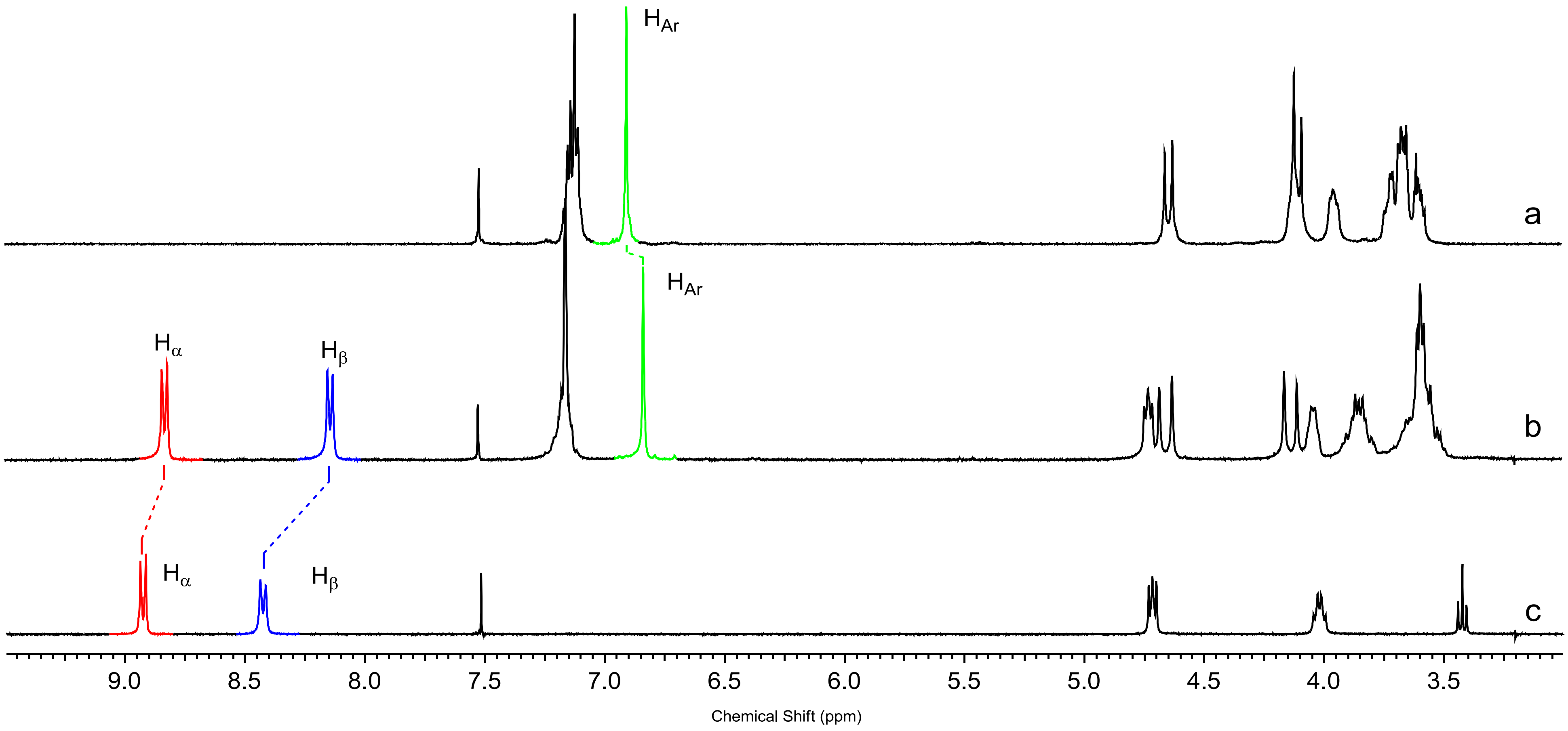

Figure S2: ^{1}H NMR spectra (300 MHz, $CD_3CN:CDCl_3$. 4:3, v/v) of (a) free host **1**, (b) mixture of **1** and **8** (1:1), (c) free guest **8**

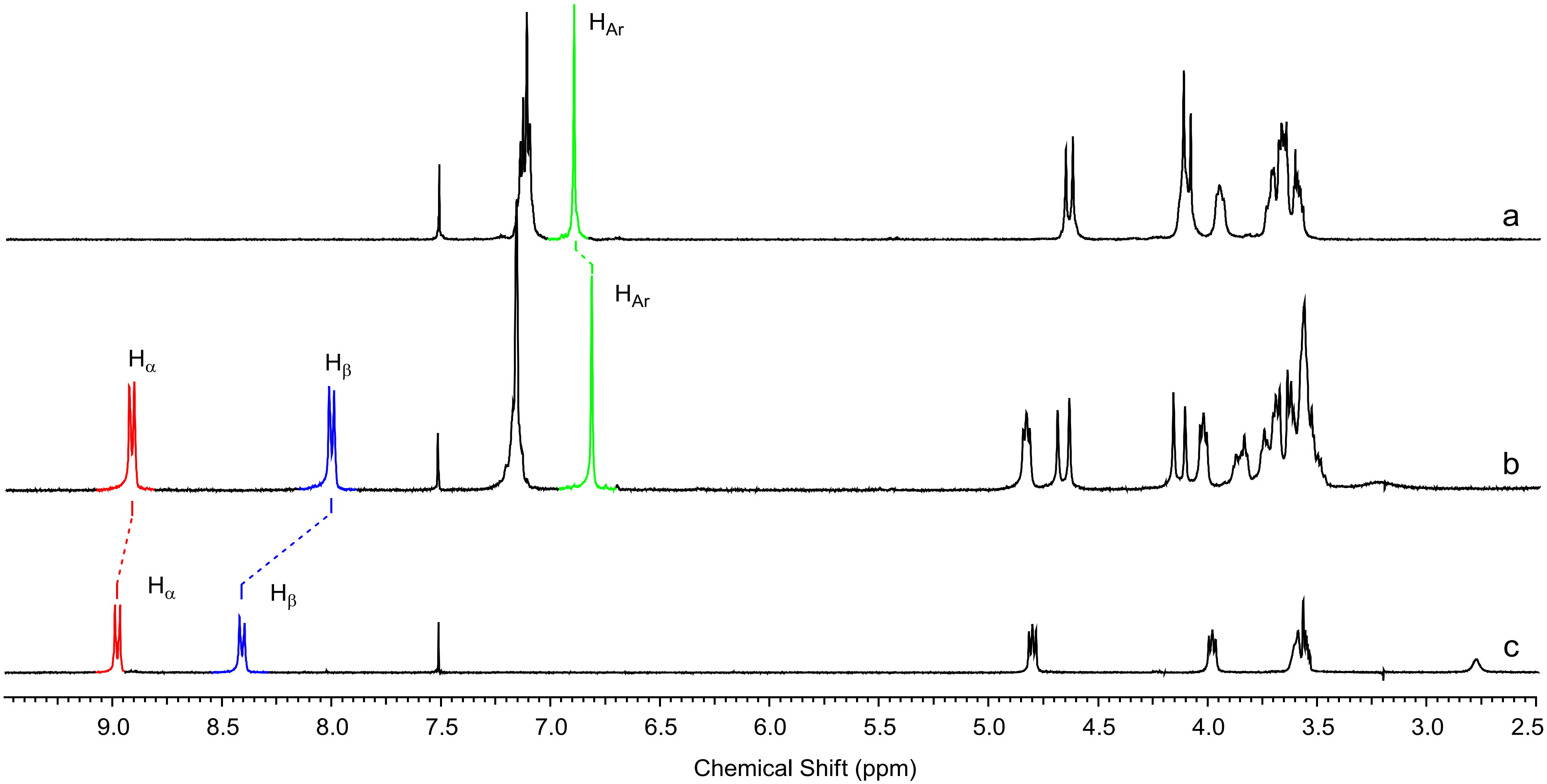

Figure S3: ^{1}H NMR spectra (300 MHz, CD_3CN:$CDCl_3$. 4:3, v/v) of (a) free host **1**, (b) mixture of **1** and **9** (1:1), (c) free guest **9**.

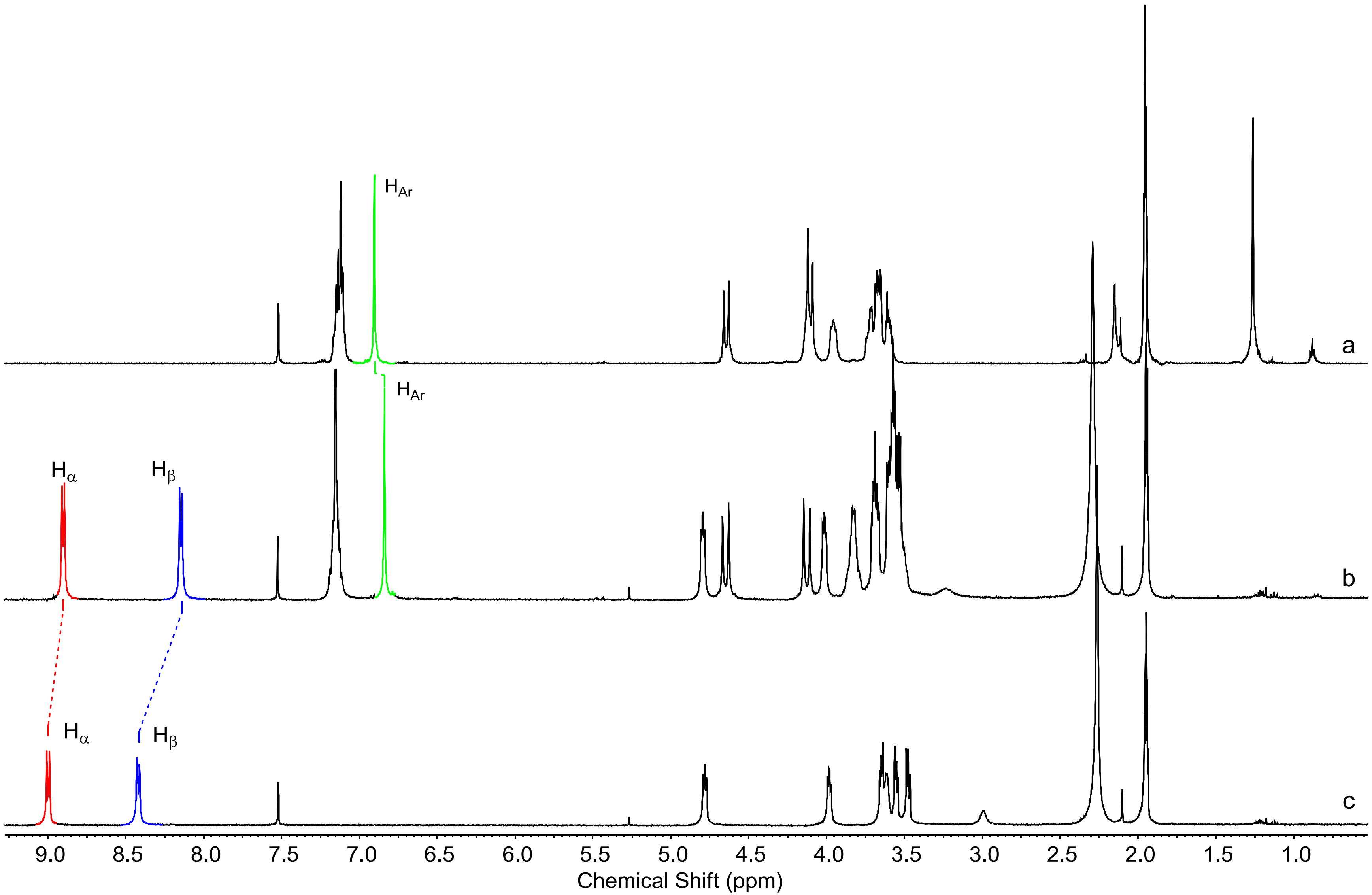

Figure S4 ^{1}H NMR spectra (300 MHz, CD_3CN:$CDCl_3$. 4:3, v/v) of (a) free host **1**, (b) mixture of **1** and **10** (1:1), (c) free guest **10**

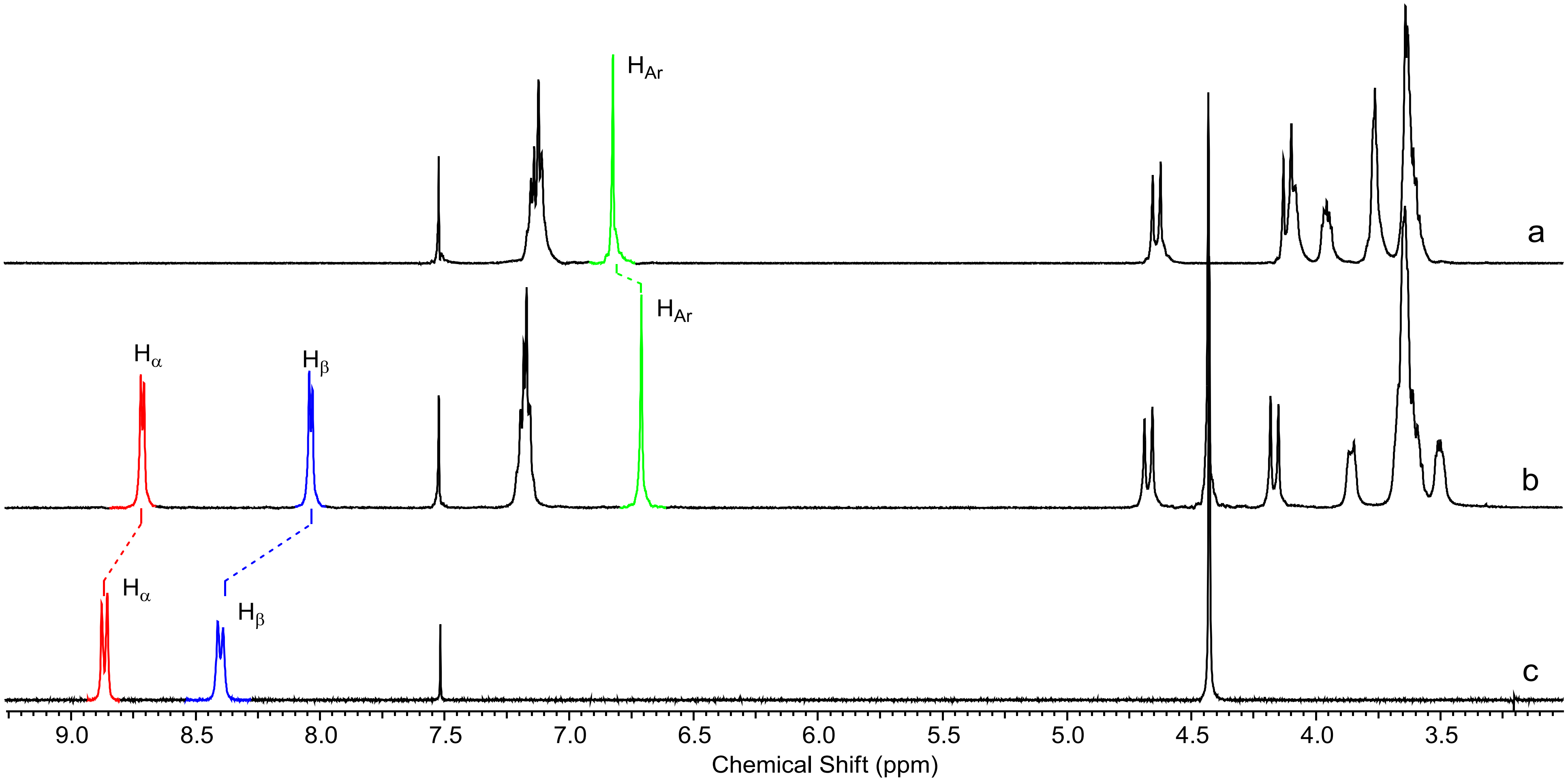

Figure S5: ^{1}H NMR spectra (300 MHz, CD_3CN:$CDCl_3$. 4:3, v/v) of (a) free host **2**, (b) mixture of **2** and **7** (1:1), (c) free guest **7**

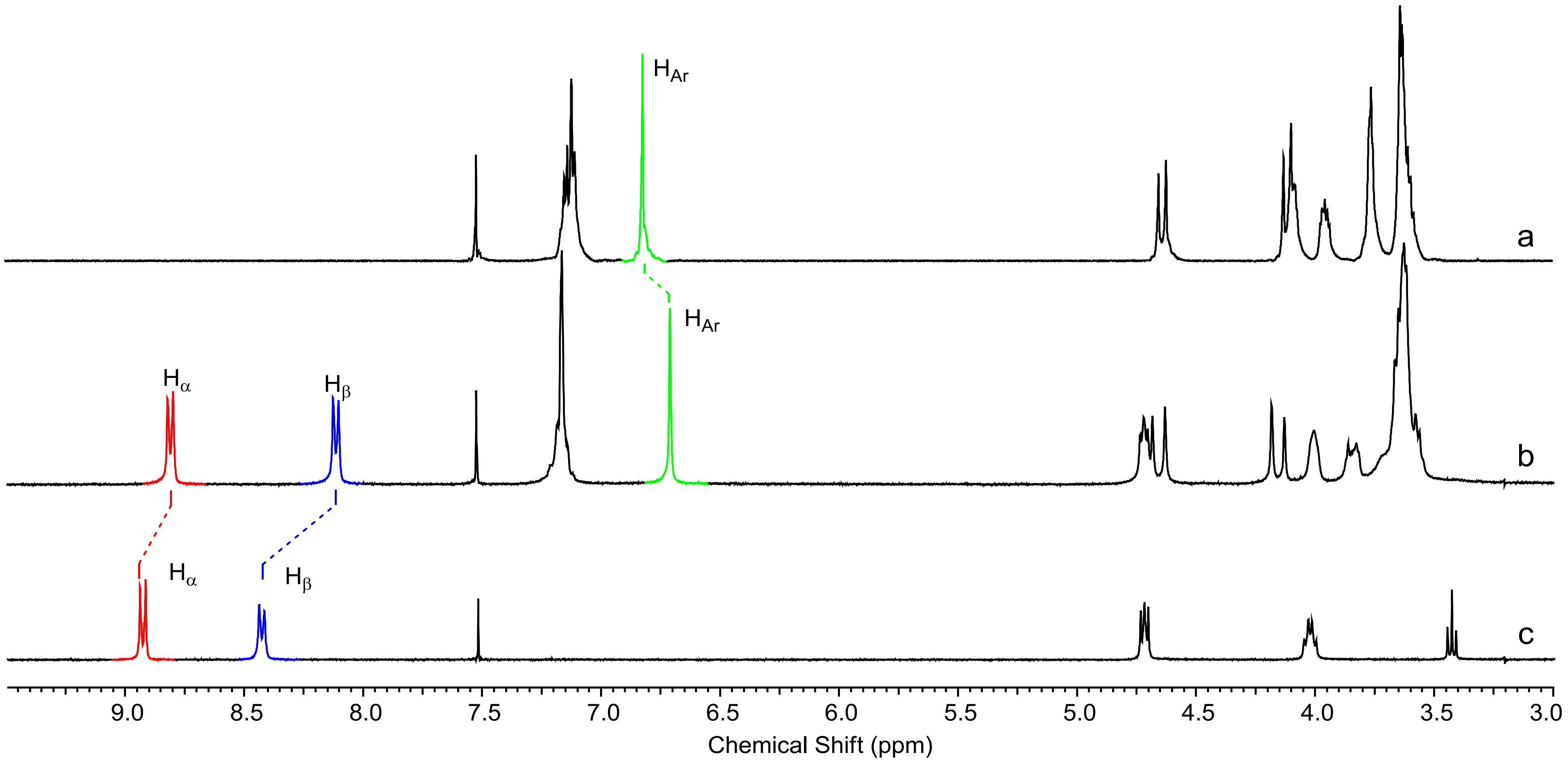

Figure S6: ^{1}H NMR spectra (300 MHz, CD_3CN:$CDCl_3$. 4:3, v/v) of (a) free host **2**, (b) mixture of **2** and **8** (1:1), (c) free guest **8**

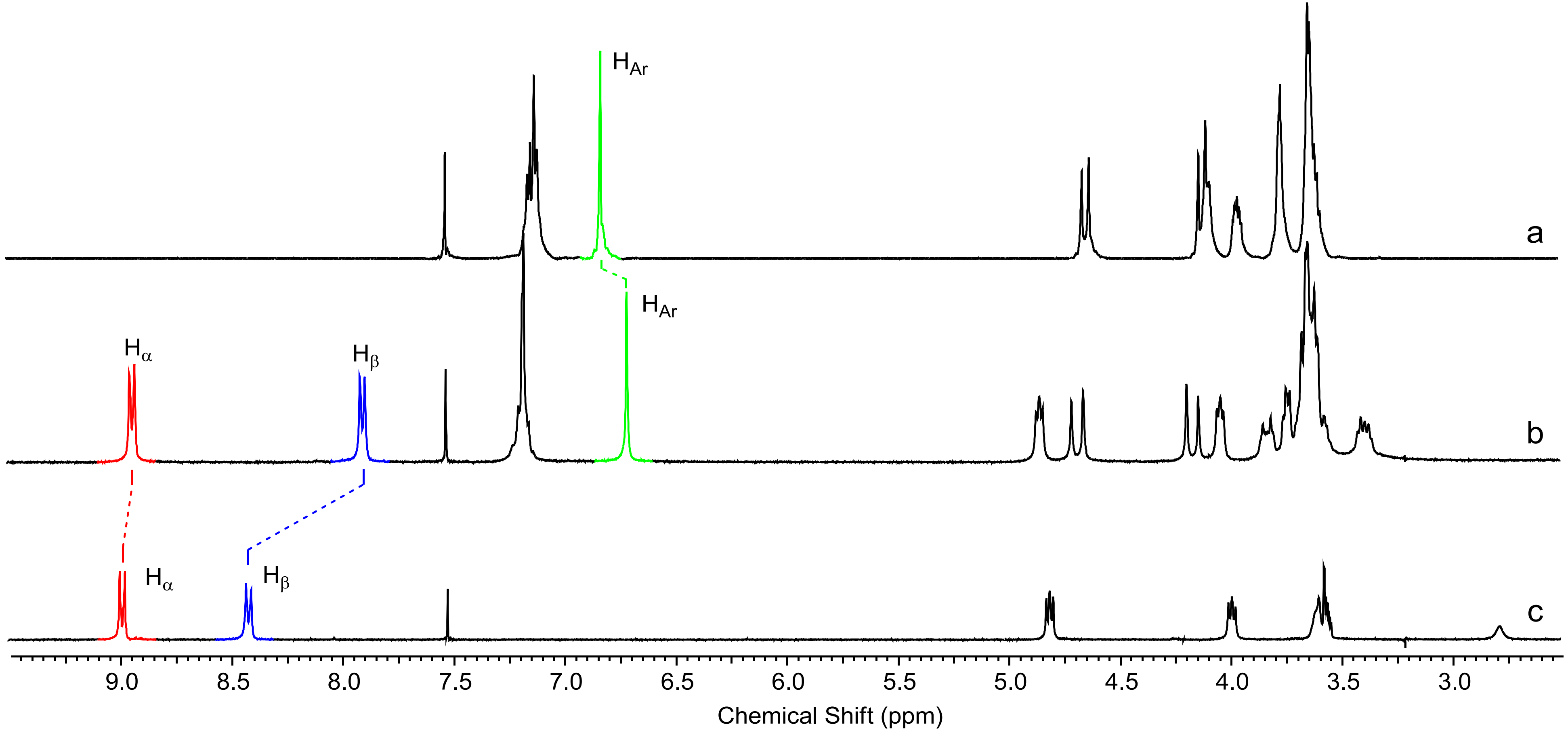

Figure S7: ^{1}H NMR spectra (300 MHz, CD_3CN:$CDCl_3$. 4:3, v/v) of (a) free host **2**, (b) mixture of **2** and **9** (1:1), (c) free guest **9**

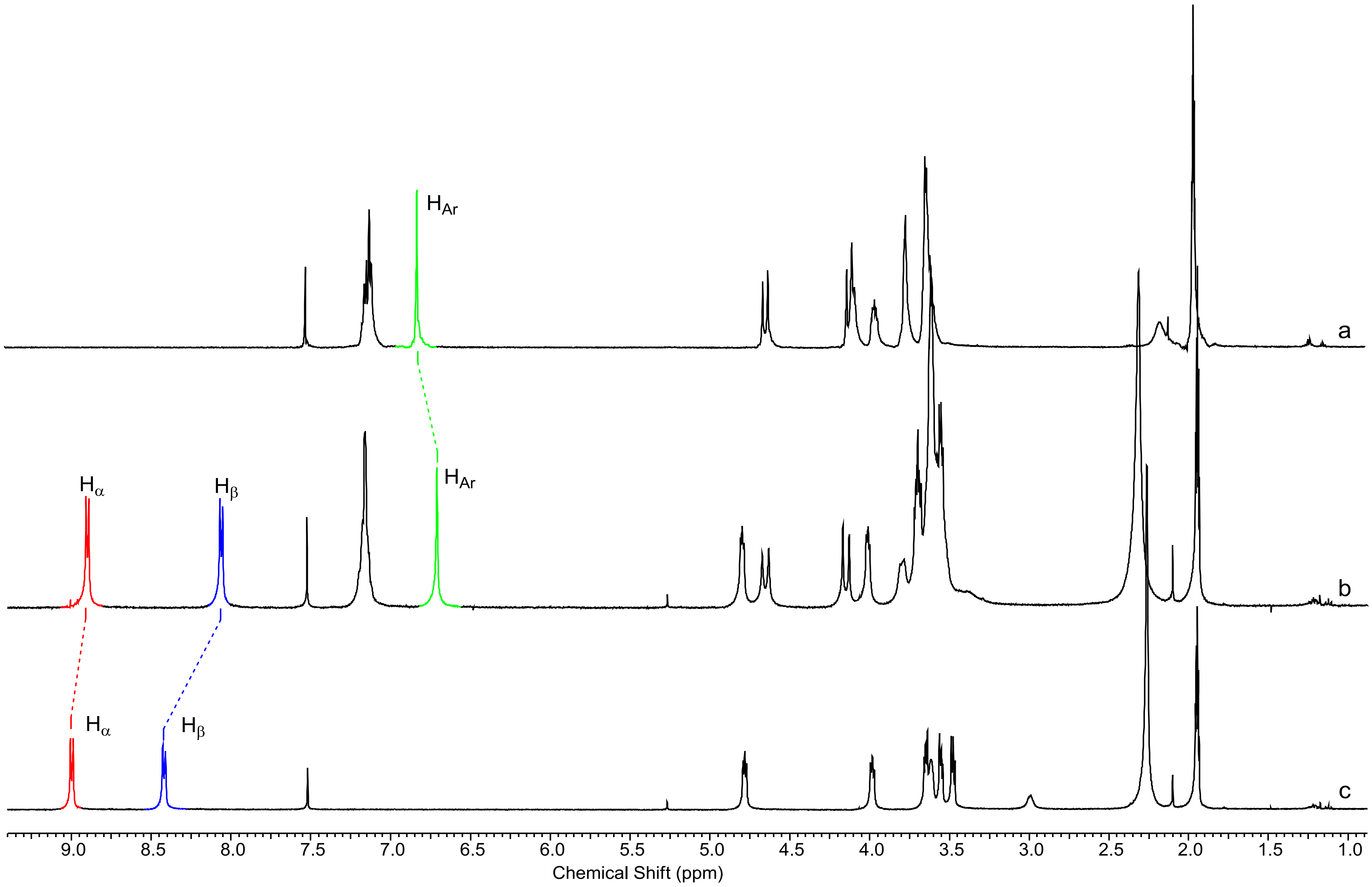

Figure S8: ^{1}H NMR spectra (300 MHz, CD_3CN:$CDCl_3$. 4:3, v/v) of (a) free host **2**, (b) mixture of **2** and **10** (1:1), (c) free guest **10**

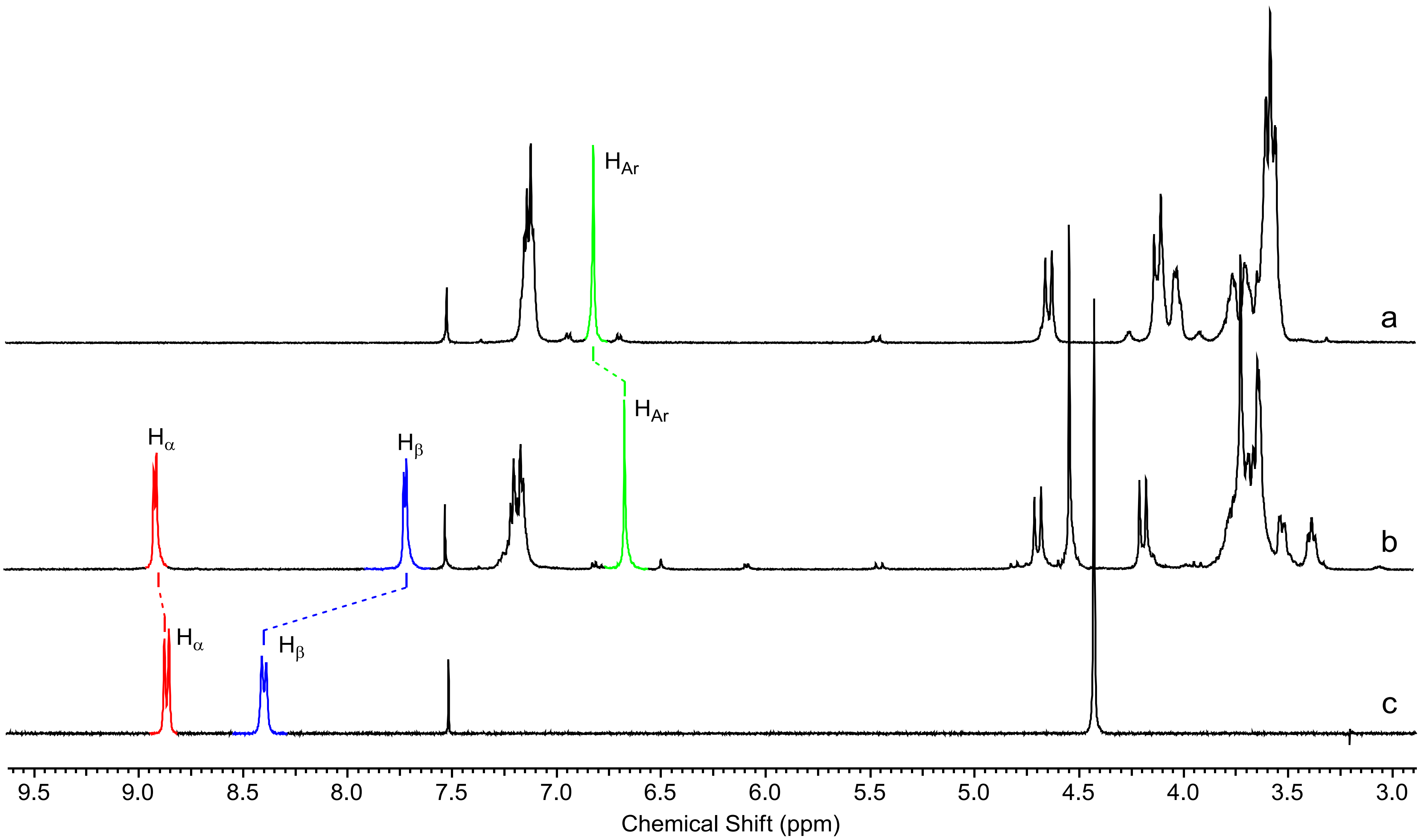

Figure S9: ^{1}H NMR spectra (300 MHz, CD_3CN:$CDCl_3$. 4:3, v/v) of (a) free host **3**, (b) mixture of **3** and **7** (1:1), (c) free guest **7**

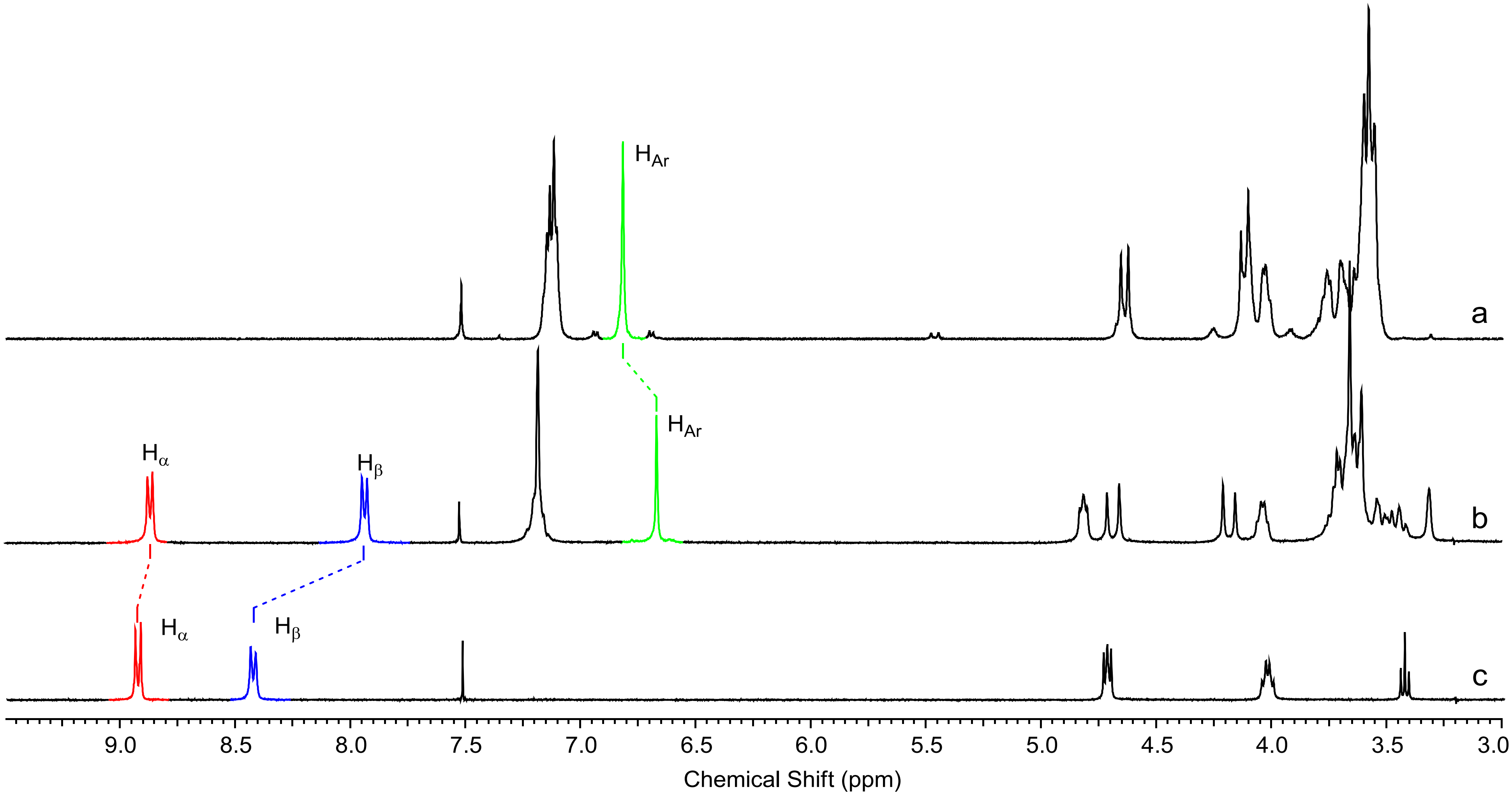

Figure S10: ^{1}H NMR spectra (300 MHz, CD_3CN:$CDCl_3$. 4:3, v/v) of (a) free host **3**, (b) mixture of **3** and **8** (1:1), (c) free guest **8**

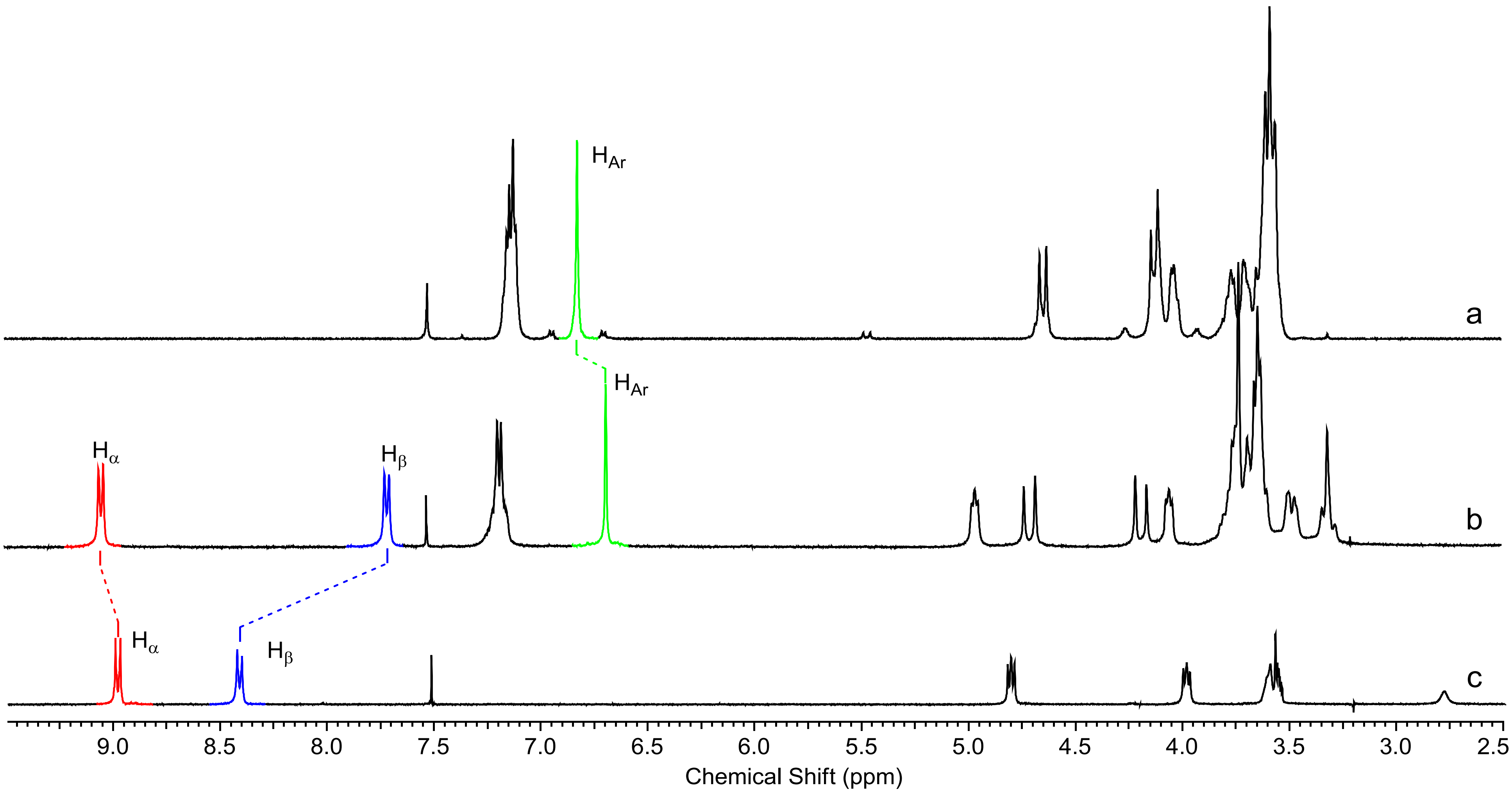

Figure S11: ^{1}H NMR spectra (300 MHz, $CD_3CN:CDCl_3$. 4:3, v/v) of (a) free host **3**, (b) mixture of **3** and **9** (1:1), (c) free guest **9**

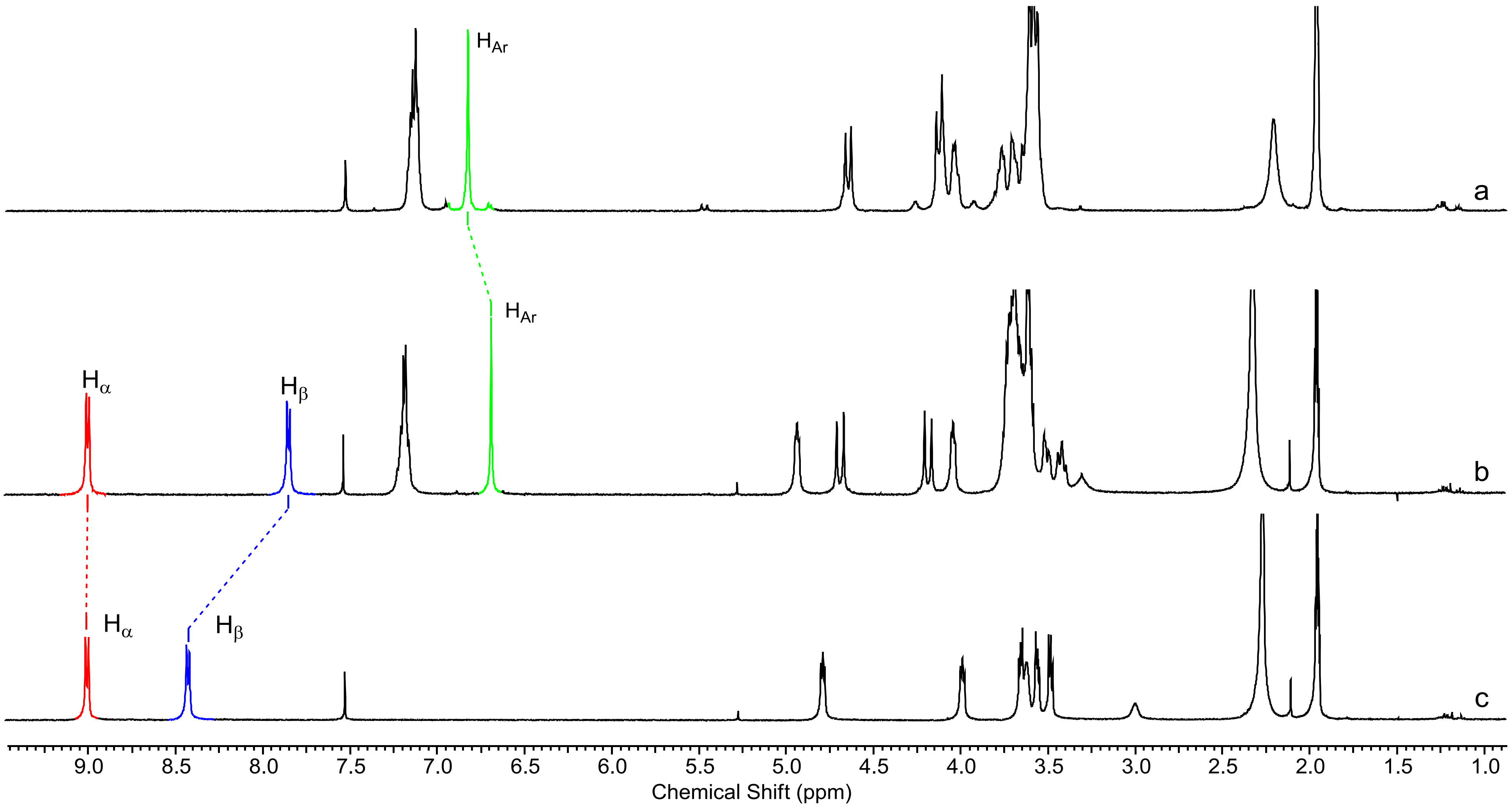

Figure S12: ^{1}H NMR spectra (300 MHz, $CD_3CN:CDCl_3$. 4:3, v/v) of (a) free host **3**,(b) mixture of **3** and **10** (1:1), (c) free guest **10**

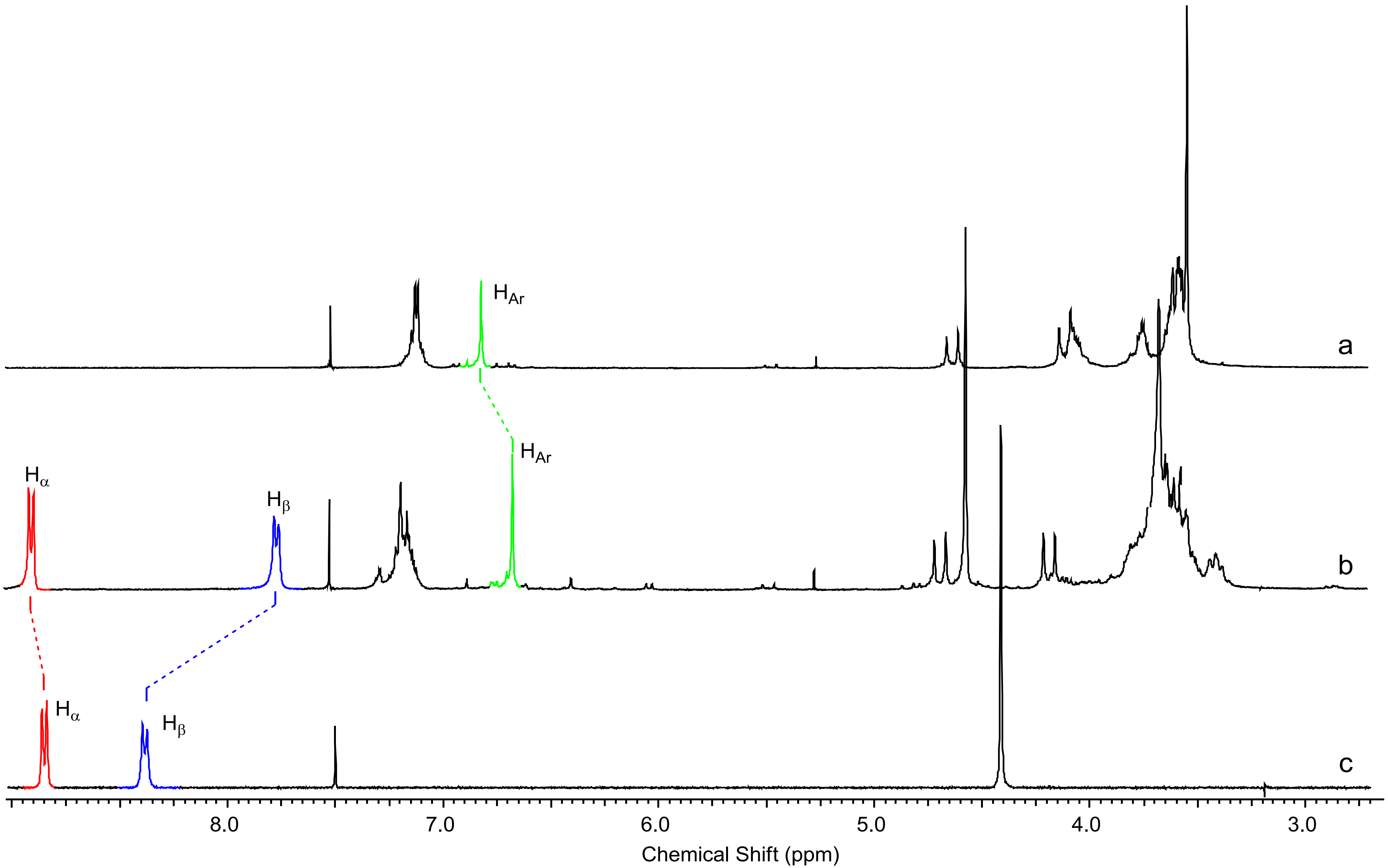

Figure S13: ^{1}H NMR spectra (300 MHz, CD_3CN:$CDCl_3$. 4:3, v/v) of (a) free host **4**, (b) mixture of **4** and **7** (1:1), (c) free guest **7**

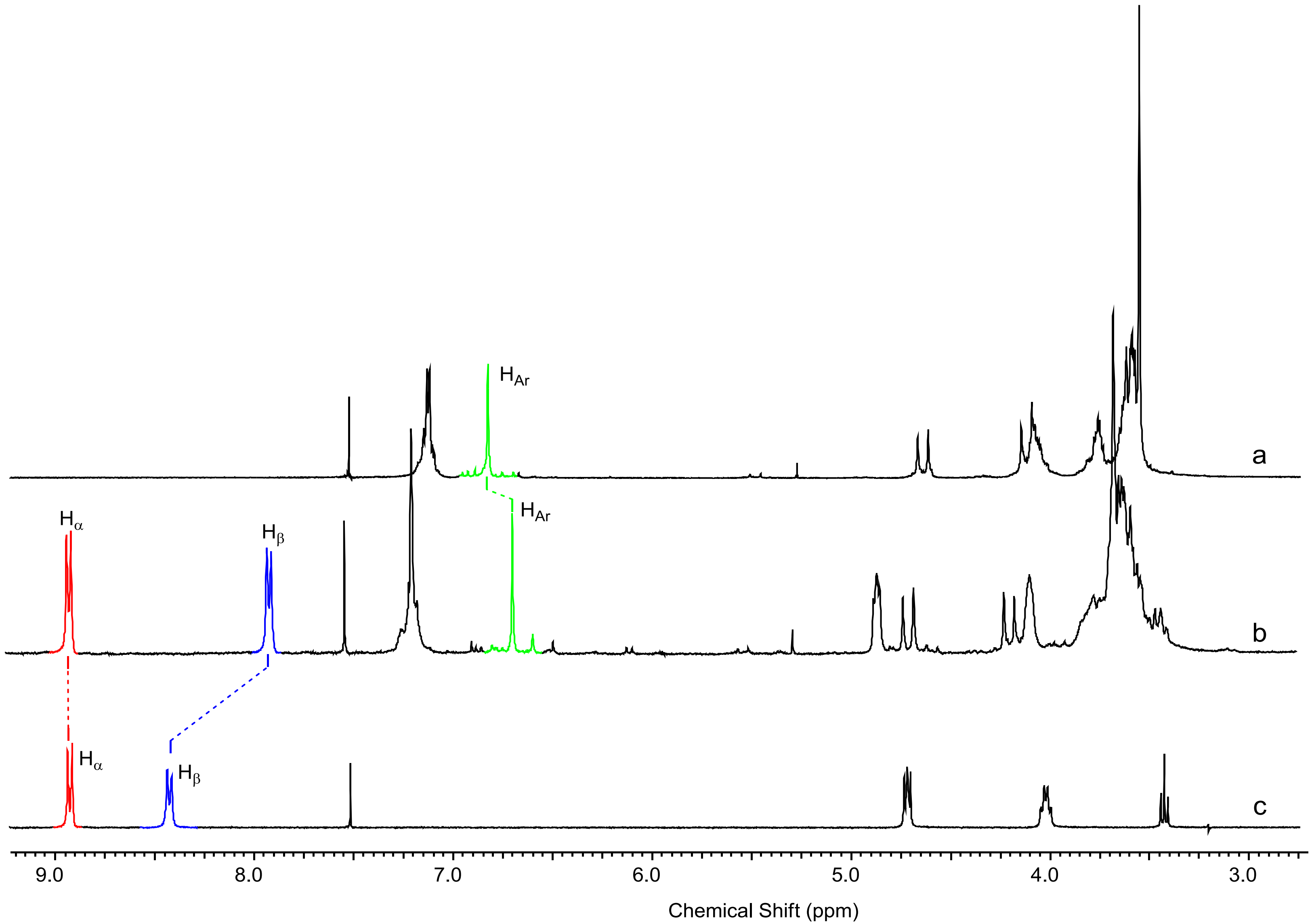

Figure S14: ^{1}H NMR spectra (300 MHz, CD_3CN:$CDCl_3$. 4:3, v/v) of (a) free host **4**, (b) mixture of **4** and **8** (1:1), (c) free guest **8**

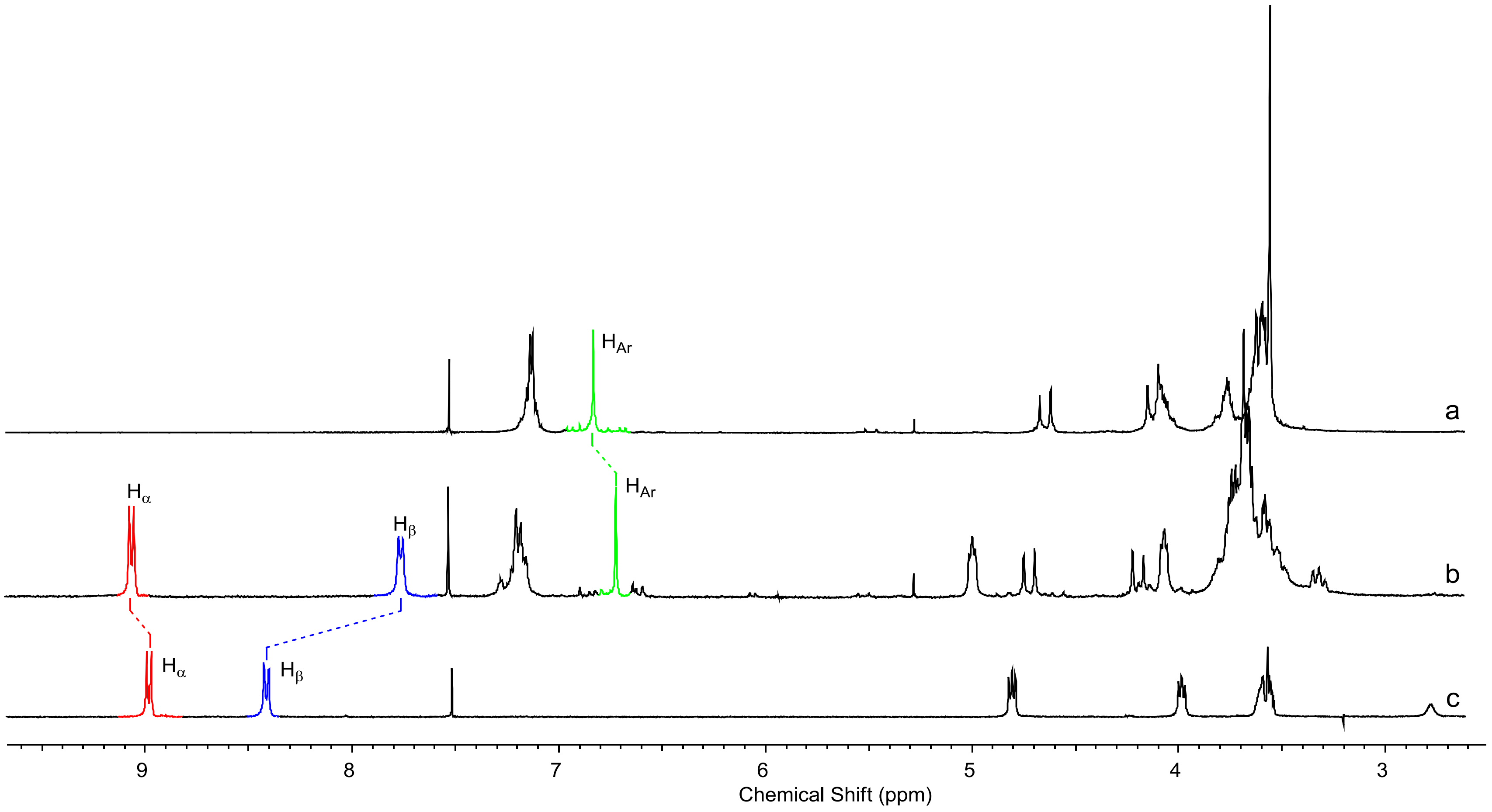

Figure S15: ^{1}H NMR spectra (300 MHz, CD_3CN:$CDCl_3$. 4:3, v/v) of (a) free host **4**, (b) mixture of **4** and **9** (1:1), (c) free guest **9**

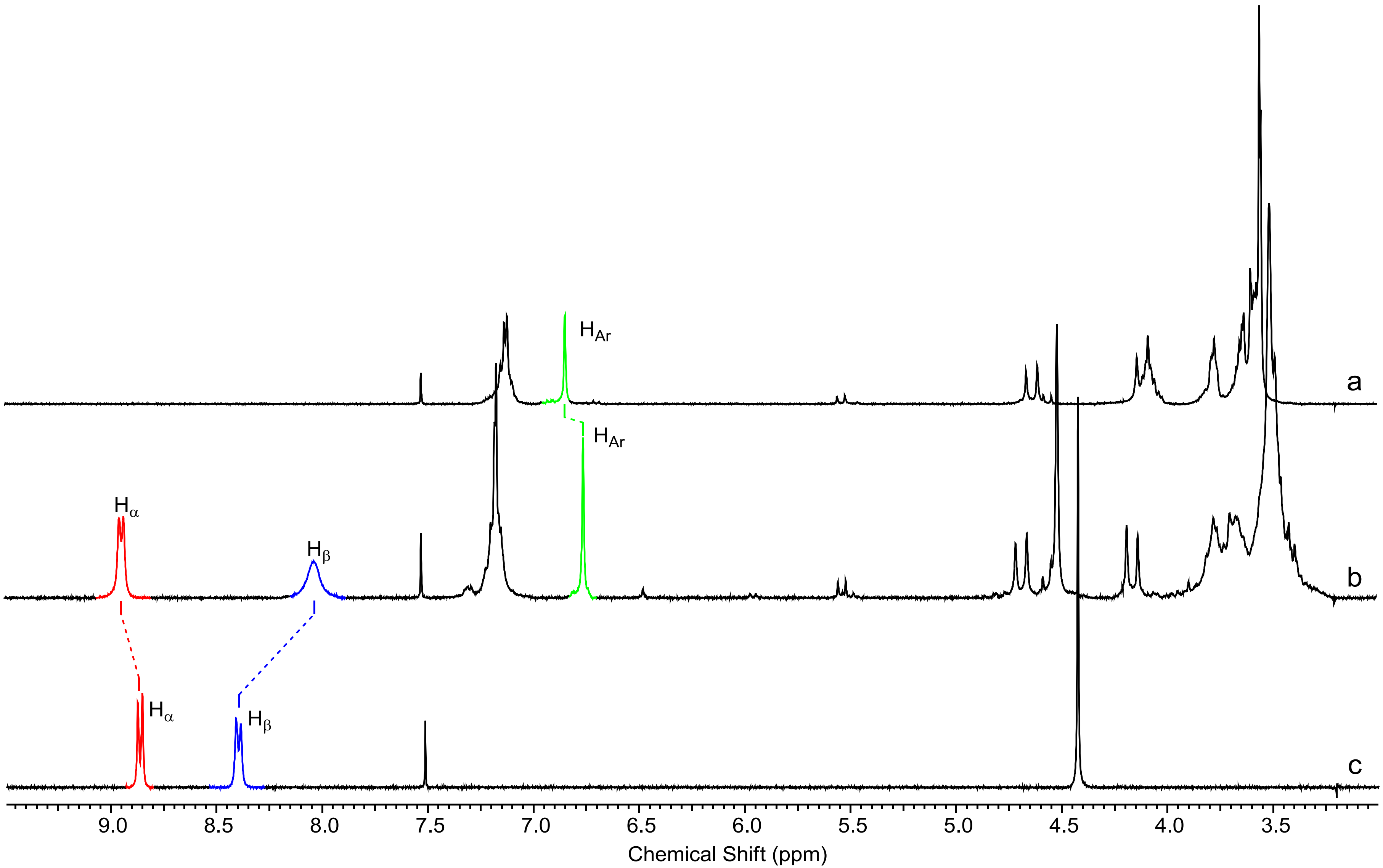

Figure S16: ^{1}H NMR spectra (300 MHz, CD_3CN:$CDCl_3$. 4:3, v/v) of (a) free host **5**, (b) mixture of **5** and **7** (1:1), (c) free guest **7**

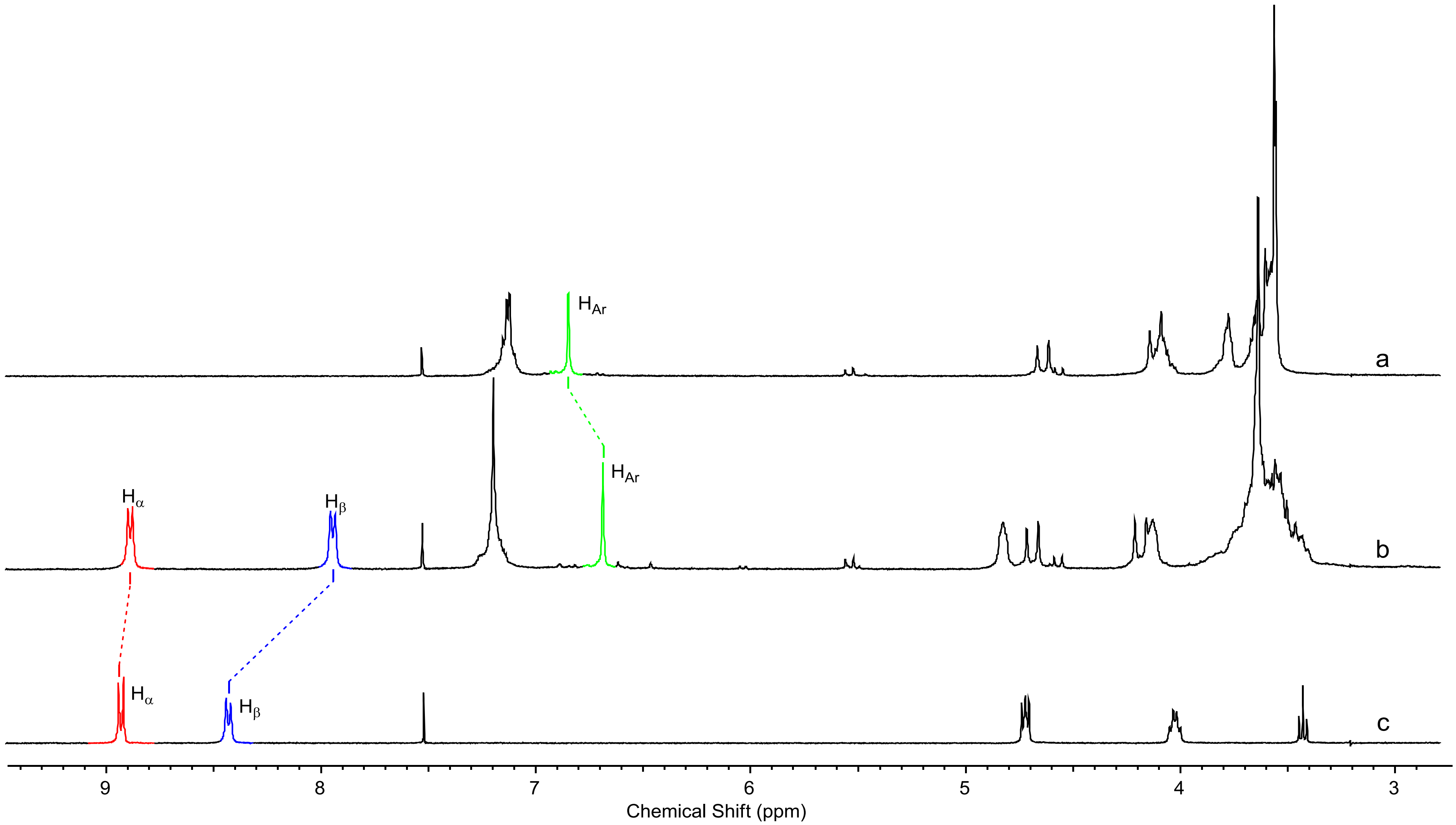

Figure S17: ^{1}H NMR spectra (300 MHz, CD_3CN:$CDCl_3$. 4:3, v/v) of (a) free host **5**, (b) mixture of **5** and **8** (1:1), (c) free guest **8**

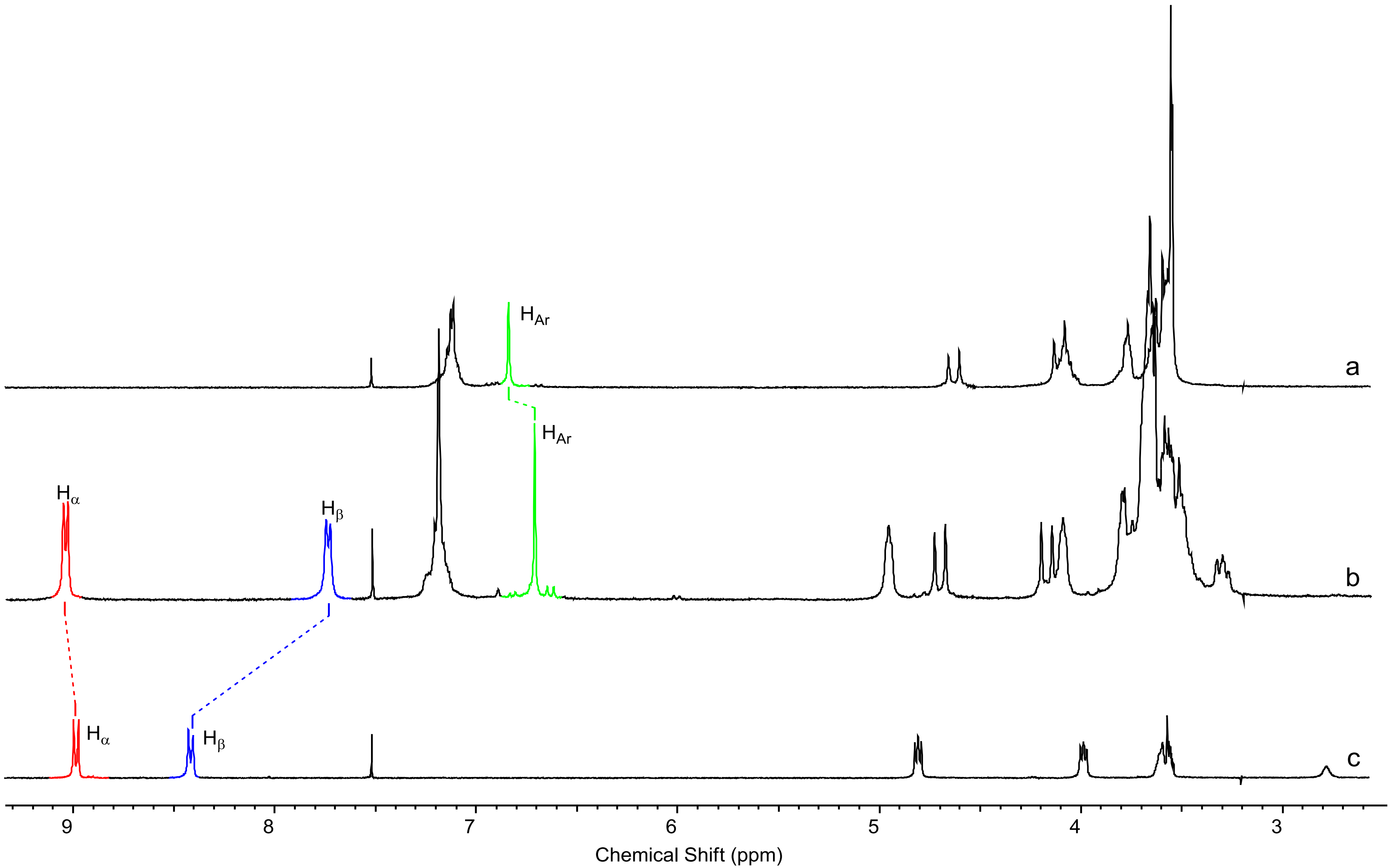

Figure S18: ^{1}H NMR spectra (300 MHz, CD_3CN:$CDCl_3$. 4:3, v/v) of (a) free host **5**, (b) mixture of **5** and **9** (1:1), (c) free guest **9**

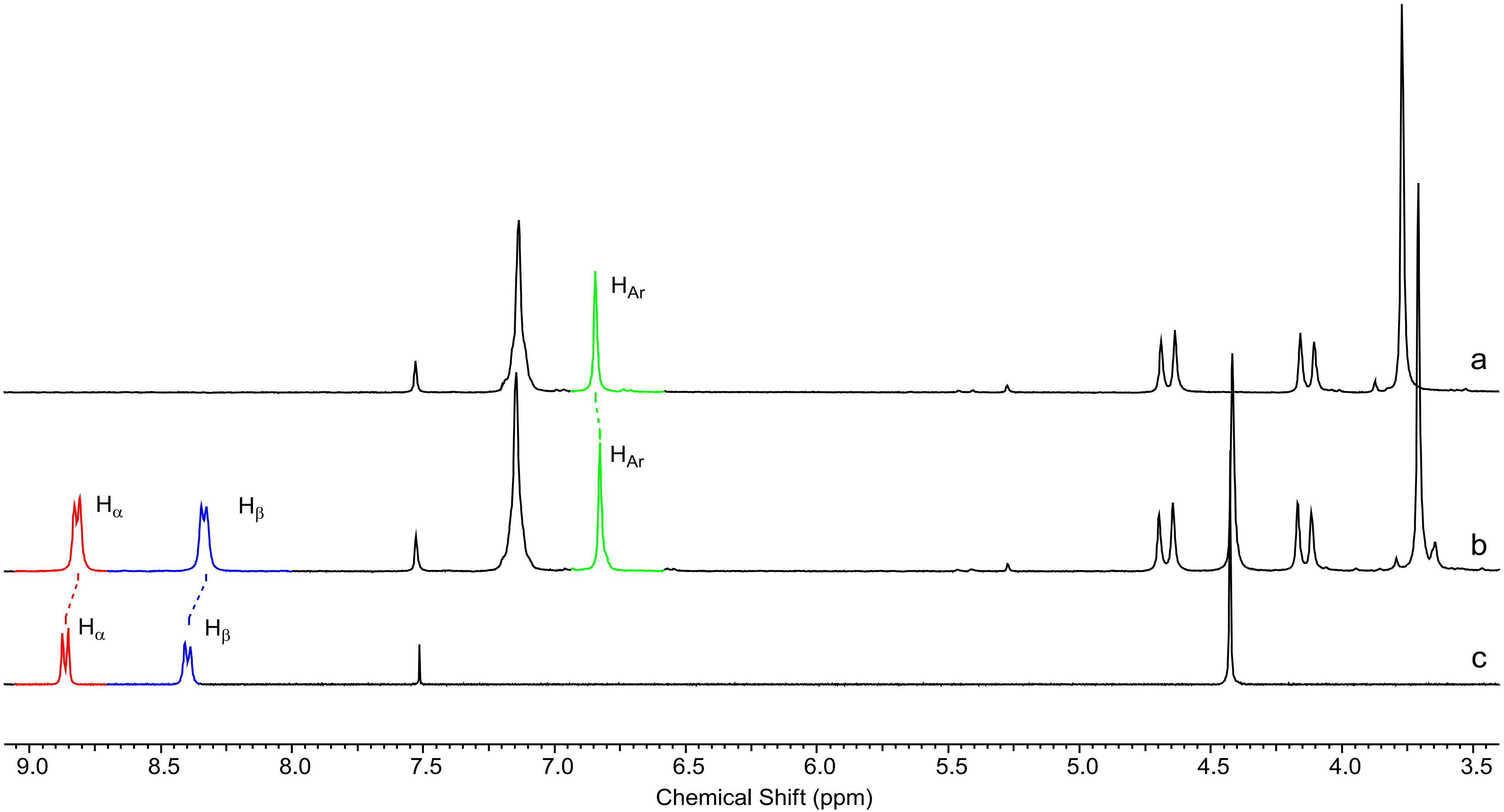

Figure S19: ^{1}H NMR spectra (300 MHz, CD_3CN:$CDCl_3$. 4:3, v/v) of (a) free host **6**, (b) mixture of **6** and **7** (1:1), (c) free guest **7**

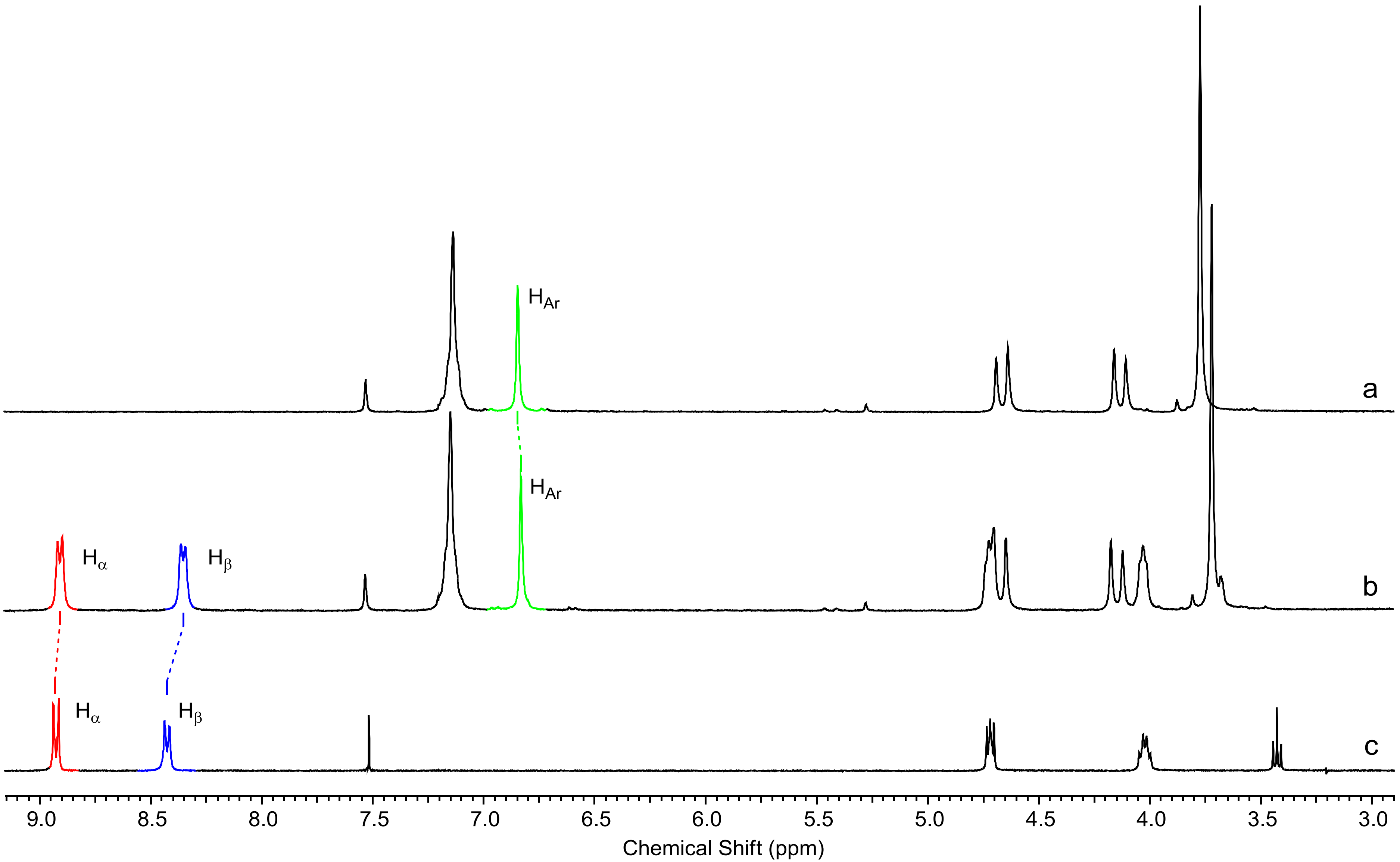

Figure S20: ^{1}H NMR spectra (300 MHz, CD_3CN:$CDCl_3$. 4:3, v/v) of (a) free host **6**, (b) mixture of **6** and **8** (1:1), (c) free guest **8**

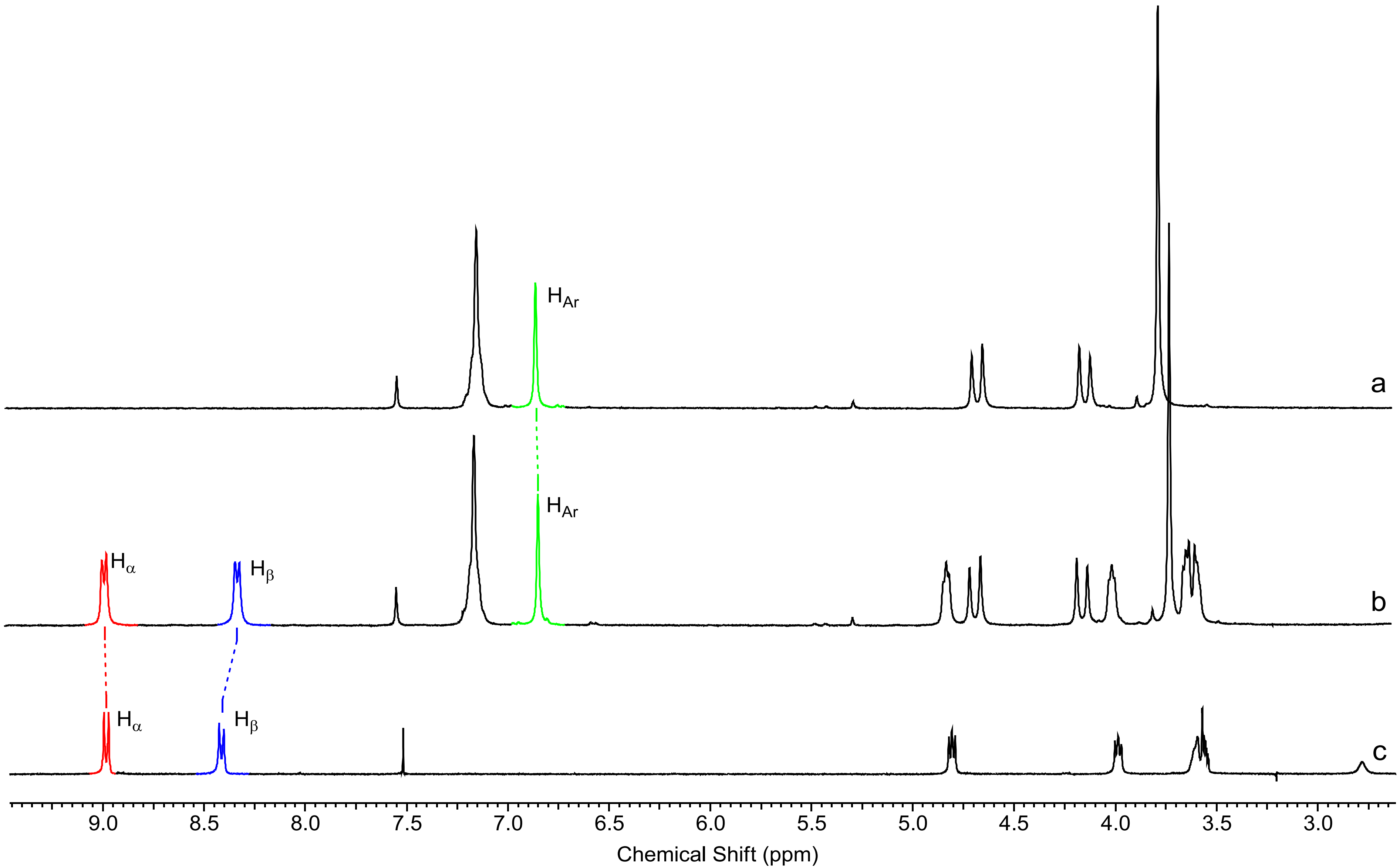

Figure S21: ^{1}H NMR spectra (300 MHz, CD_3CN:$CDCl_3$. 4:3, v/v) of(a) free host **6**, (b) mixture of **6** and **9** (1:1), (c) free guest **9**

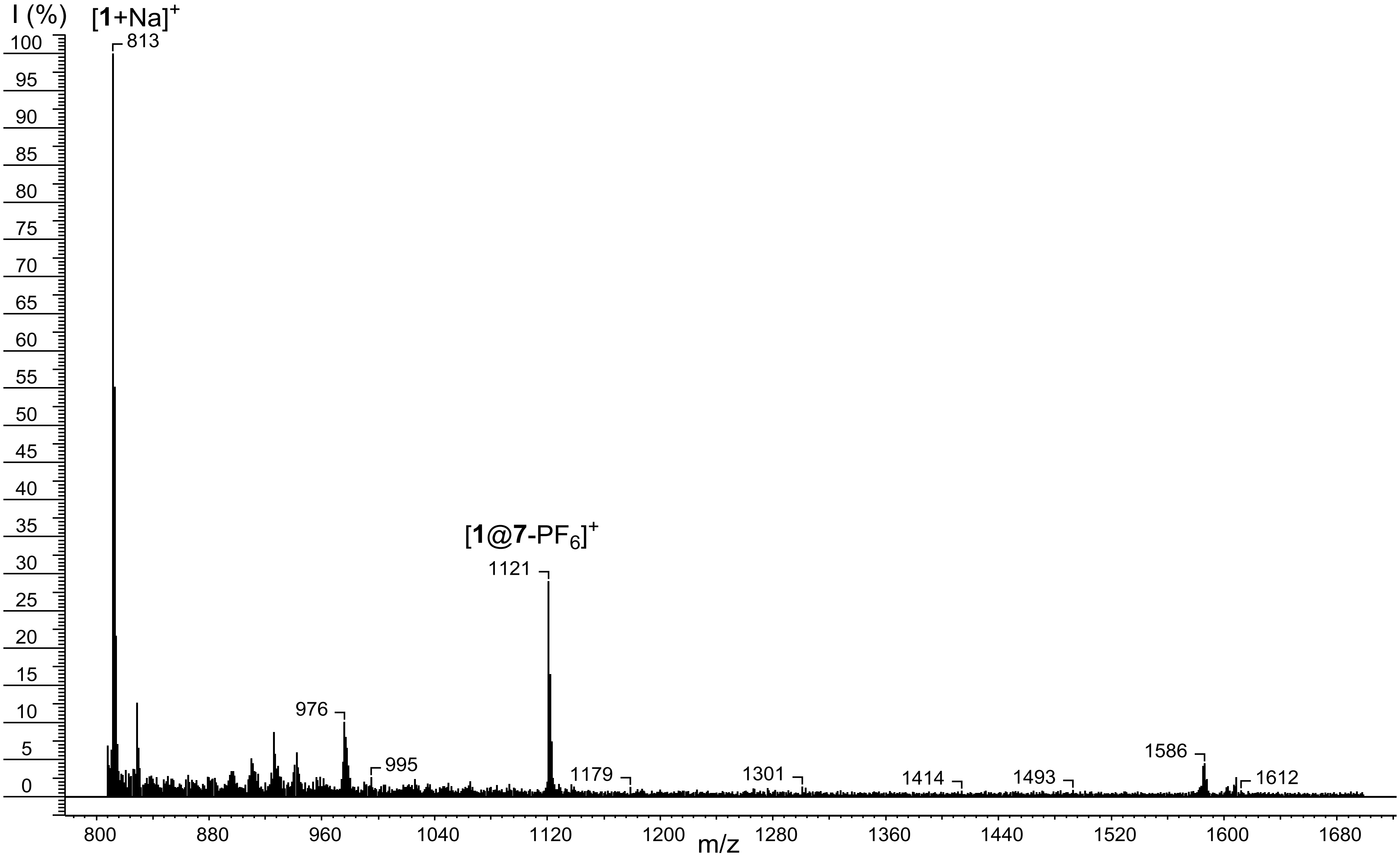

Figure S22: FAB–MS spectrum of the complex **1@7** (3-nitrobenzyl alcohol).

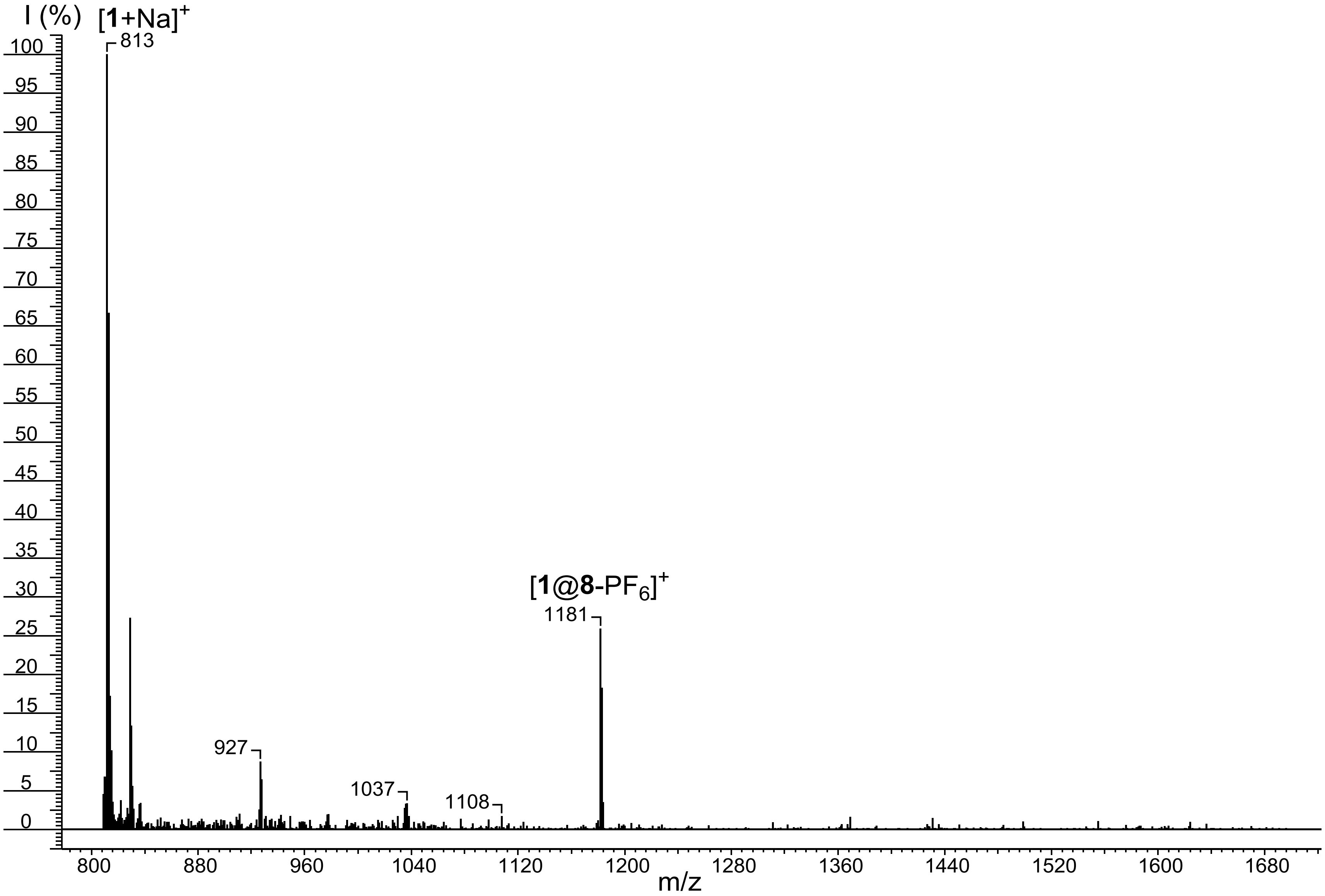

Figure S23: FAB–MS spectrum of the complex **1@8** (3-nitrobenzyl alcohol).

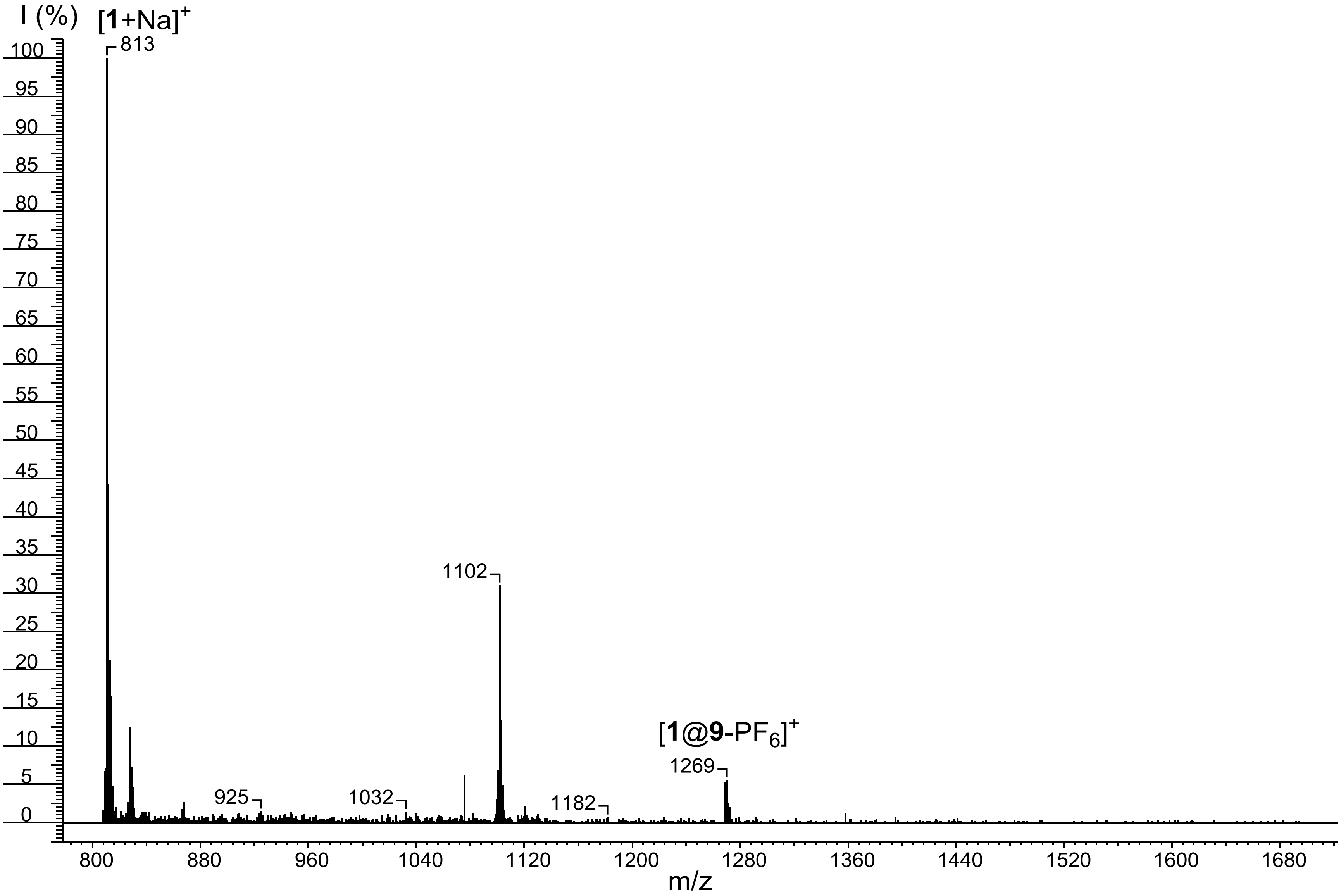

Figure S24: FAB–MS spectrum of the complex **1@9** (3-nitrobenzyl alcohol).

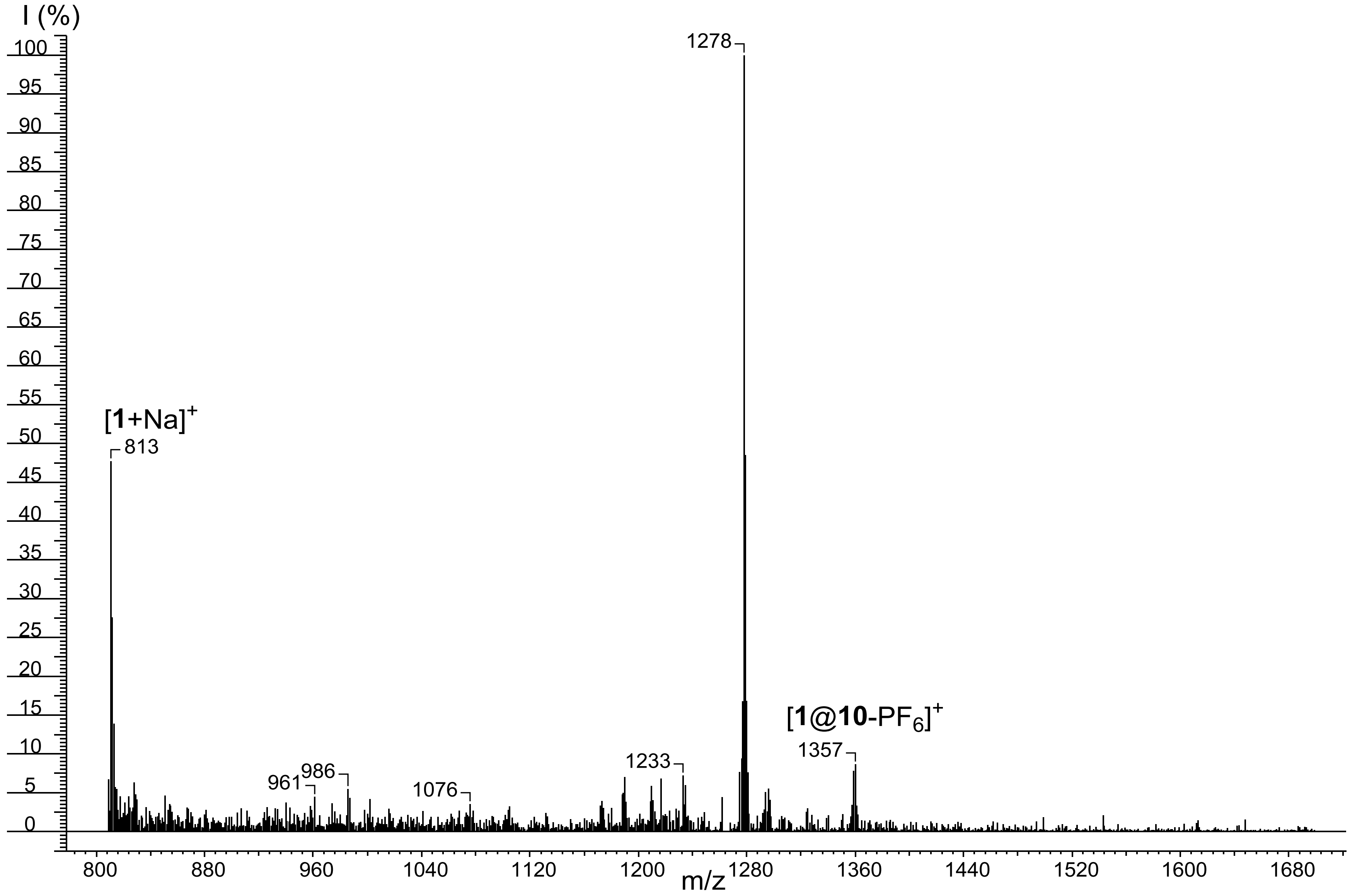

Figure S25: FAB–MS spectrum of the complex **1@10** (3-nitrobenzyl alcohol).

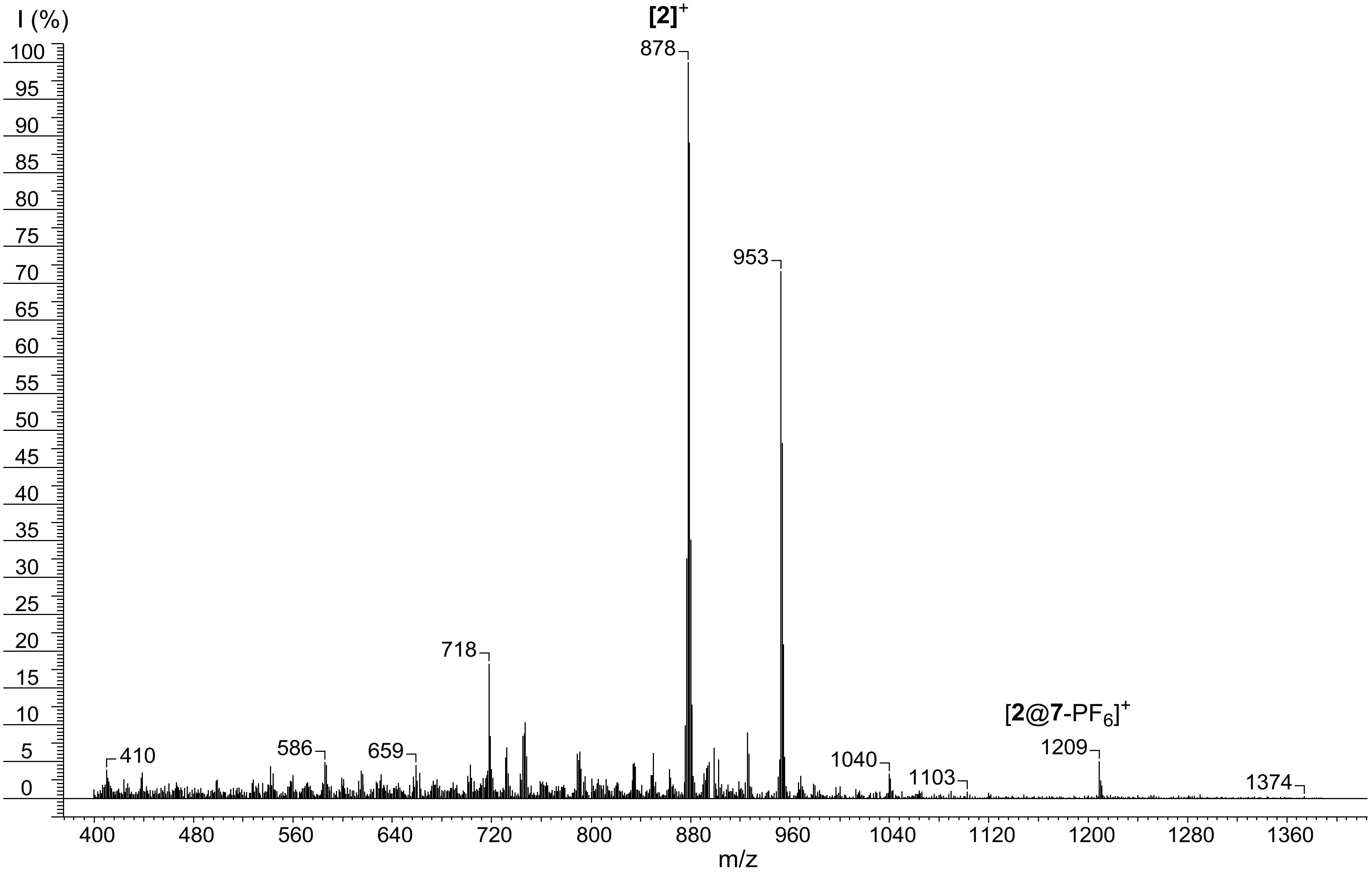

Figure S26: FAB–MS spectrum of the complex **2@7** (3-nitrobenzyl alcohol).

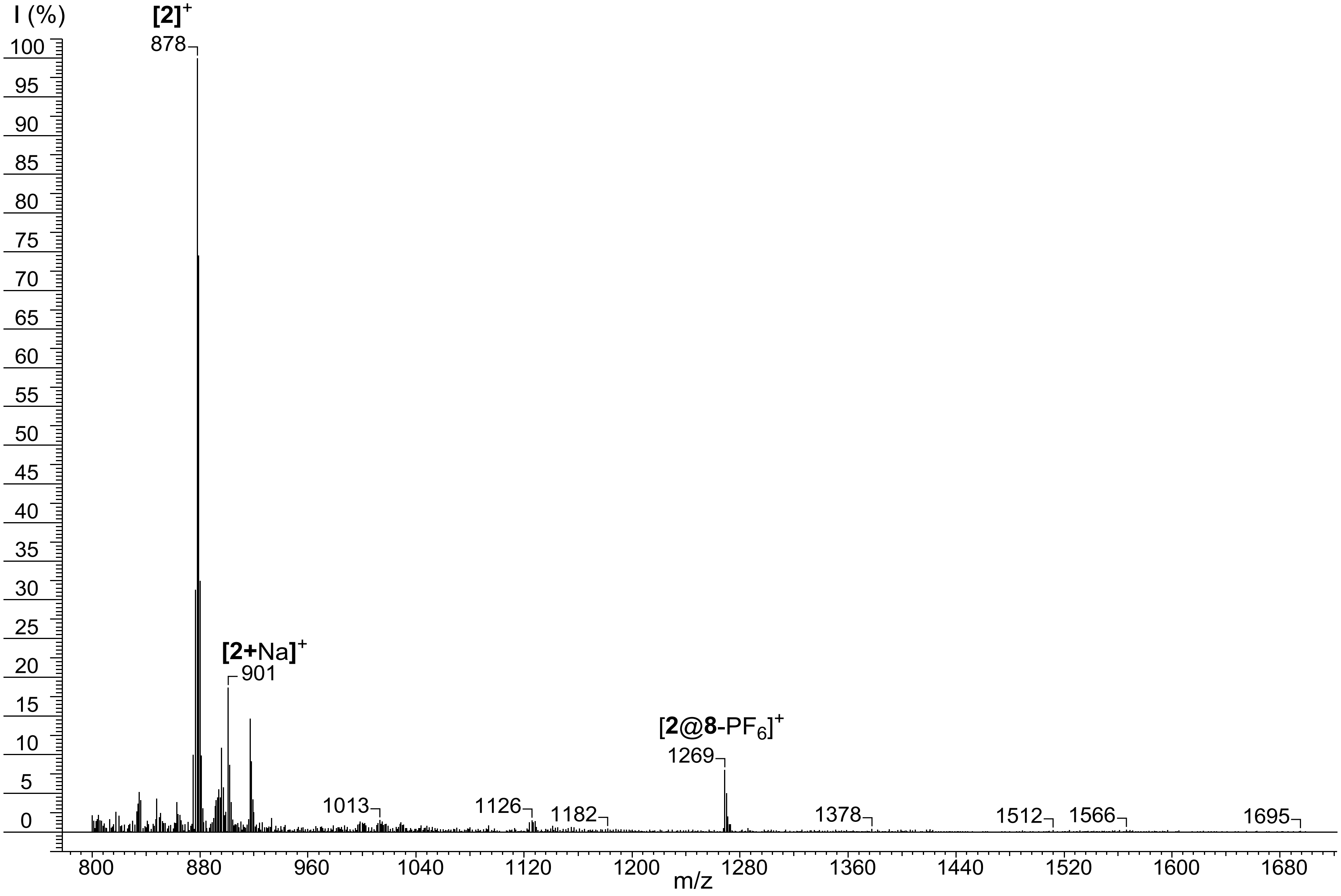

Figure S27: FAB–MS spectrum of the complex **2@8** (3-nitrobenzyl alcohol).

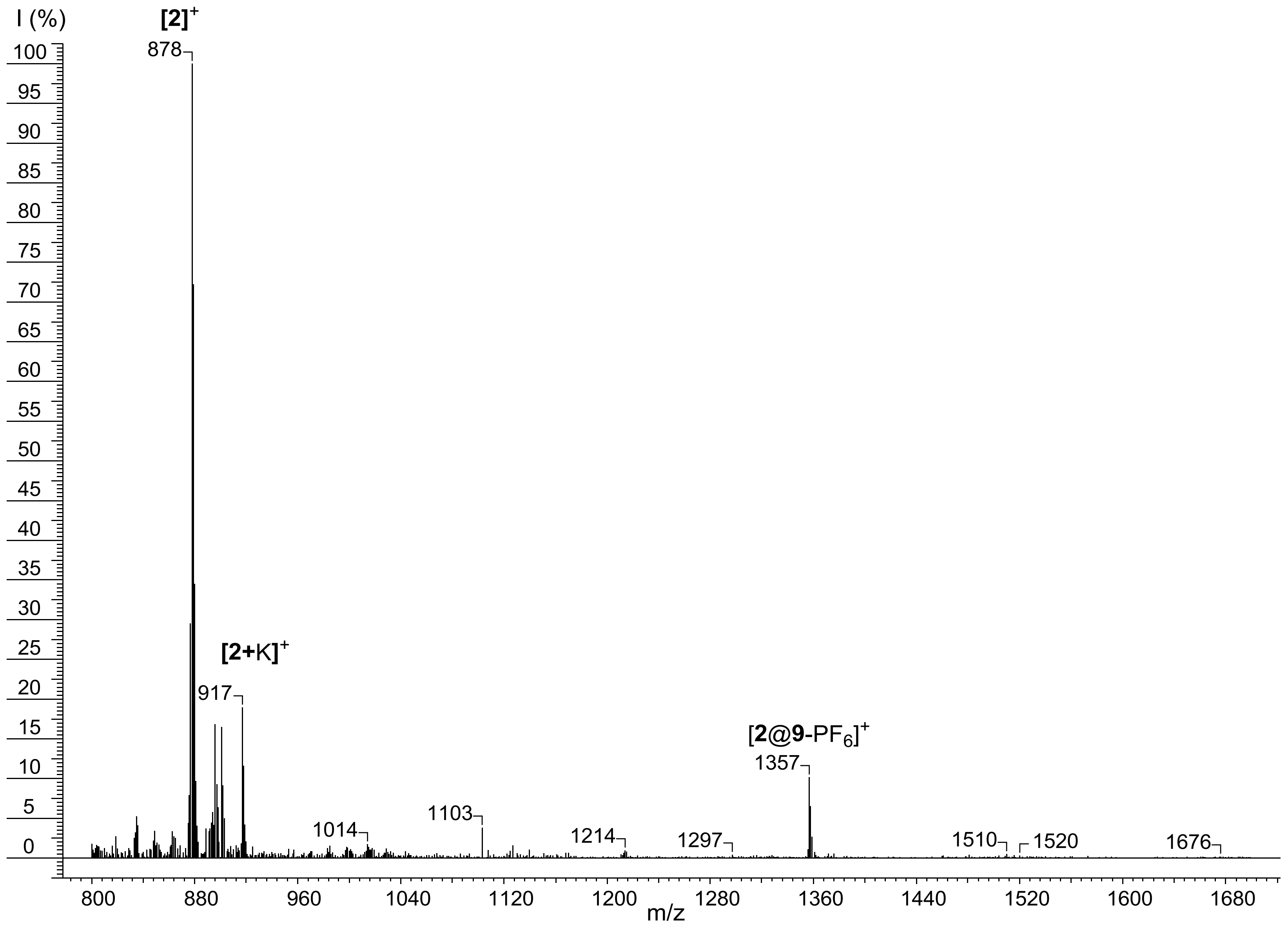

Figure S28: FAB–MS spectrum of the complex **2@9** (3-nitrobenzyl alcohol).

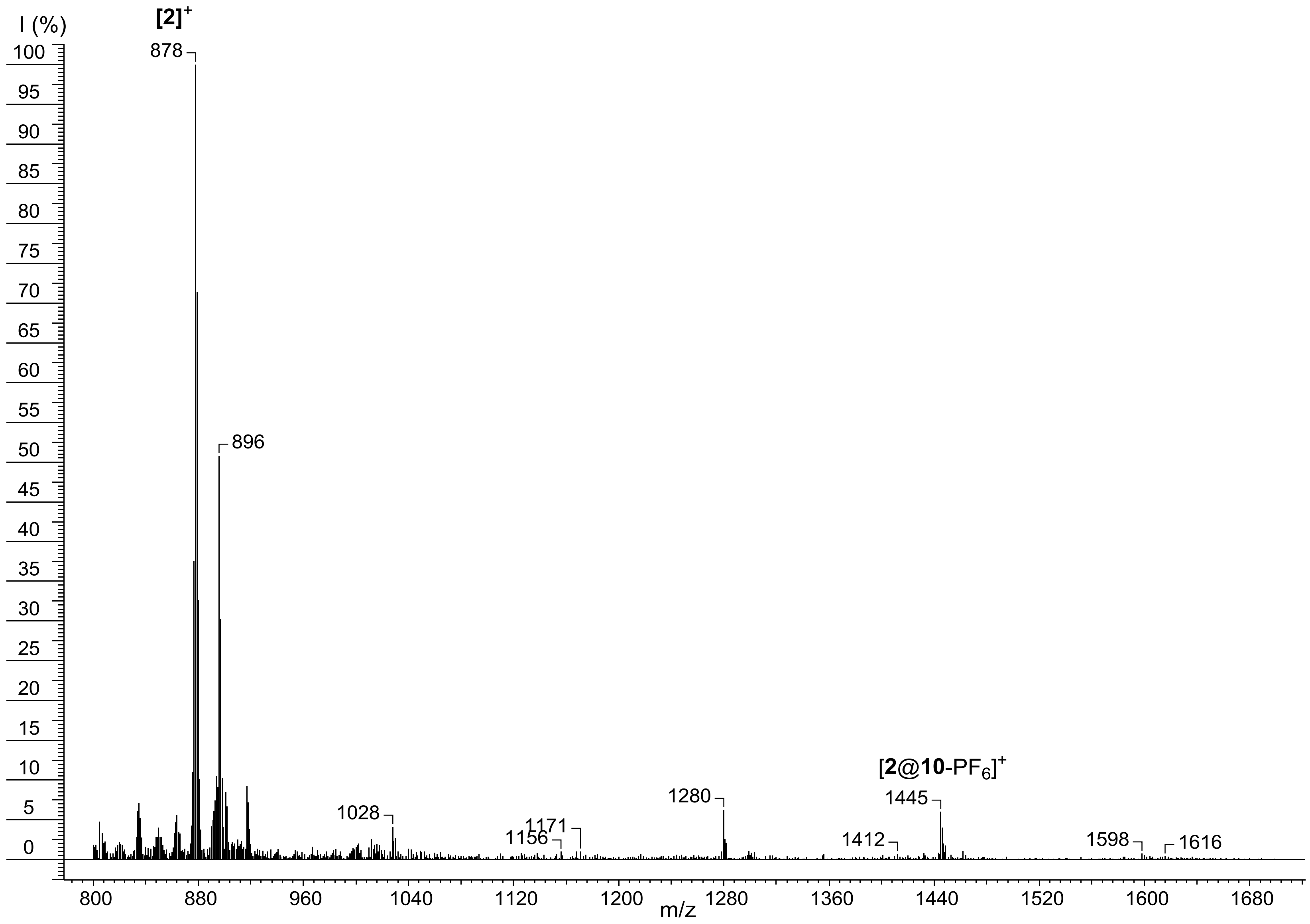

Figure S29: FAB–MS spectrum of the complex **2@10** (3-nitrobenzyl alcohol).

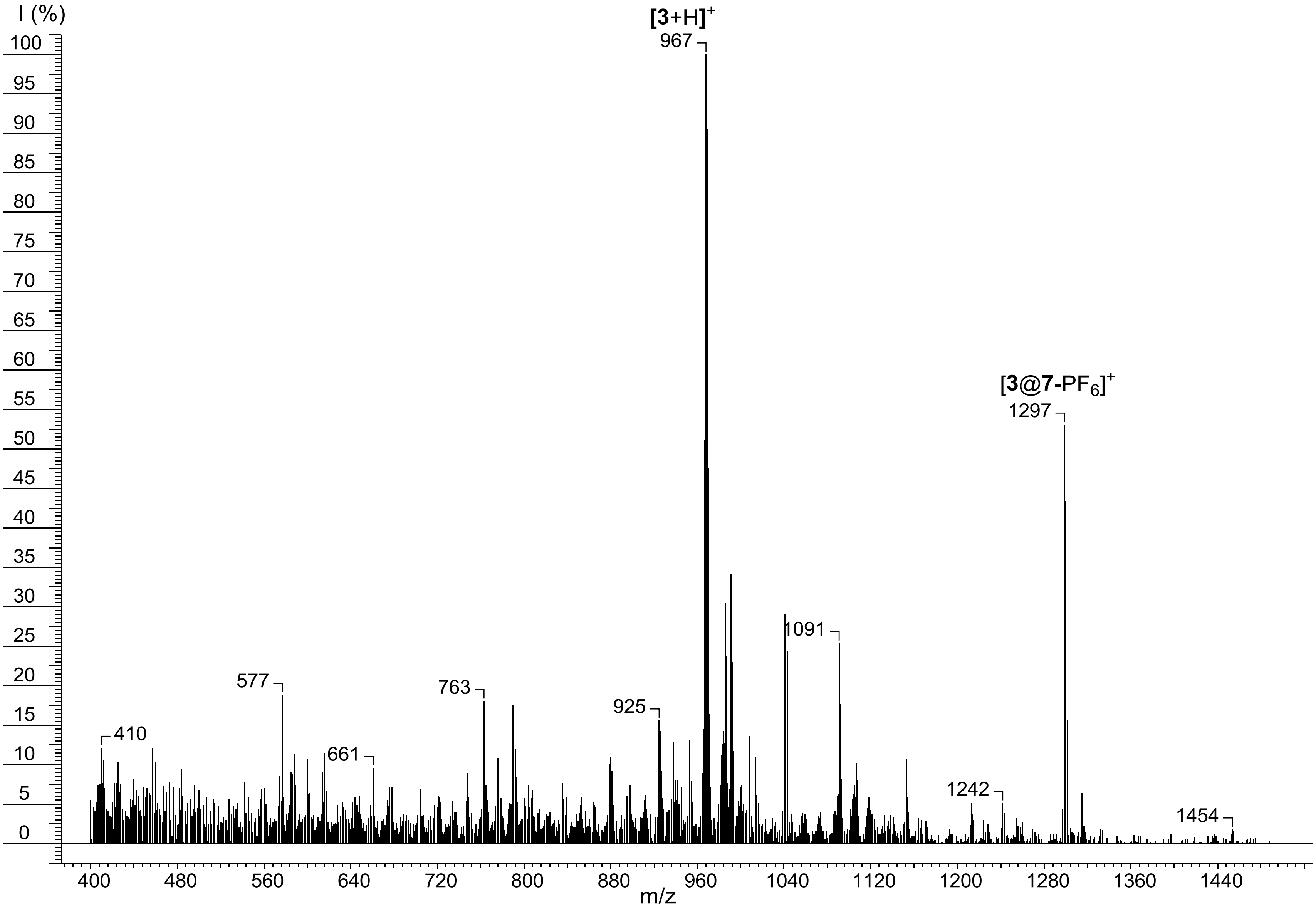

Figure S30: FAB–MS spectrum of the complex **3@7** (3-nitrobenzyl alcohol).

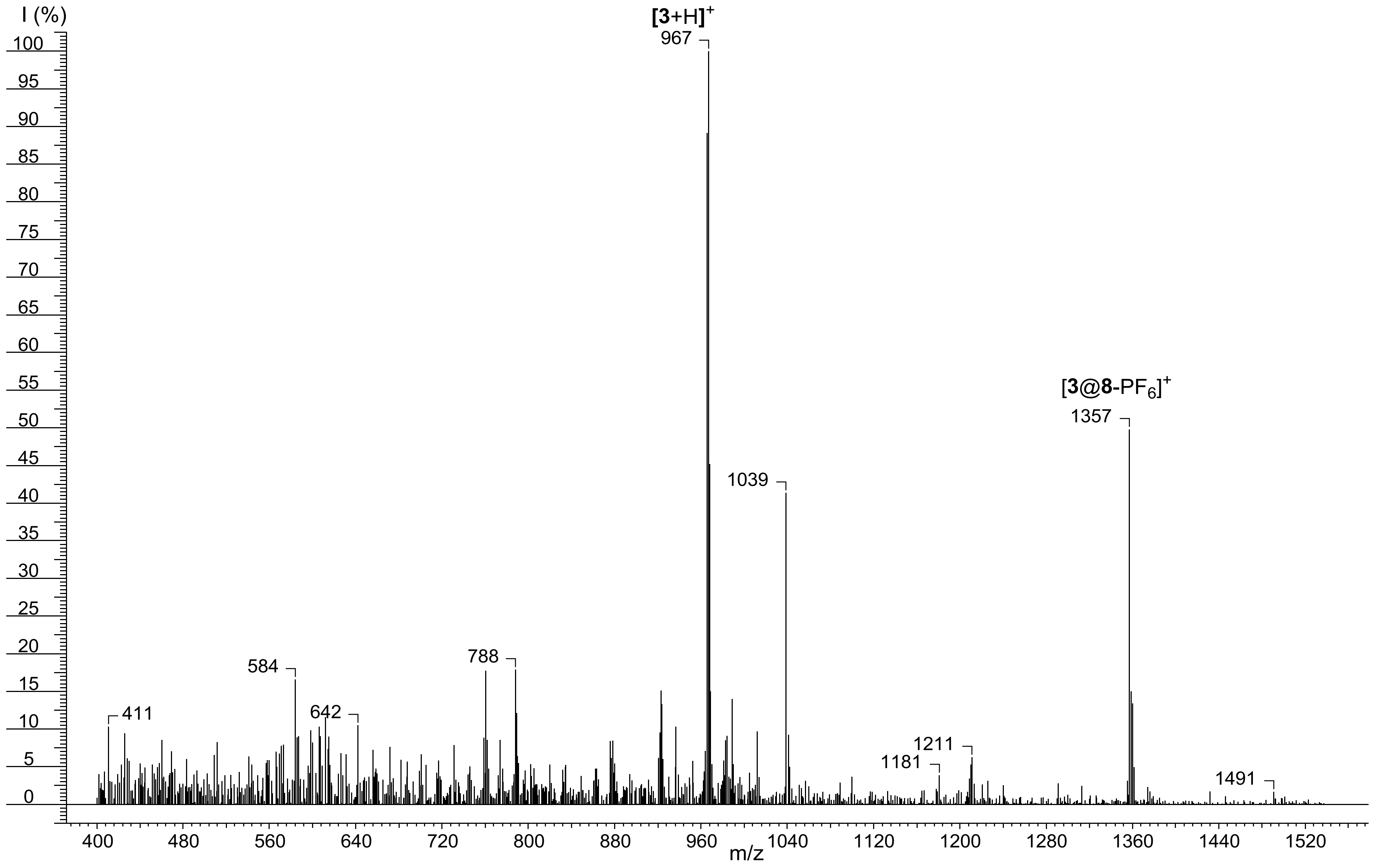

Figure S31: FAB–MS spectrum of the complex **3@8** (3-nitrobenzyl alcohol).

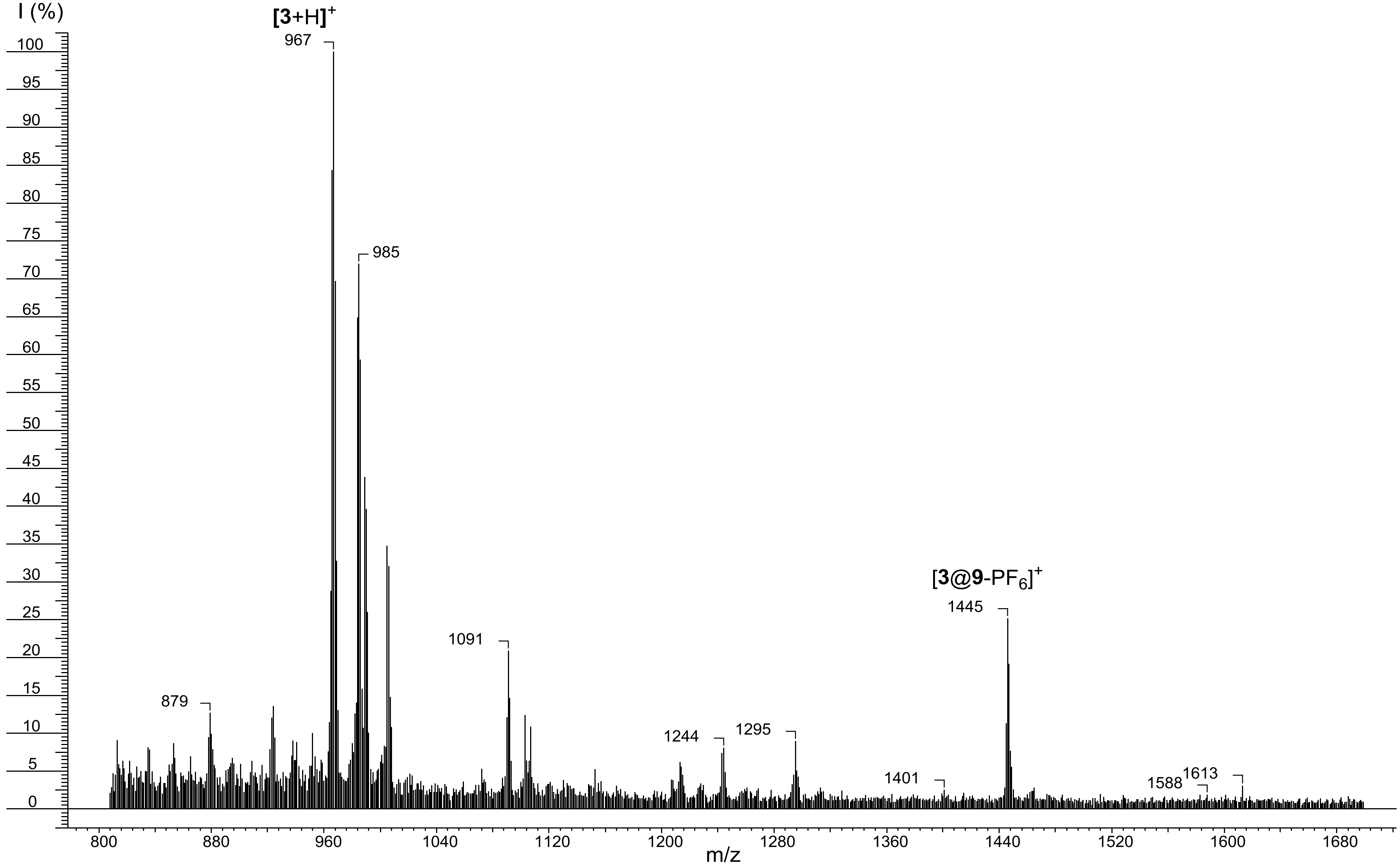

Figure S32: FAB–MS spectrum of the complex **3@9** (3-nitrobenzyl alcohol).

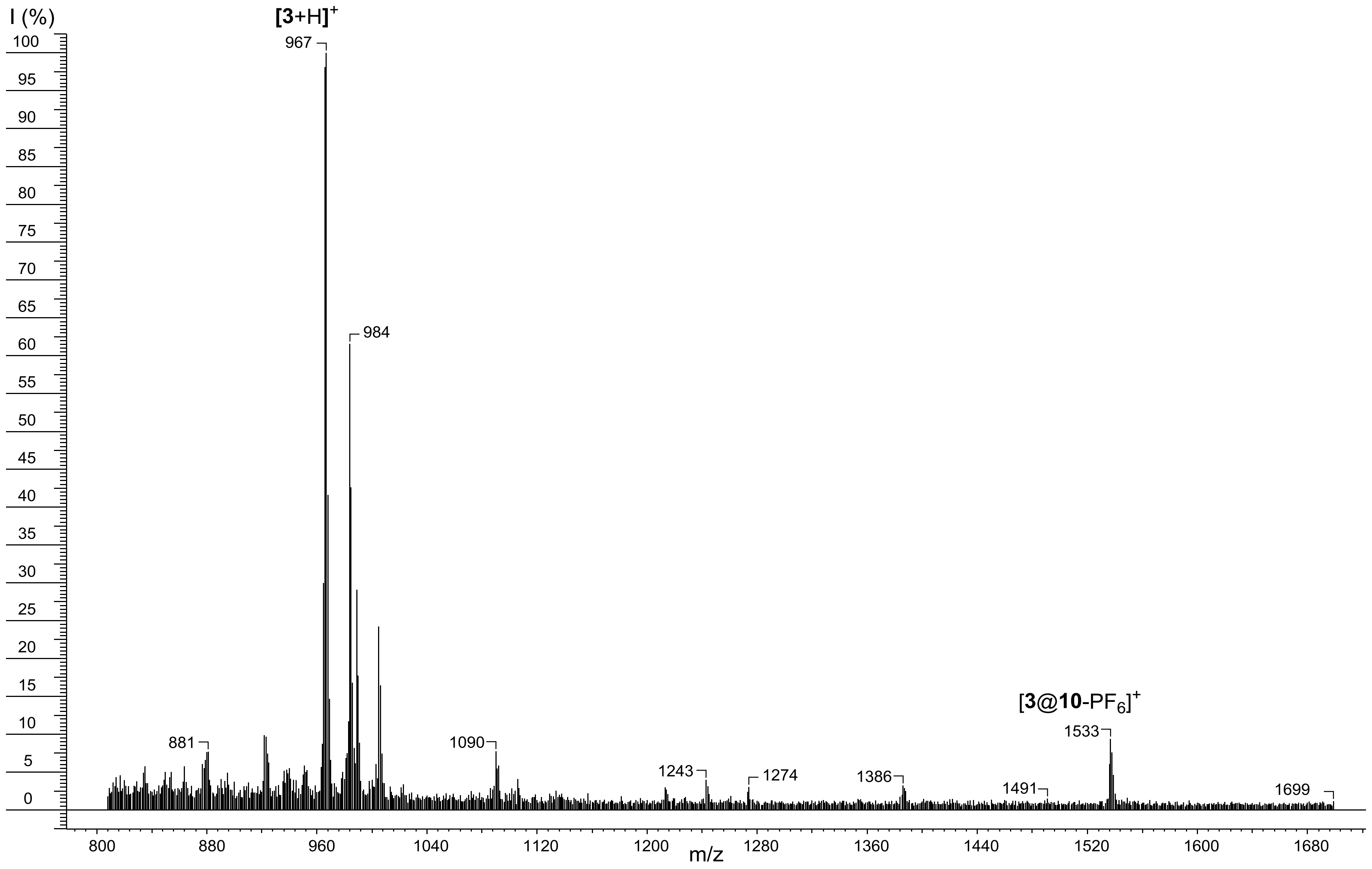

Figure S33: FAB–MS spectrum of the complex **3@10** (3-nitrobenzyl alcohol).

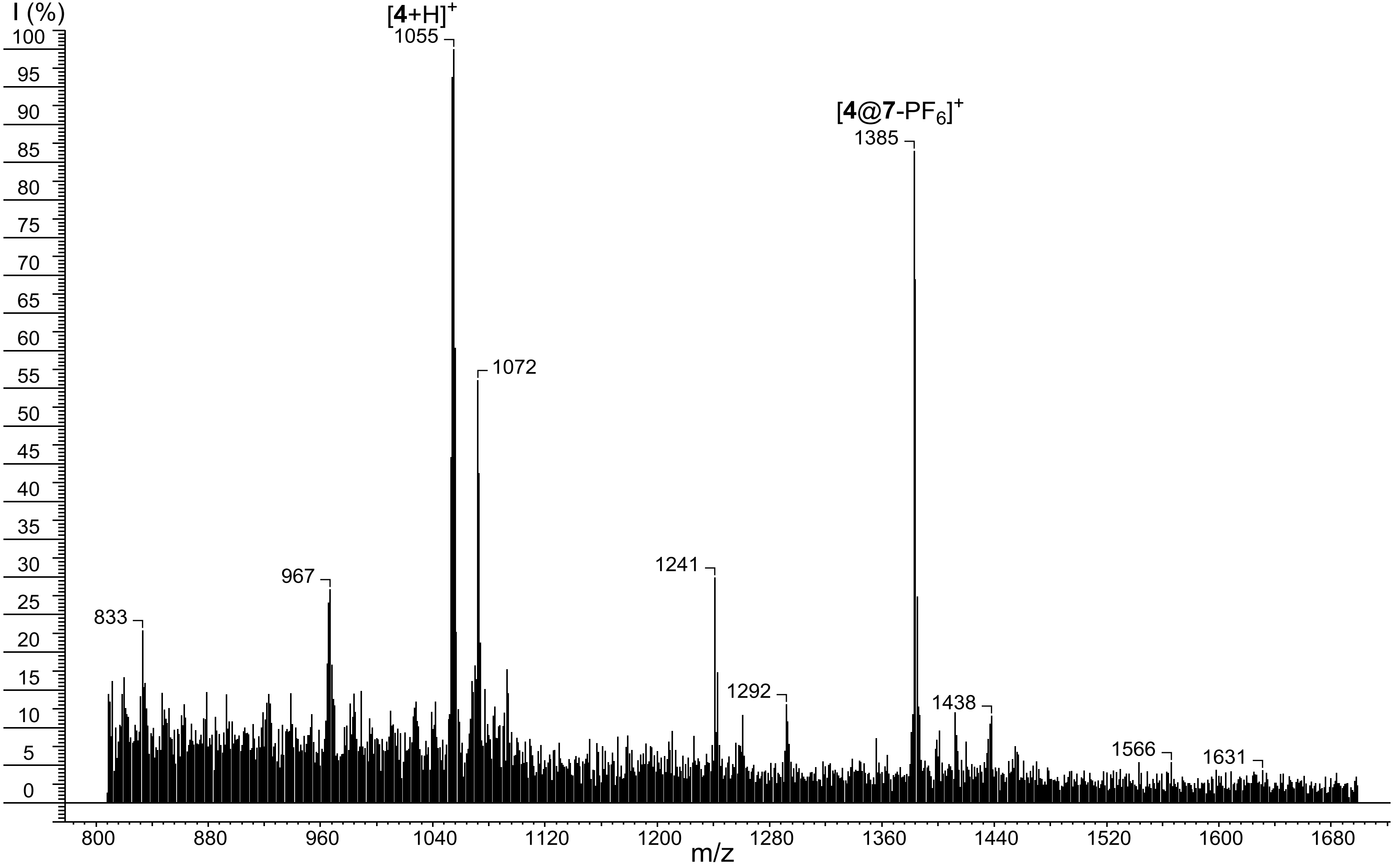

Figure S34: FAB–MS spectrum of the complex **4@7** (3-nitrobenzyl alcohol).

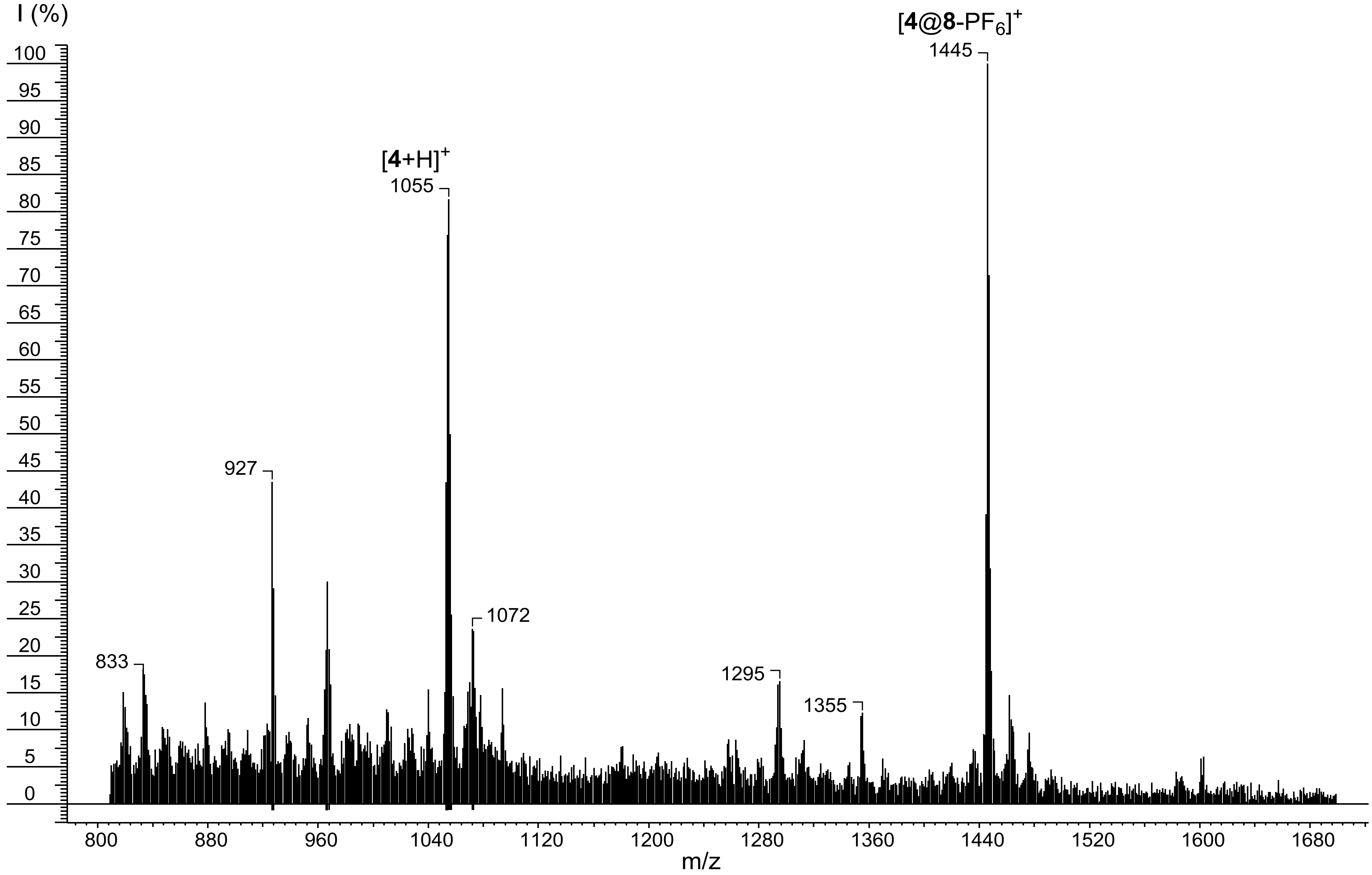

Figure S35: FAB–MS spectrum of the complex **4@8** (3-nitrobenzyl alcohol).

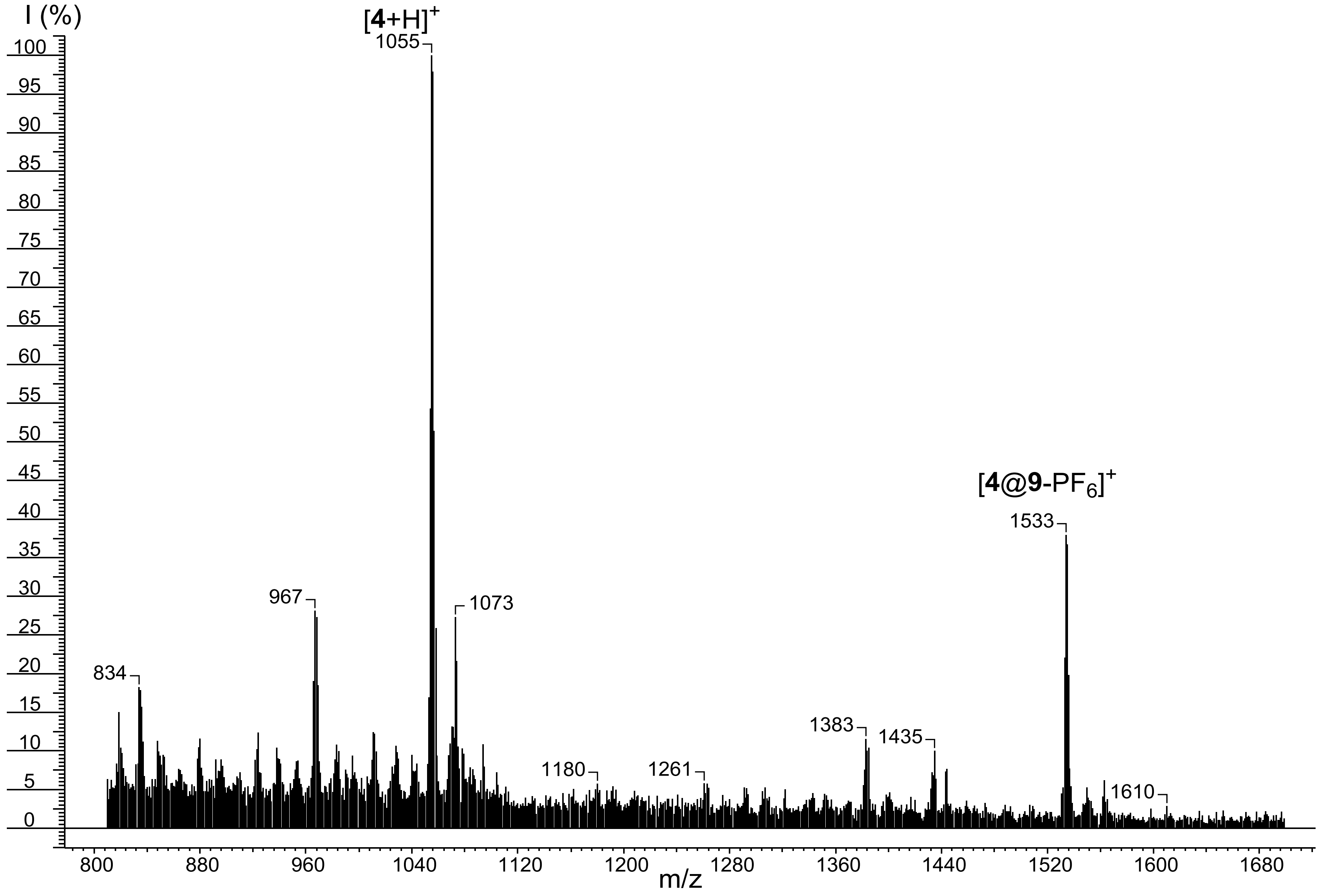

Figure S36: FAB–MS spectrum of the complex **4@9** (3-nitrobenzyl alcohol).

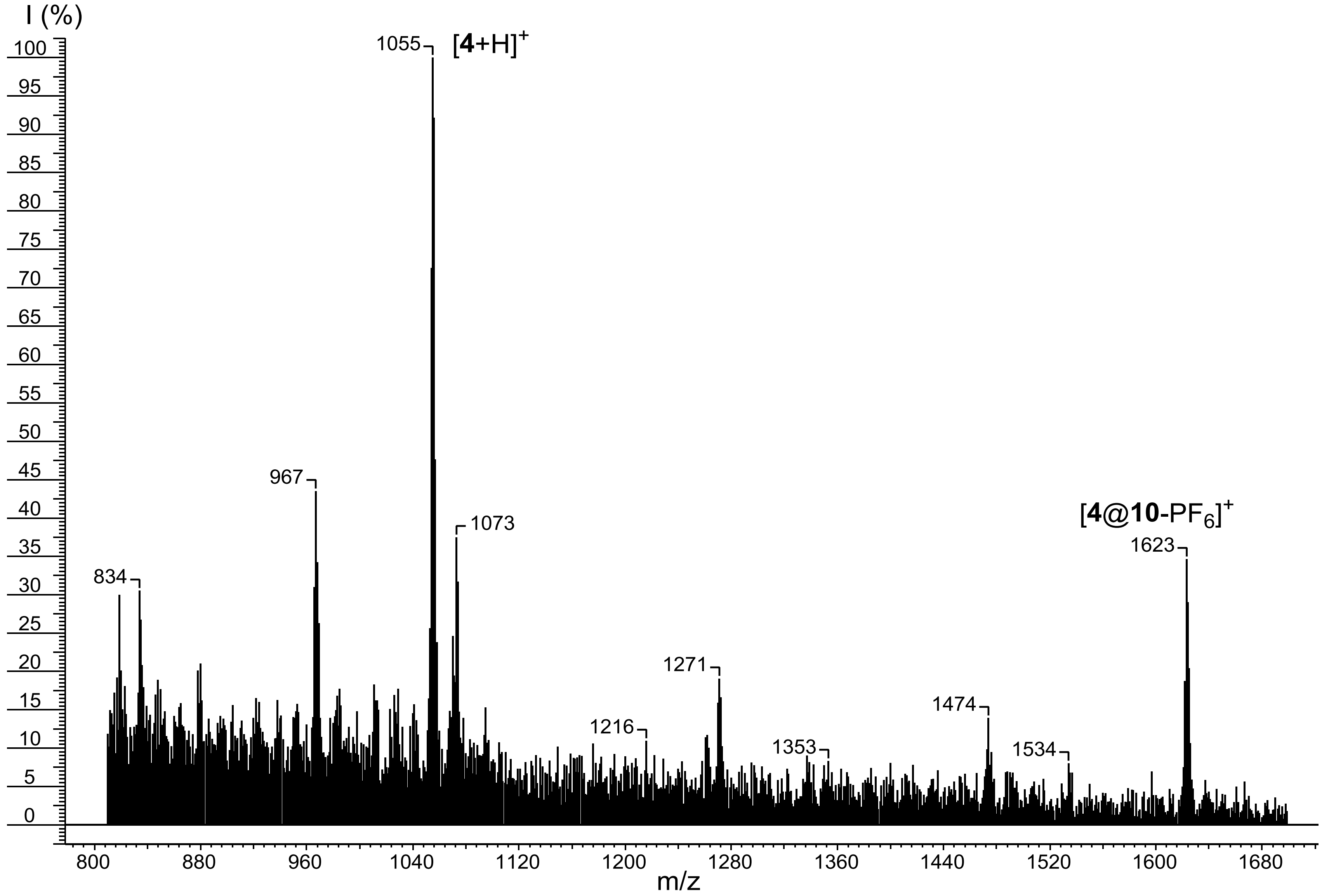

Figure S37: FAB–MS spectrum of the complex **4@10** (3-nitrobenzyl alcohol).

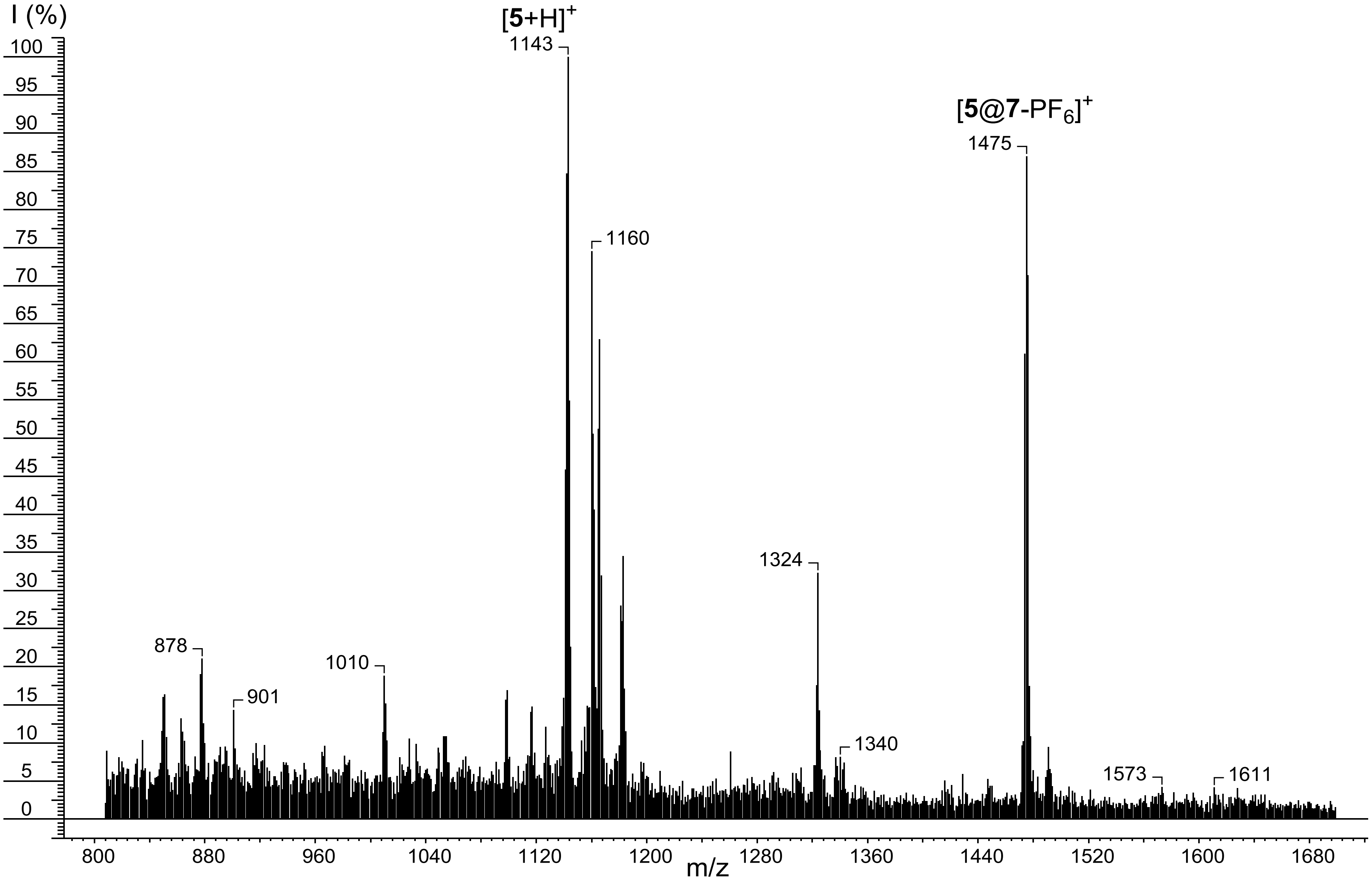

Figure S38: FAB–MS spectrum of the complex **5@7** (3-nitrobenzyl alcohol).

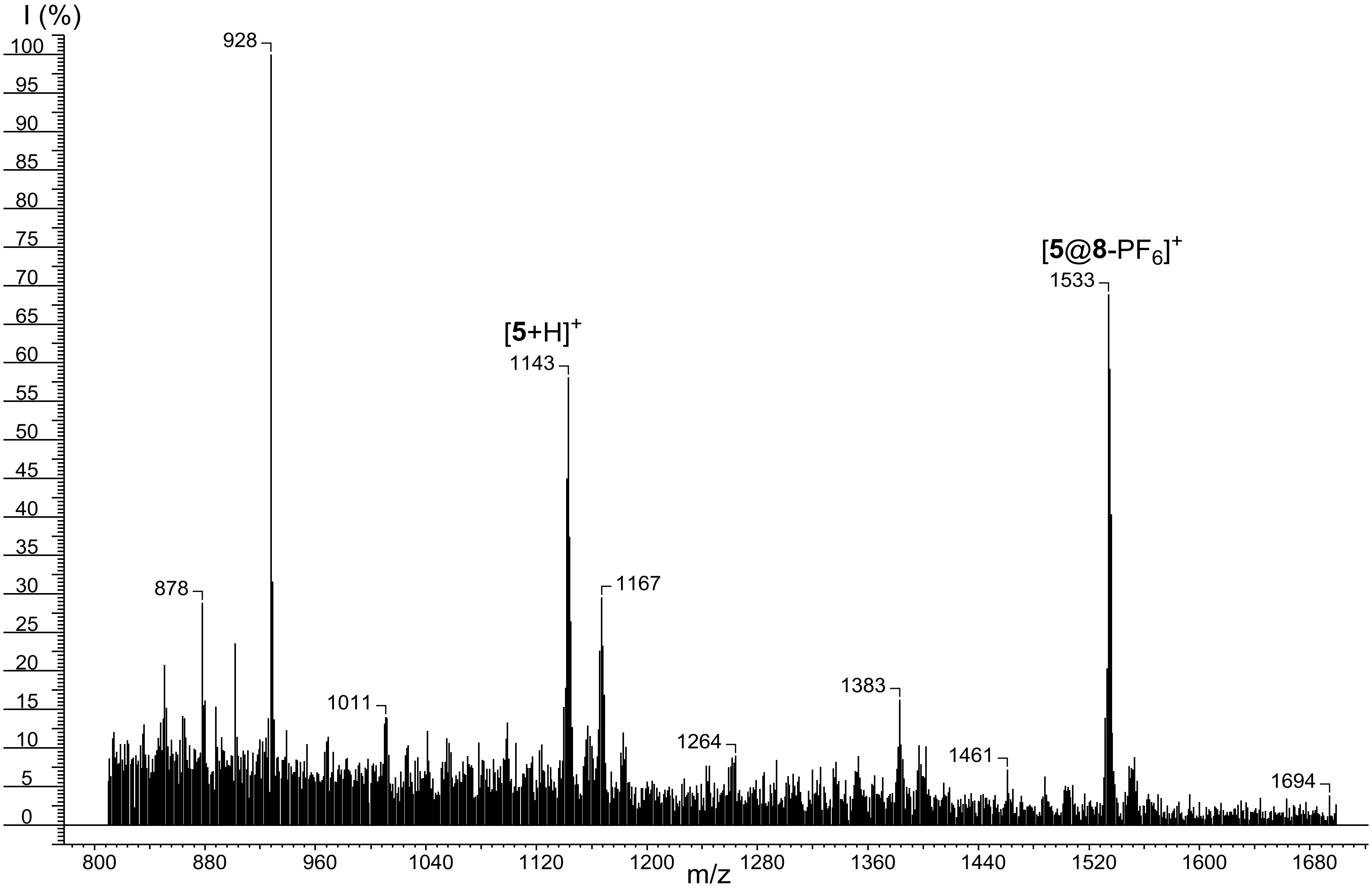

Figure S39: FAB–MS spectrum of the complex **5@8** (3-nitrobenzyl alcohol).

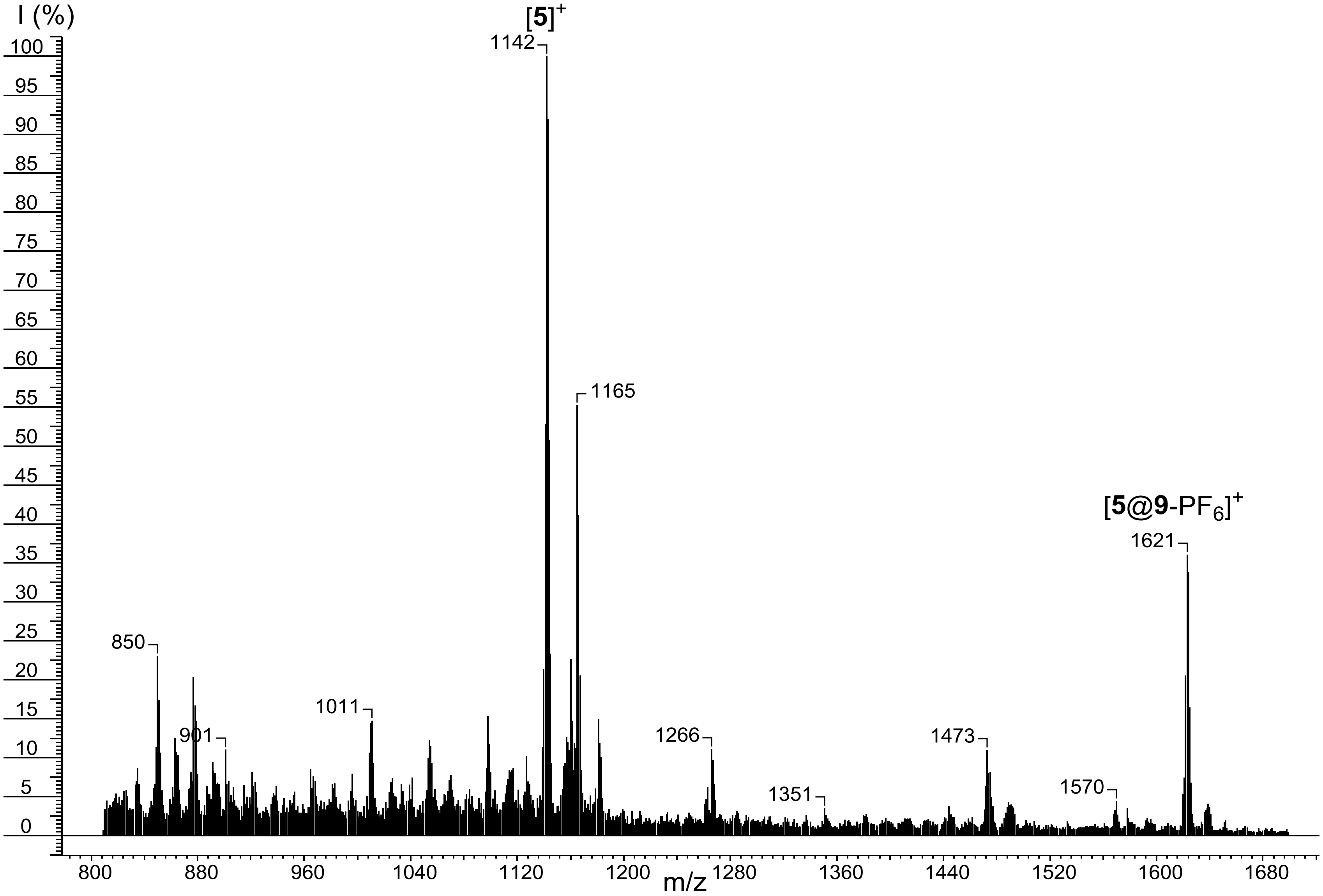

Figure S40: FAB–MS spectrum of the complex **5@9** (3-nitrobenzyl alcohol).

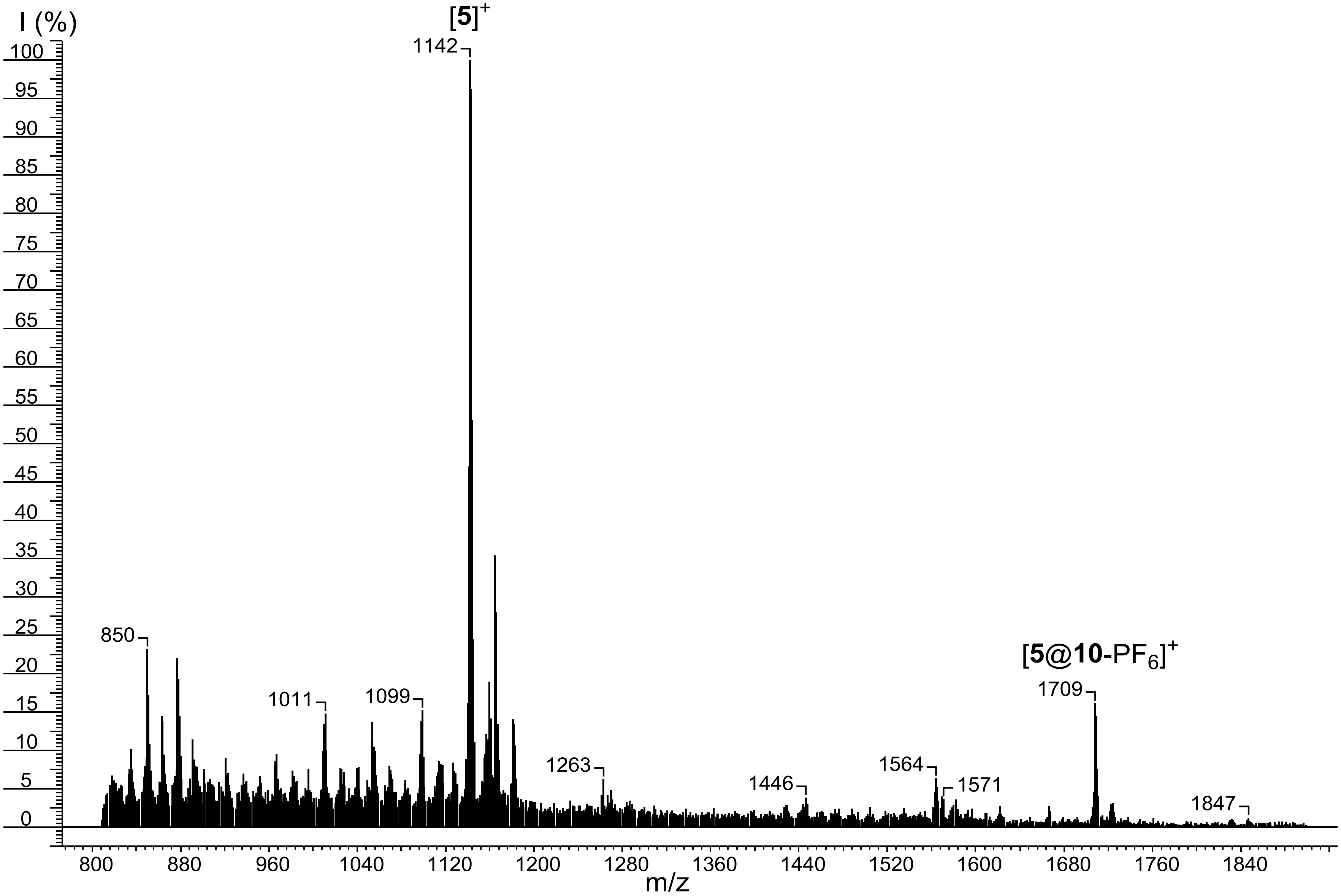

Figure S41: FAB–MS spectrum of the complex **5@10** (3-nitrobenzyl alcohol).

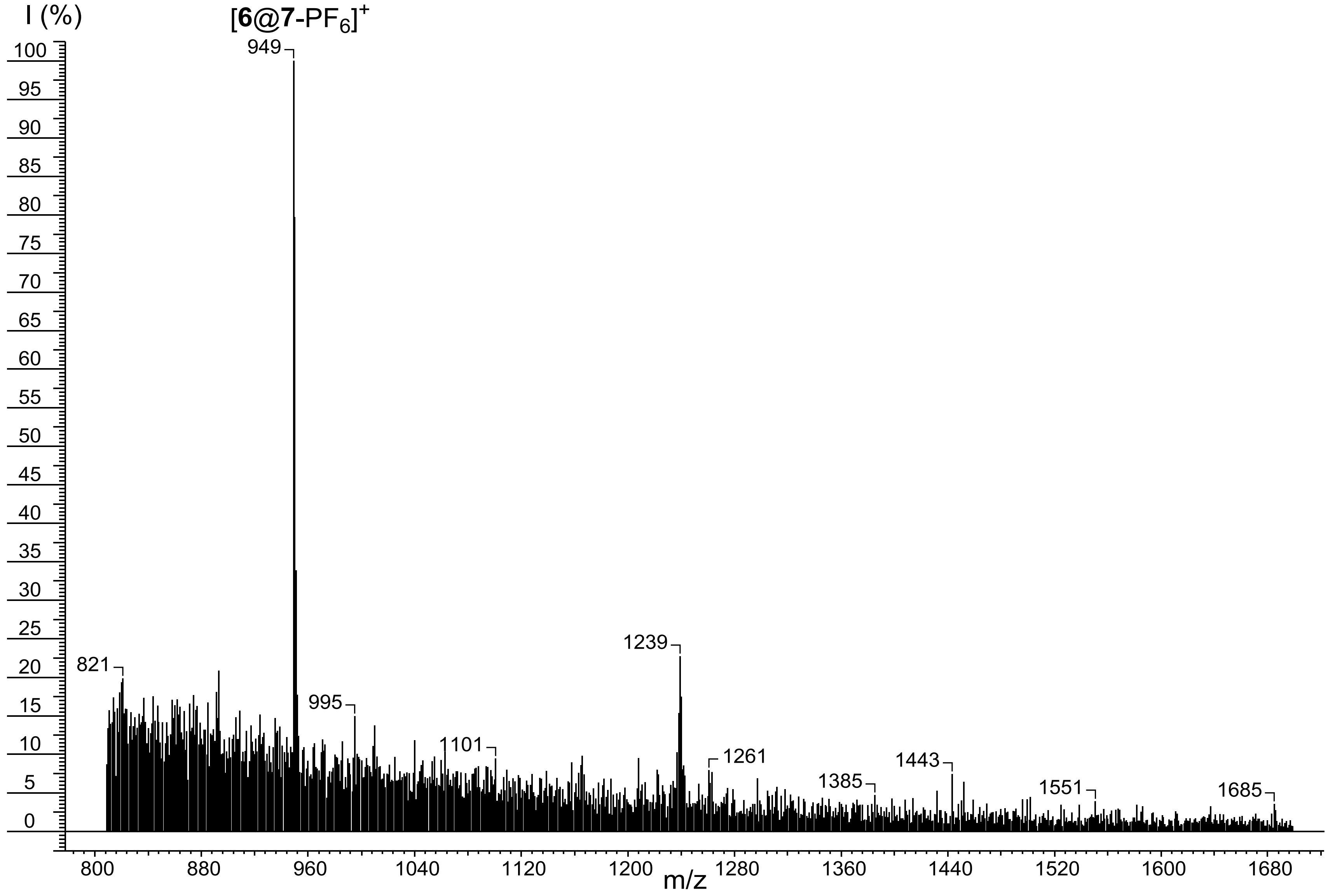

Figure S42: FAB–MS spectrum of the complex **6@7** (3-nitrobenzyl alcohol).

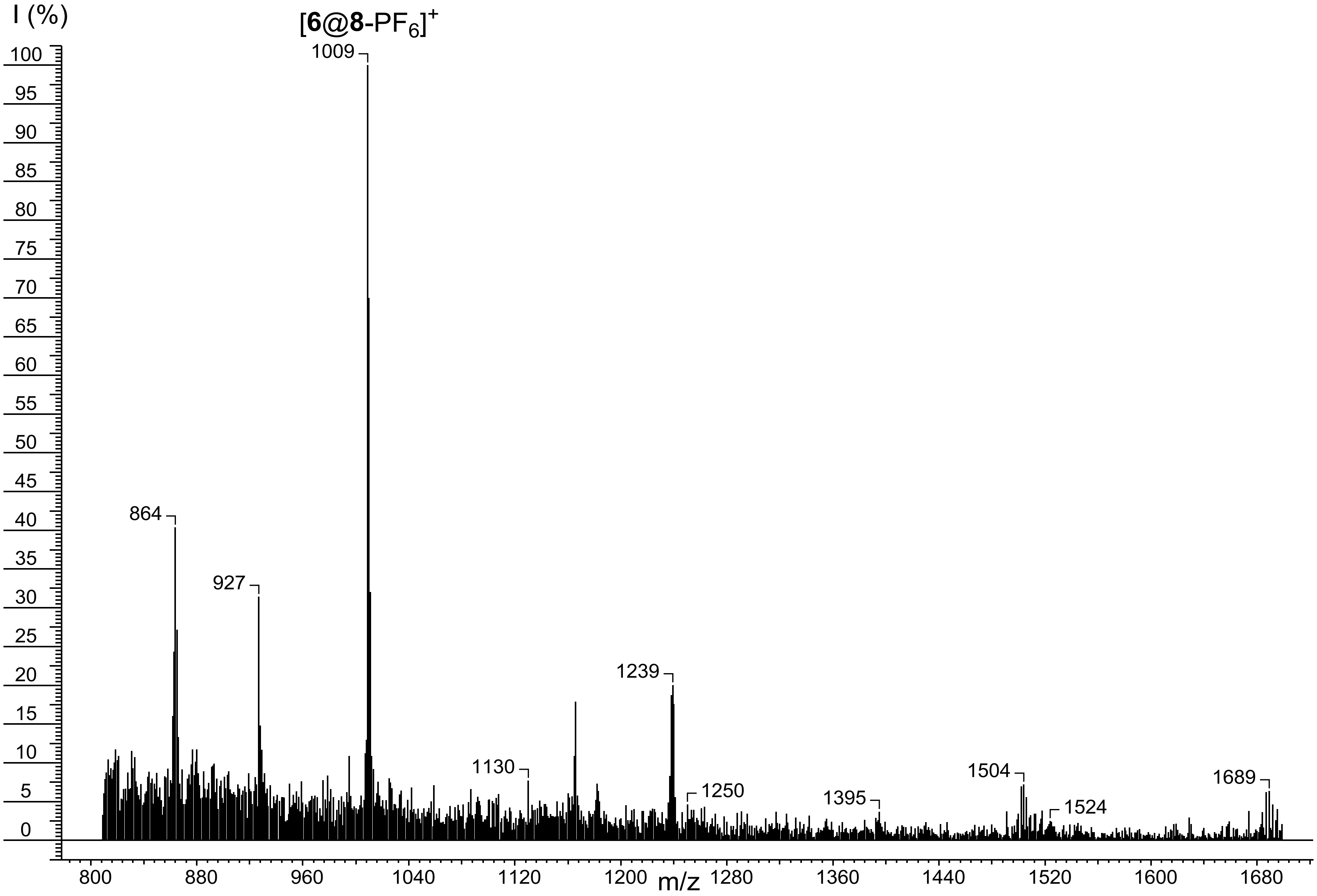

Figure S43: FAB–MS spectrum of the complex **6@8** (3-nitrobenzyl alcohol).

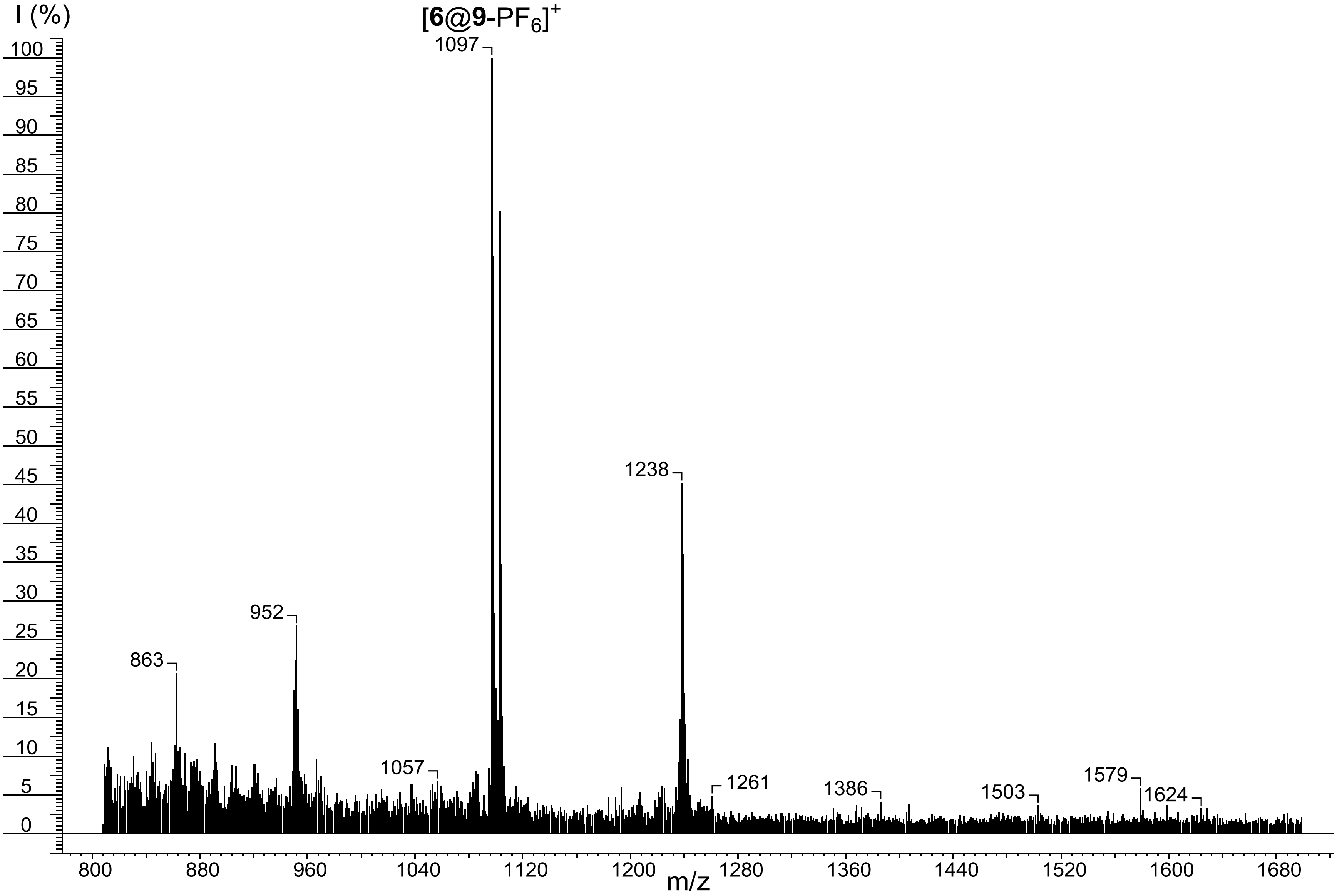

Figure S44: FAB–MS spectrum of the complex **6@9** (3-nitrobenzyl alcohol).

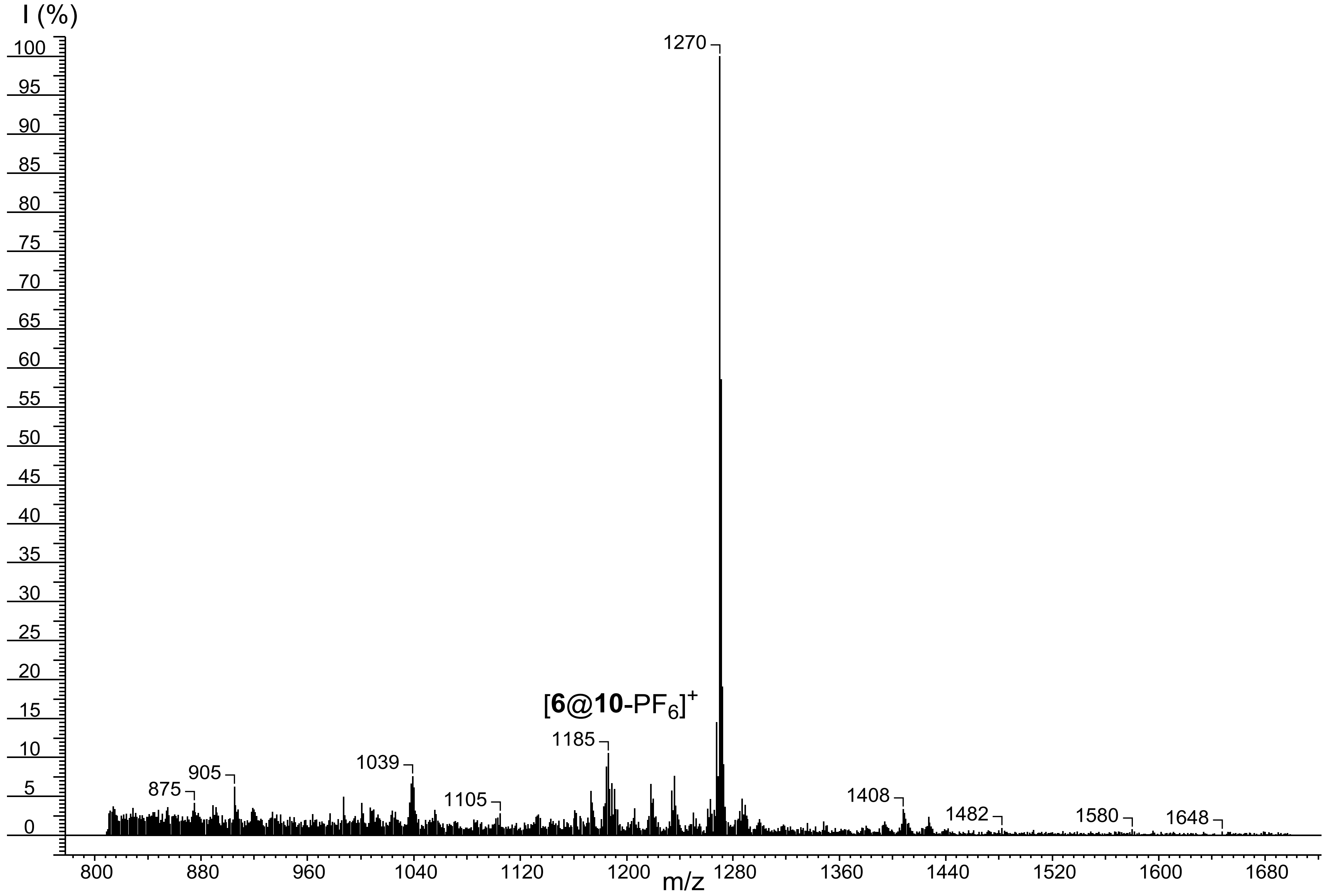

Figure S45: FAB–MS spectrum of the complex **6@10** (3-nitrobenzyl alcohol).

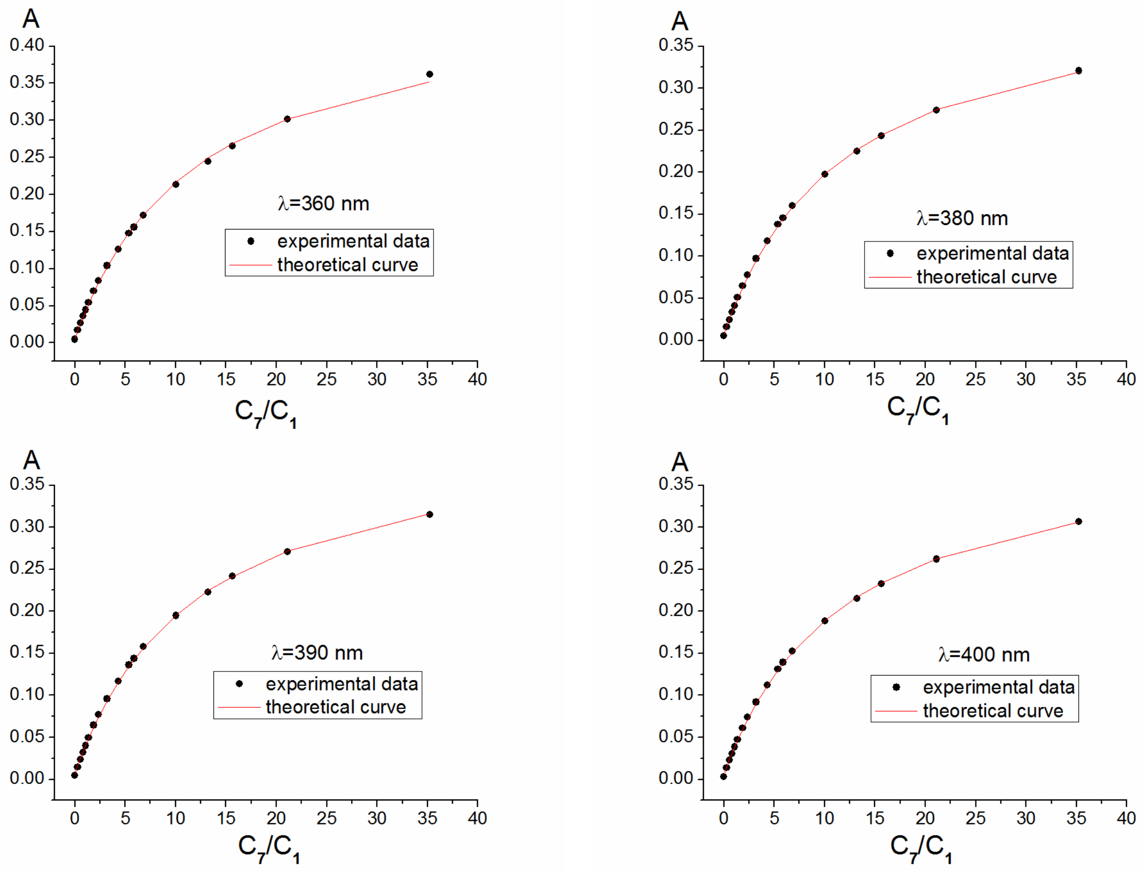

Figure S46: The binding curves at different wavelengths for the molecular clip **1** with guest **7** (C_{clip} = 7.76 × 10^{-4} M).

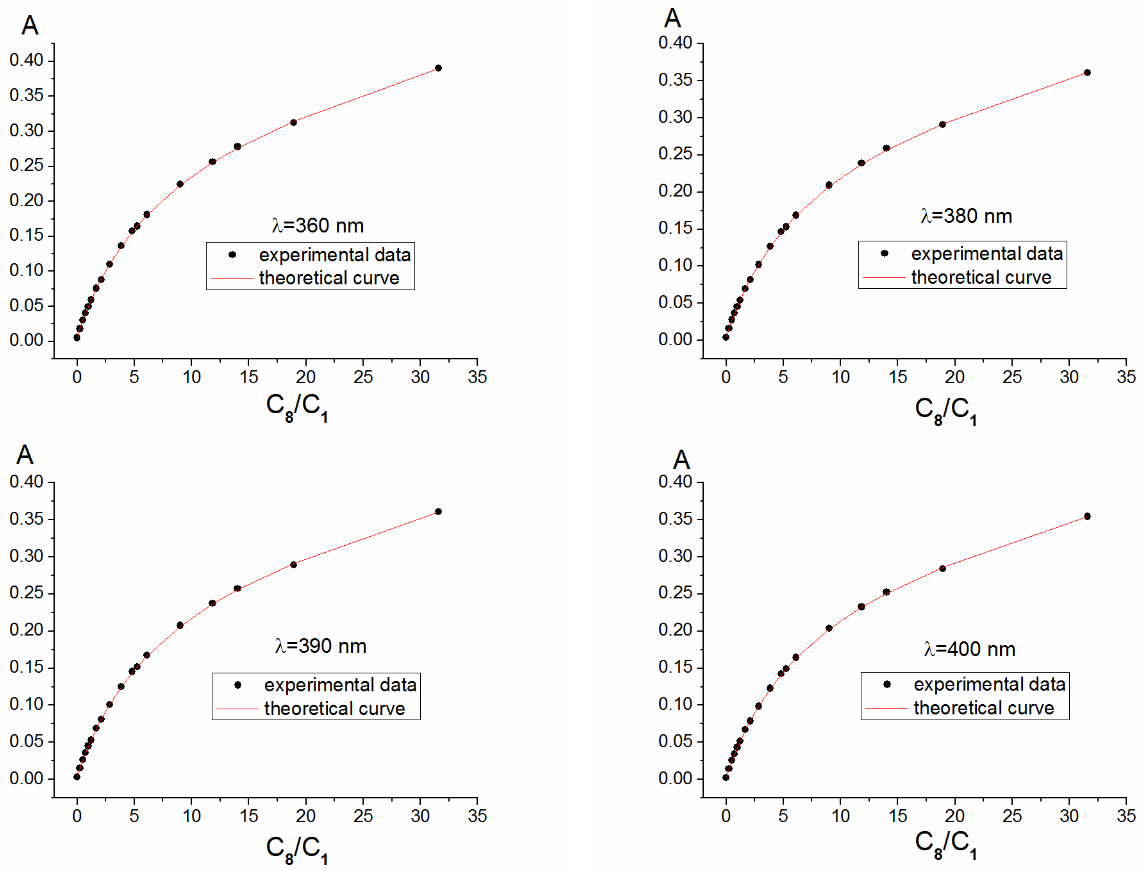

Figure S47: The binding curves at different wavelengths for the molecular clip **1** with guest **8** (C_{clip} = 8.27 × 10^{-4} M)

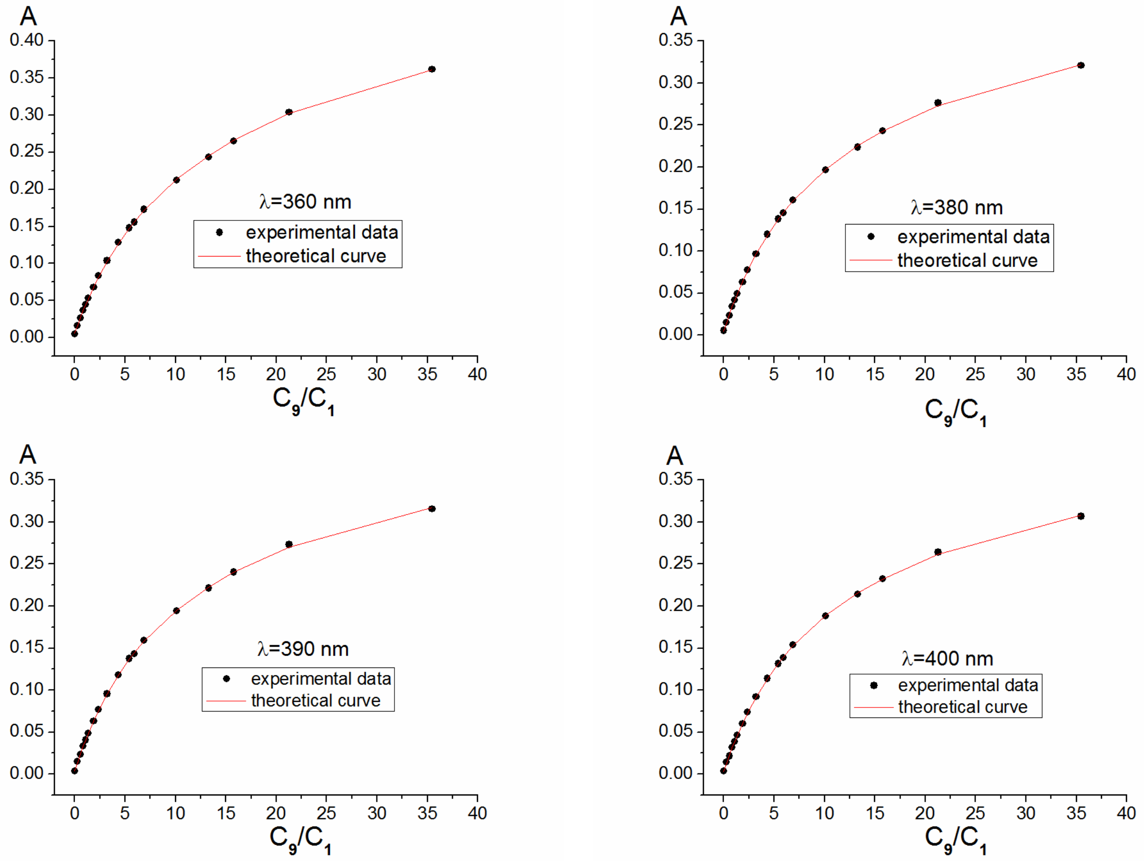

Figure S48: The binding curves at different wavelengths for the molecular clip **1** with guest **9** (C_{clip} = 8.22 × 10^{-4} M)

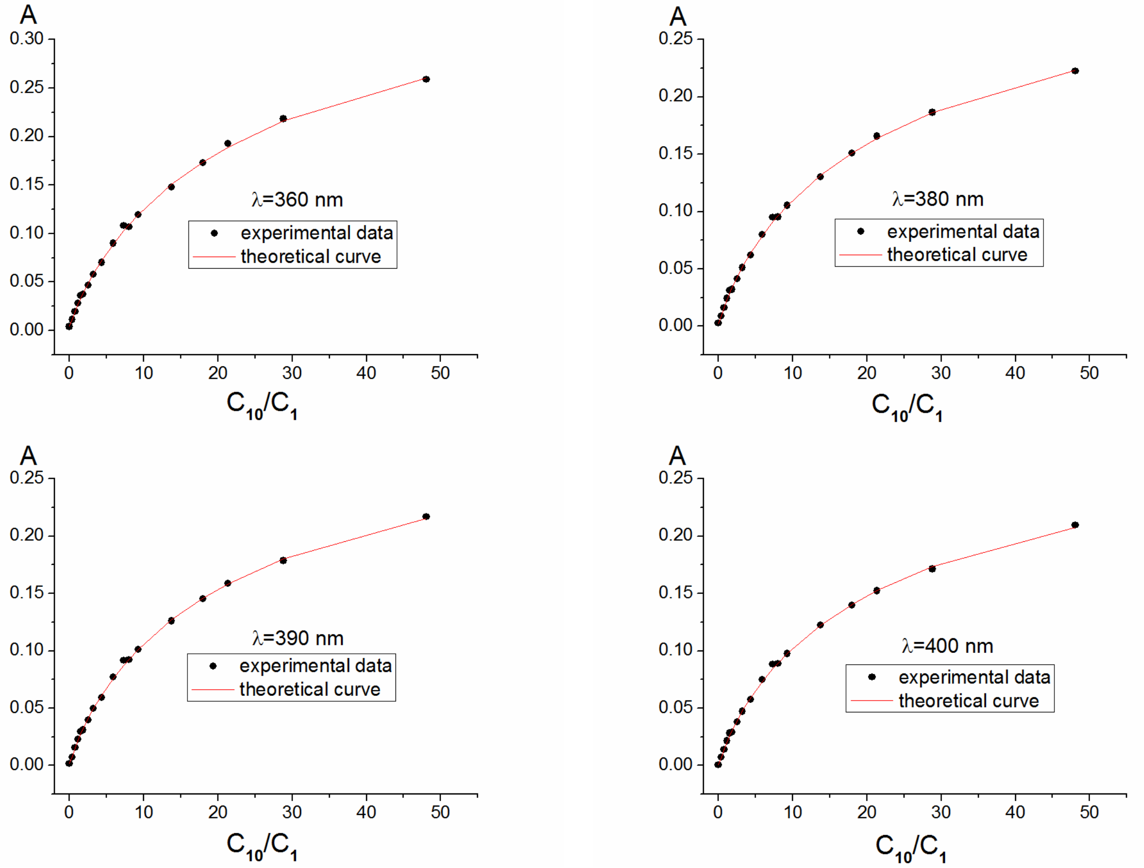

Figure S49: The binding curves at different wavelengths for the molecular clip **1** with guest **10** (C_{clip} = 9.41 × 10^{-4} M)

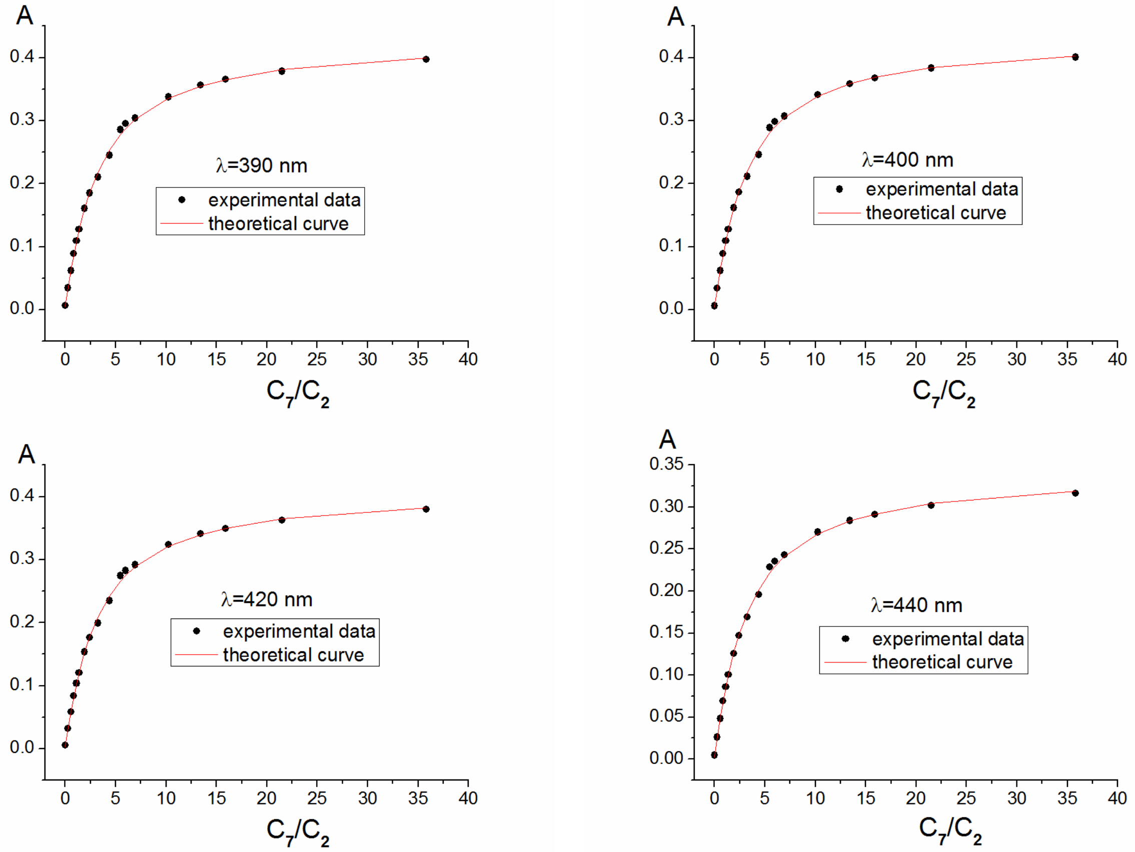

Figure S50: The binding curves at different wavelengths for the molecular clip **2** with guest **7** (C_{clip} = 1.03 × 10^{-3} M)

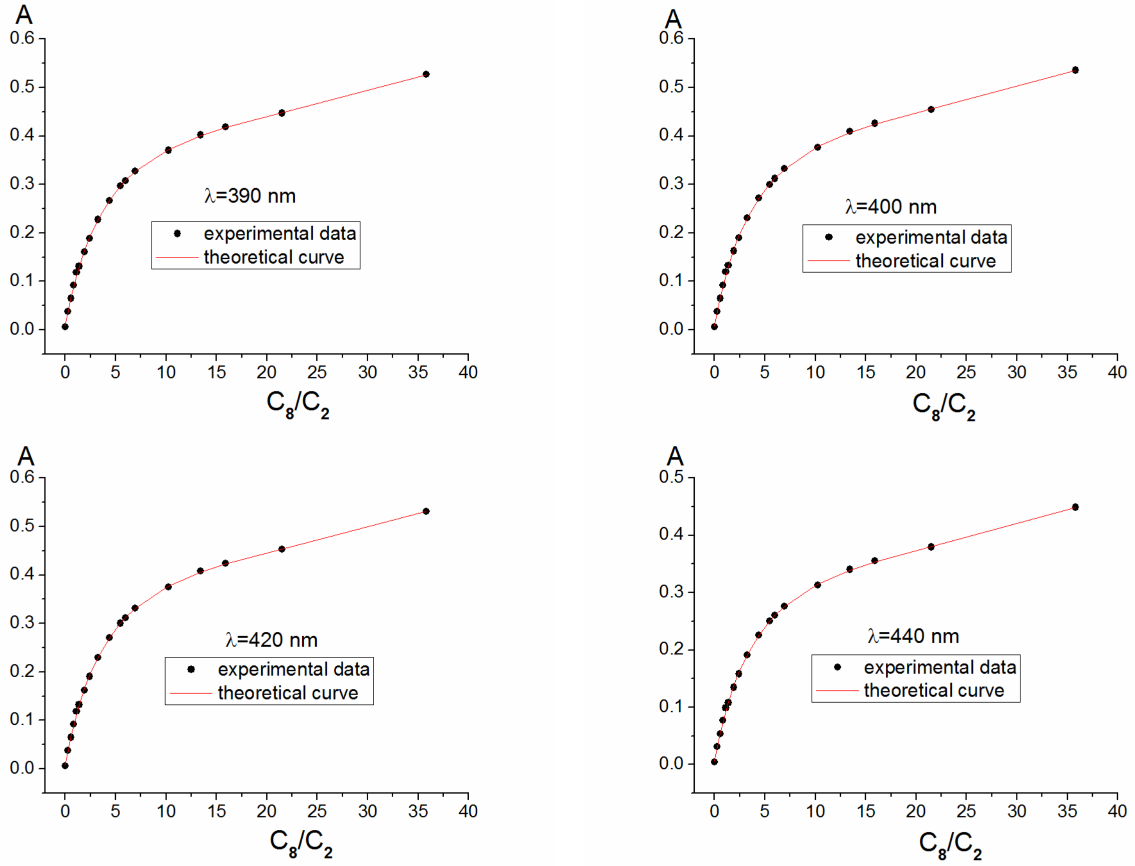

Figure S51: The binding curves at different wavelengths for the molecular clip **2** with guest **8** (C_{clip} = 1.1 × 10^{-3} M)

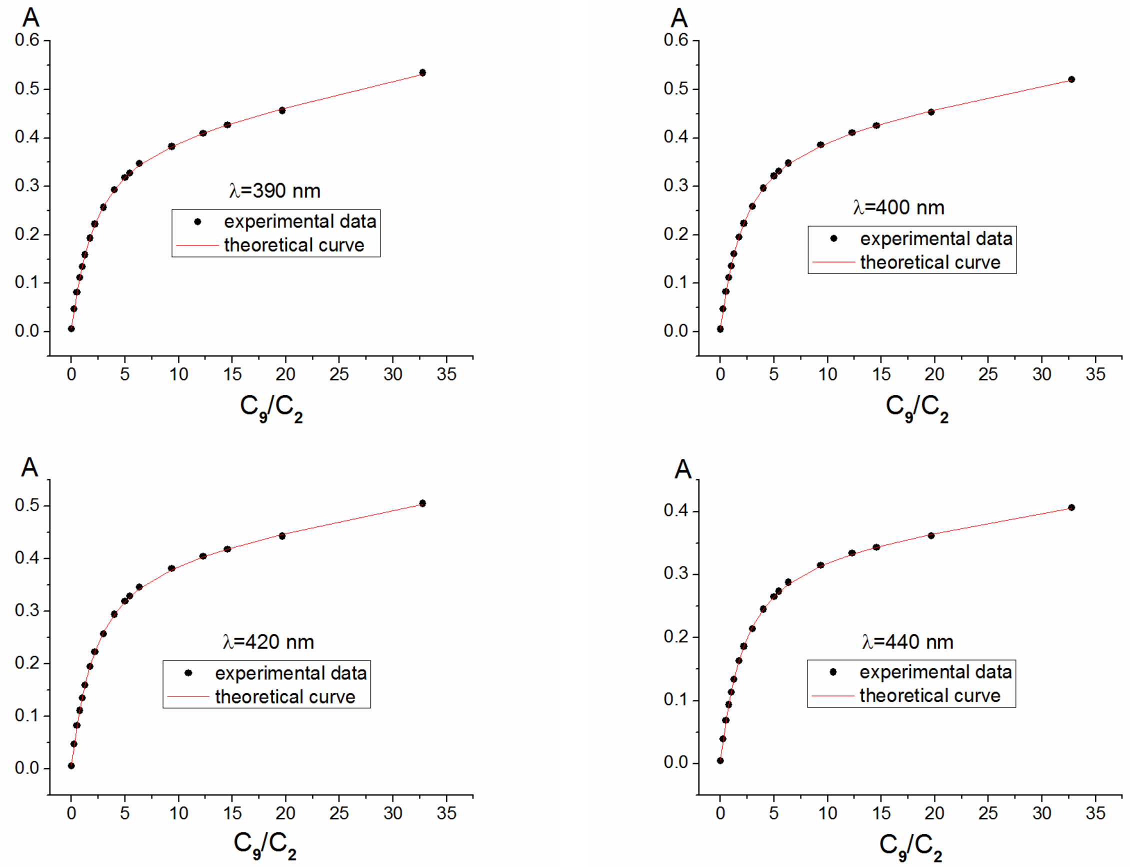

Figure S52: The binding curves at different wavelengths for the molecular clip **2** with guest **9** (C_{clip} = 1.02 × 10^{-3} M)

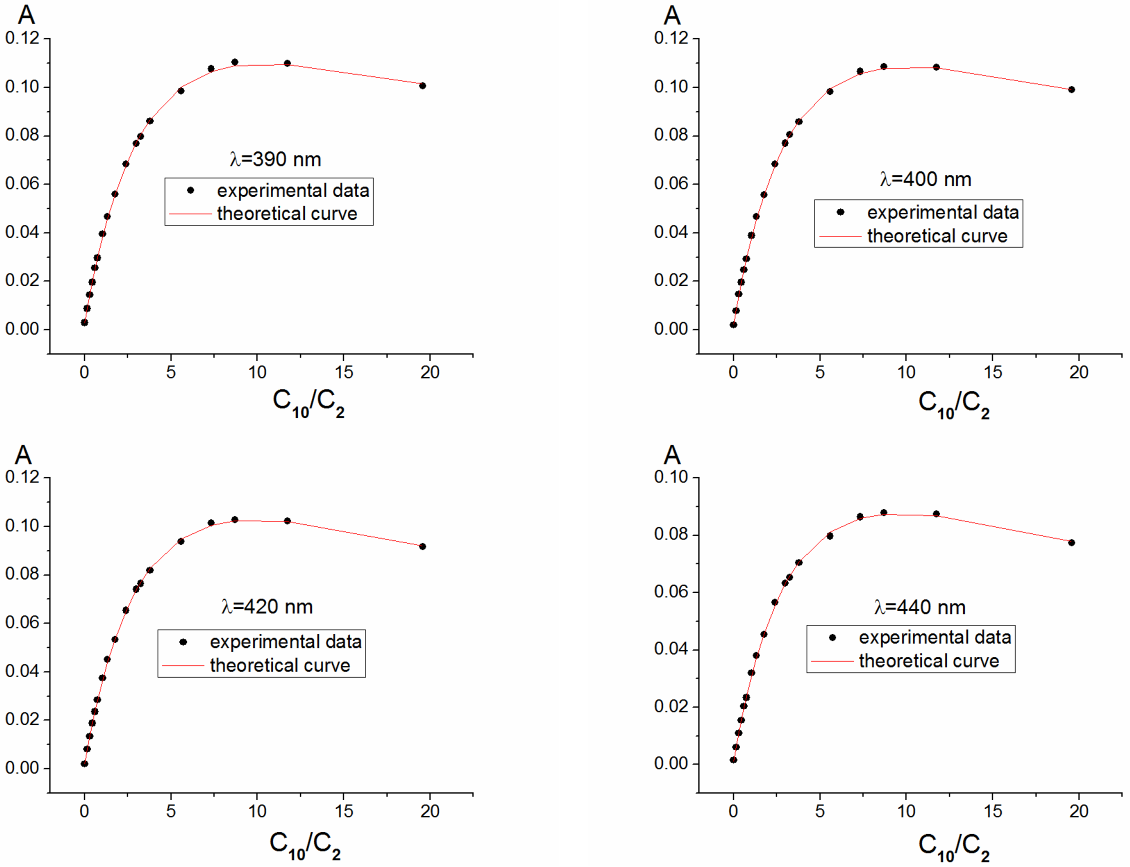

Figure S53: The binding curves at different wavelengths for the molecular clip **2** with guest **10** (C_{clip} = 1.11 × 10^{-3} M)

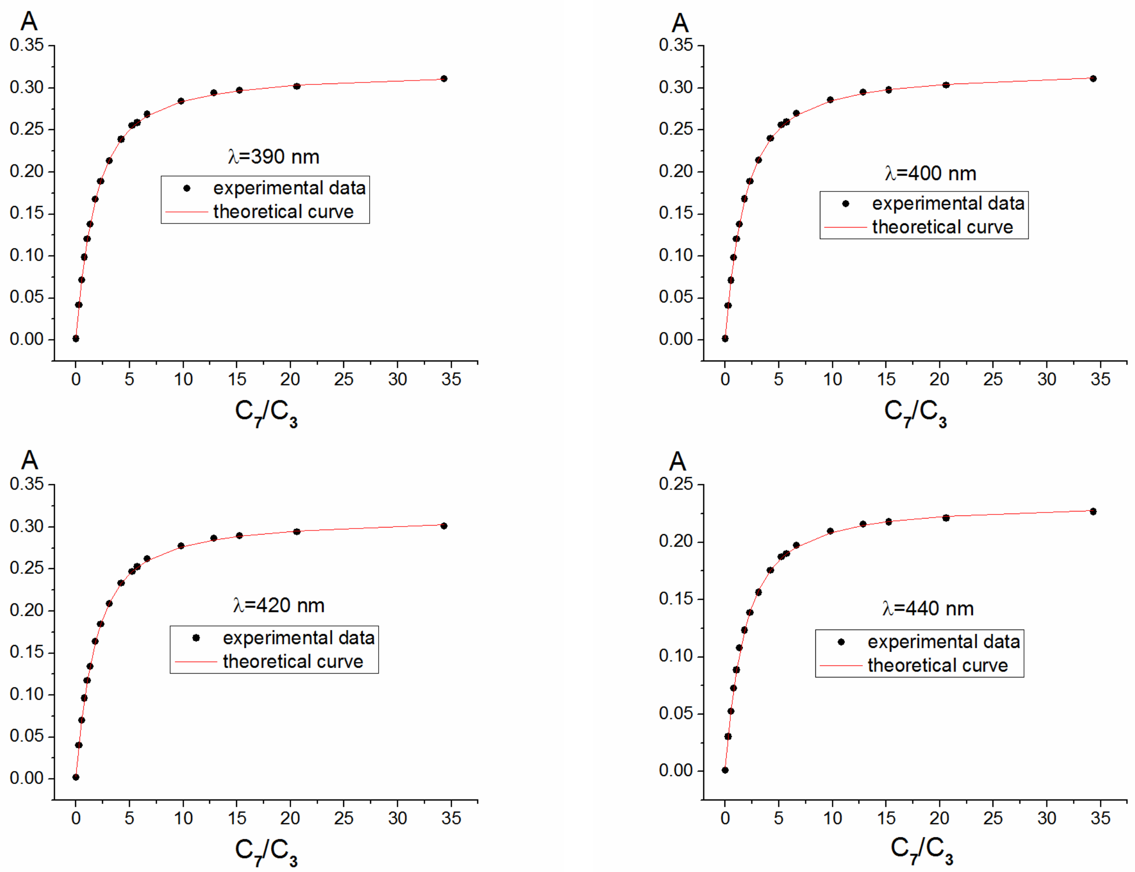

Figure S54: The binding curves at different wavelengths for the molecular clip **3** with guest **7** (C_{clip} = 8.05 × 10^{-4} M)

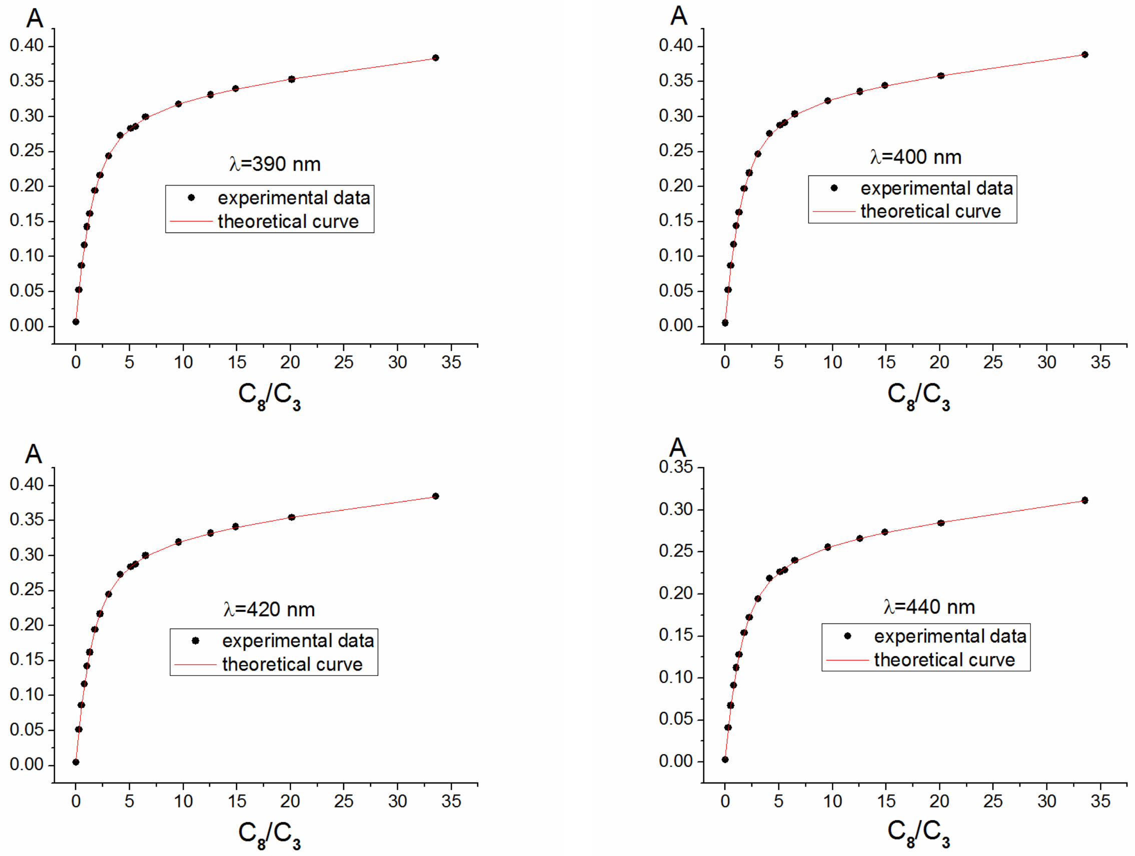

Figure S55: The binding curves at different wavelengths for the molecular clip **3** with guest **8** (C_{clip} = 7.8 × 10^{-4} M)

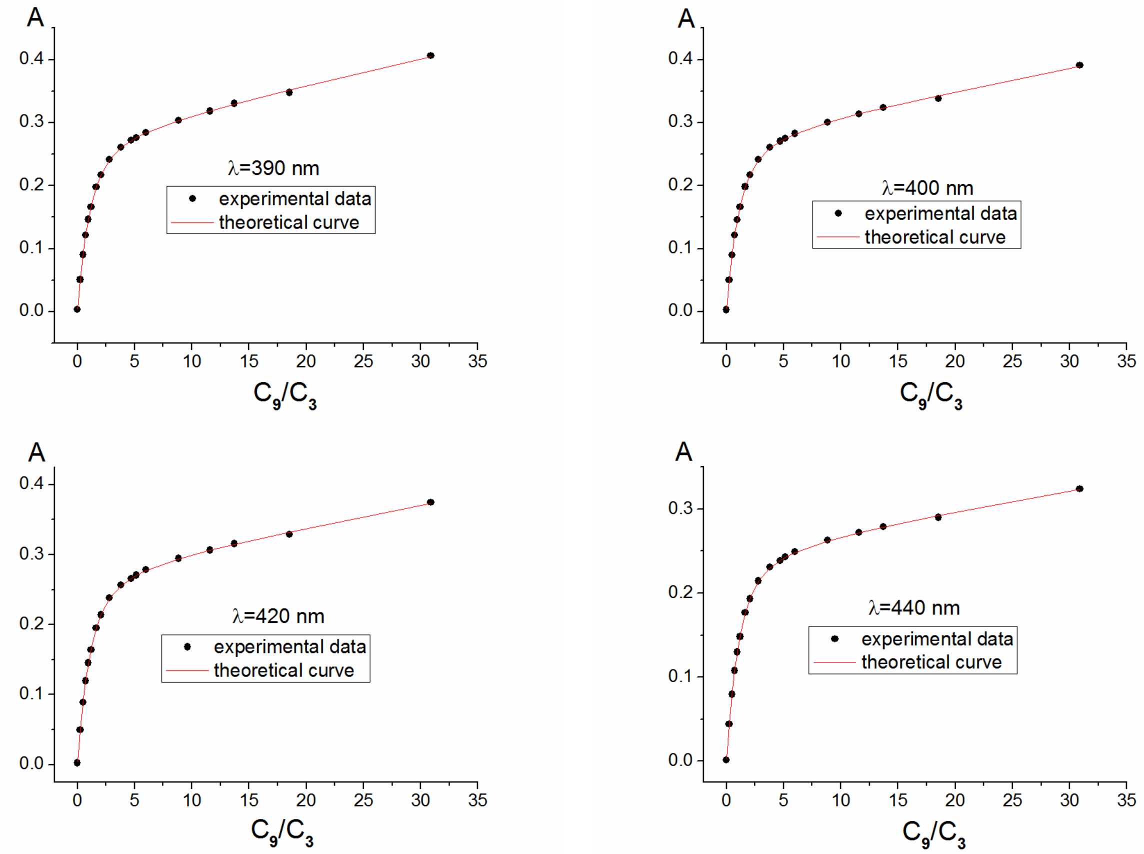

Figure S56: The binding curves at different wavelengths for the molecular clip **3** with guest **9** (C_{clip} = 7.76 × 10^{-4} M)

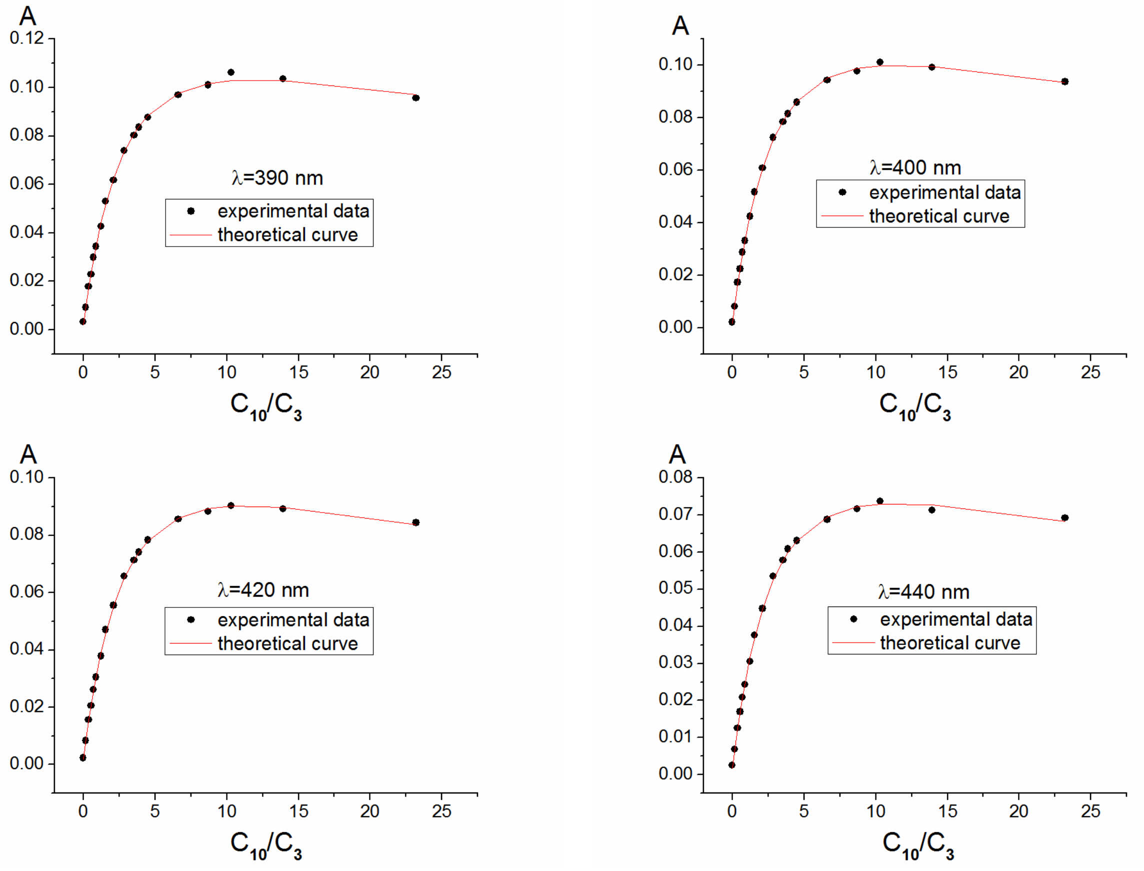

Figure S57: The binding curves at different wavelengths for the molecular clip **3** with guest **10** (C_{clip} = 8.28 × 10^{-4} M)

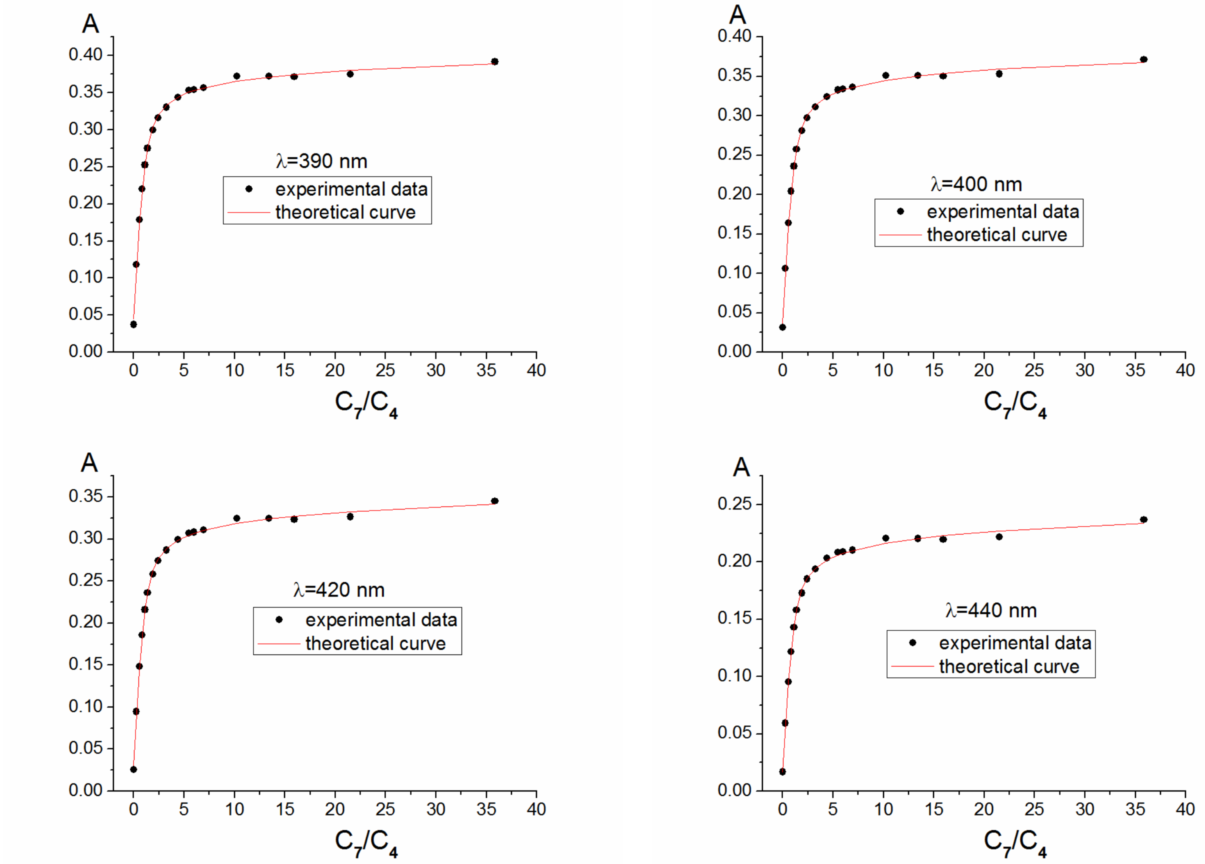

Figure S58: The binding curves at different wavelengths for the molecular clip **4** with guest **7** (C_{clip} = 8.17 × 10^{-4} M)

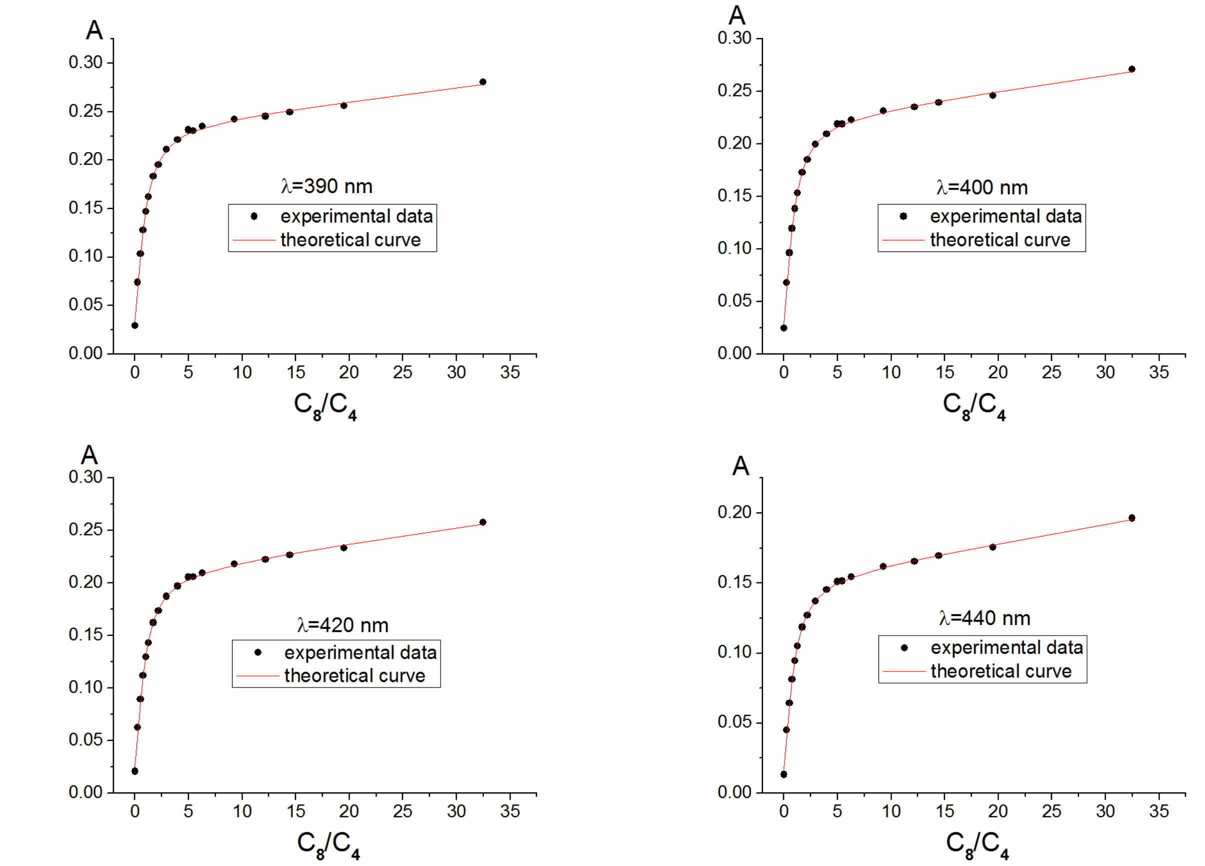

Figure S59: The binding curves at different wavelengths for the molecular clip **4** with guest **8** (C_{clip} = 5.34 × 10^{-4} M)

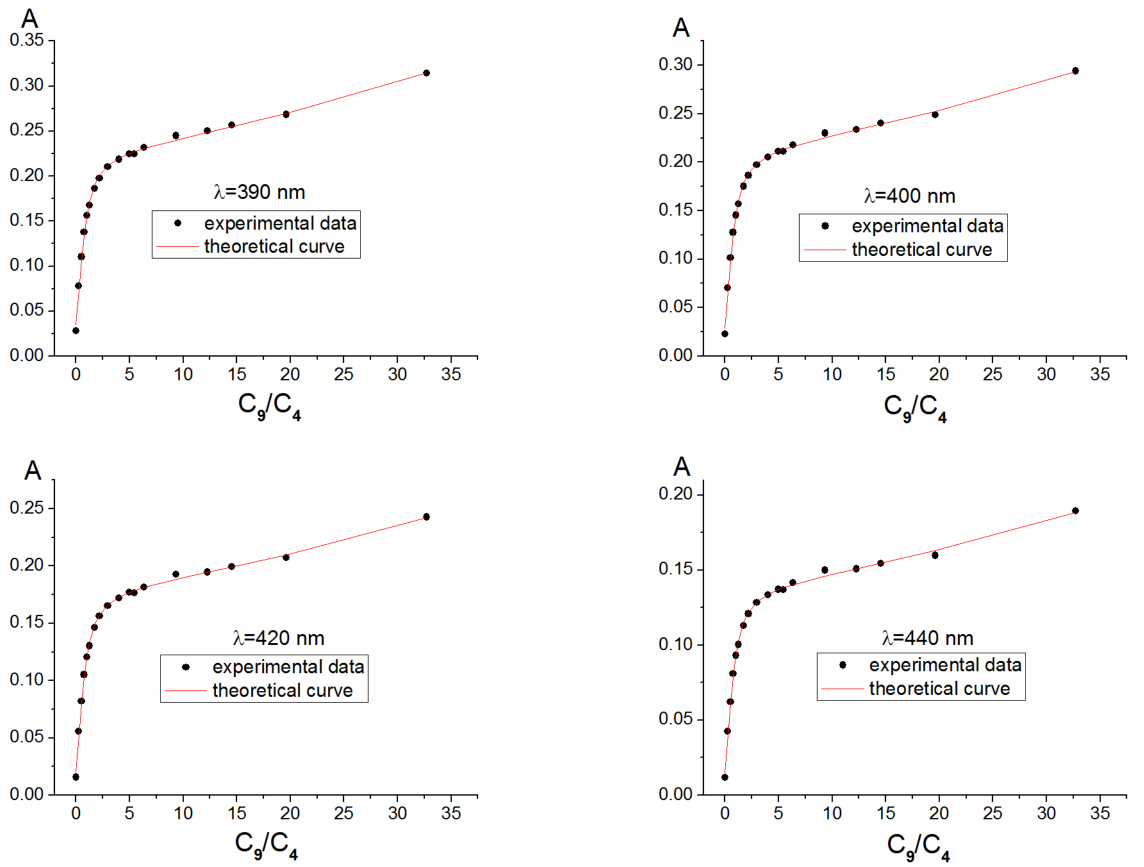

Figure S60: The binding curves at different wavelengths for the molecular clip **4** with guest **9** (C_{clip} = 5.34 × 10^{-4} M)

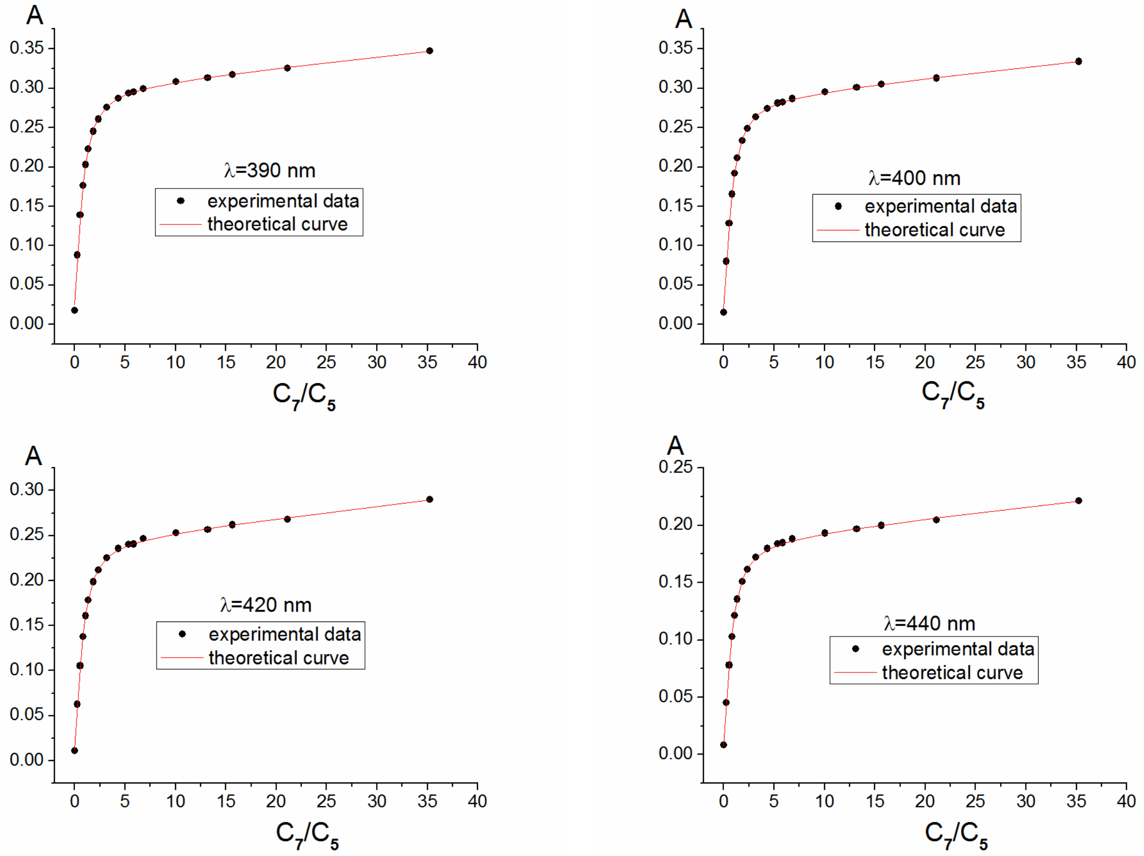

Figure S61: The binding curves at different wavelengths for the molecular clip **5** with guest **7** (C_{clip} = 7.13 × 10^{-4} M)

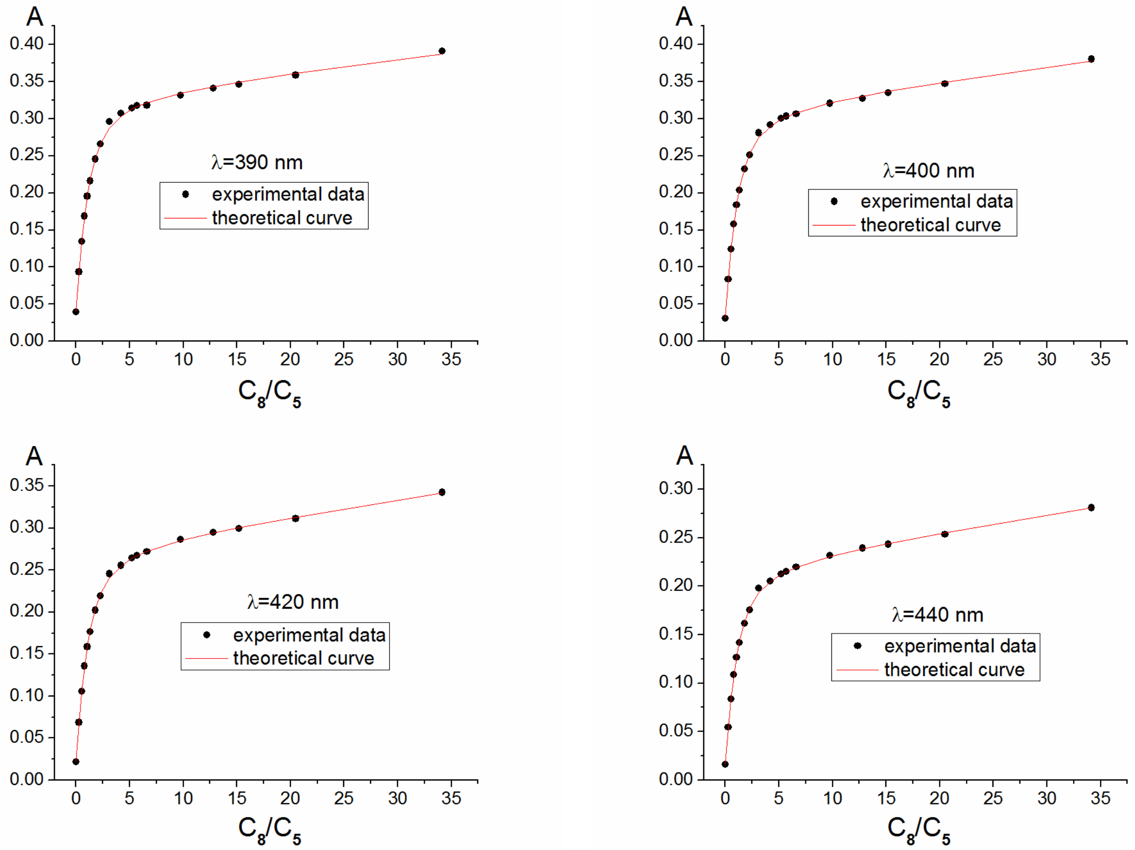

Figure S62: The binding curves at different wavelengths for the molecular clip **5** with guest **8** (C_{clip} = 6.71 × 10^{-4} M)

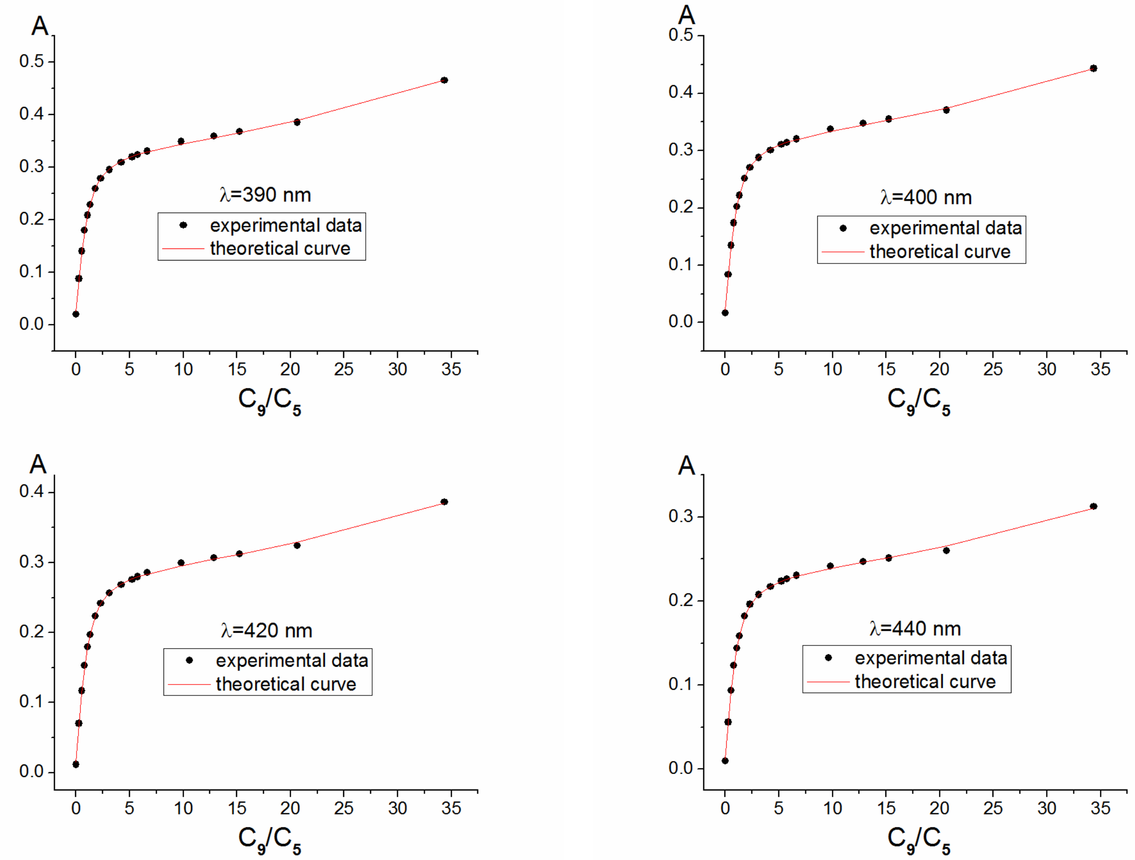

Figure S63: The binding curves at different wavelengths for the molecular clip **5** with guest **9** (C_{clip} = 7.14 × 10^{-4} M)

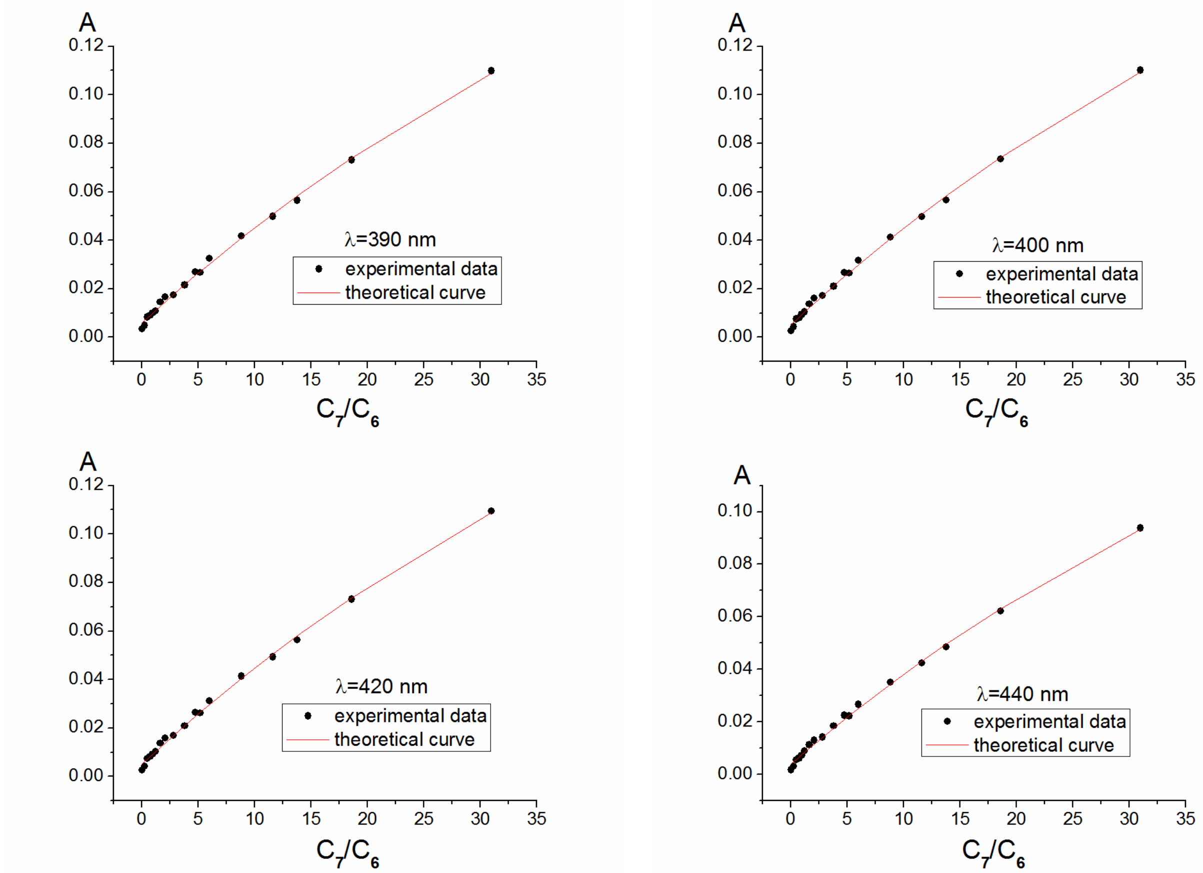

Figure S64: The binding curves at different wavelengths for the molecular clip **6** with guest **7** (C_{clip} = 8.97 × 10^{-4} M)

Table S1: The crystallographic data and experimental parameters for complexes **2@7**, **2@8**, **2@9**, **3@7**, **3@8**, **3@9**, **5@7**, **2**@1**0** and **3@10**.

Parameter	**2@7**	**2@8**	**2@9**	**3@7**	**3@8**	**3@9**	**5@7**	**2@10**	**3@10**
Unit cell<br>a, Å	17.6255(4)	13.5352(3)	15.0134(6)	12.1698(4)	13.1738(4)	14.0779(3)	12.2582(7)	15.6903(4)	17.9713(3)
b, Å	21.8818(5)	19.0864(5)	15.3160(8)	16.2787(6)	15.1475(4)	15.5960(5)	16.7161(9)	17.5825(2)	19.0283(3)
c, Å	18.8953(4)	25.6142(5)	16.8450(8)	17.3973(8)	19.1315(5)	18.7279(5)	18.555(1)	30.3495(7)	27.0060(5)
α, deg	90.0	90.0	73.484(4)	97.280(3)	107.536(2)	111.179(3)	80.366(4)	90.654(1)	90.0
β, deg	90.0	101.882(2)	87.430(4)	94.265(3)	104.120(3)	90.890(2)	84.428(5)	103.001(2)	100.432(1)
γ, deg	90.0	90.0	80.344(4)	96.959(3)	92.529(2)	100.848(2)	82.931(5)	104.924(2)	90.0
V, $Å^3$	7298.6(3)	6475.4(2)	3661.0(3)	3379.5(2)	3501.6(2)	3749.9(2)	3708.5(4)	7861.4(3)	9082.4(3)
F(000)	3256	3072	1656	1553	1612	1708		3504	3688
Crystal system	Orthorhombic	Monoclinic	Triclinic	Triclinic	Triclinic	Triclinic	Triclinic	Triclinic	Monoclinic
Space group	Pbcn	$P2_1/c$	$P\bar{1}$	$P\bar{1}$	$P\bar{1}$	$P\bar{1}$	$P\bar{1}$	$P\bar{1}$	$P2_1$
Z	4	14	2	2	2	2	2	4	4
T, K	100	100	100	100	100	100	100	100	100
μ, mm^{-1}	0.159	0.176	0.162	0.168	0.167	0.162	0.163	0.157	0.142
D_{calc}, g/cm^3	1.420	1.512	1.438	1.463	1.465	1.446	1.450	1.414	1.285
$2\Theta_{max}$, grad	52.0	60.0	60.0	56.0	66.0	60.0	50.0	50.0	50
Measured reflections	31002	20823	29502	16409	24503	24523	23066	66784	59861
Independent reflections	8349	12961	17889	10757	17116	15054	12381	27336	29349
R_{int}	0.023	0.038	0.051	0.027	0.021	0.041	0.048	0.022	0.034
Reflections with F>4σ(F)	5604	9947	6353	7850	13434	11455	5718	19909	22605
Parameters	586	932	976	988	979	1029	1000	2090	2173
R_1	0.056	0.057	0.067	0.070	0.064	0.055	0.056	0.089	0.096
wR_2	0.159	0.155	0.147	0.184	0.195	0.136	0.134	0.256	0.260
S	1.039	1.029	1.015	1.023	1.074	1.041	0.838	1.089	1.099
CCDC number	1555840	1555846	1555845	1555839	1555843	1555841	1555842	1555847	1555844

Table S2: Geometrical characteristics of the intermolecular interactions between molecules **2**, **3**, **5** and **7**–**10** in host–guest complexes.

Contact	H…A, Å	D-H…A, deg
Complex **2@7**		
C24-H…C29(π)	2.86	159
C24-H…C29(π)	2.86	159
C26-H…O5a–C	2.36	134
C26-H…O5a–C	2.36	134
C27-H…O4a–C	2.39	125
C27-H…O4a–C	2.39	125
C28-H…O1=C	2.30	130
C28-H…O1=C	2.30	130
stacking	3.55	
stacking	3.55	

Complex **2@8**		
C17-H…C58(π)	2.75	153
C49-H…O4–C	2.43	130
C50-H…O3–C	2.37	138
C52-H…C34 (π)	2.82	134
C52-H…C37 (π)	2.84	108
C53-H…O1=C	2.32	150
C57-H…O4–C	2.44	130
C59-H…O7–C	2.21	133

Complex **2@9**		
O13-H…O1=C	2.29	170
O14-H…O9–C	1.96	169
C24-H…C60 (π)	2.87	154
C42-H…O011 –C	2.47	132
C49-H…O3–C	2.44	143
C52-H…C34 (π)	2.81	157
C52-H…C35 (π)	2.89	132
C52-H…C37 (π)	2.84	142
C53-H…O1=C	2.29	154
C59-H…O10–C	2.45	142
C61-H…O7=C	2.18	147

Complex **2@10** Molecule A		
C50a-H50a…O2a=C	2.28	146
C60a-H60a…O1a=C	2.32	150
C61a-H61…C23a (π)	2.91	131
C63a-H63a…O6a	2.44	142
C64a-H64a…O5a	2.44	139
stacking	3.62	
Complex **2@10** Molecule B		

C60b-H60b…O1b=C	2.46	136
C61b-H61b…C9b	2.83	116
C50b-H50b…O2b=C	2.41	128
C36b-H36c…C51b	2.73	158
C36b-H36c…C52b	2.79	149
C64b-H64b…O10b	2.32	135
C64b-H64b…O9b	2.45	130
C63b-H63b…O11b	2.29	147

Complex **3@7**		
C17-H…C56 (π)	2.85	157
C53-H…O11–C	2.34	157
C56-H…C13 (π)	2.88	117
C58-H…O10a–C	2.31	146
C59-H…O5–C	2.30	174
C60-H…O3–C	2.46	130
C62-H…C42 (π)	2.77	115
C63-H…O8=C	2.20	138
C64-H…O6–C	2.33	149
stacking	3.52	
stacking	3.51	

Complex **3@8**		
O16a-H…O10–C	2.14	170
C17-H…C55 (π)	2.82	147
C52-H…C57 (π)	2.90	170
C56-H…O1=C	2.24	149
C60-H…O5–C	2.24	135
C64-H…O8=C	2.39	155
stacking	3.52	

Complex **3@9**		
O16-H…O1=C	2.06	161
C26-H…C63 (π)	2.72	161
C52-H…O15–C	2.52	152
C53-H…O1=C	2.30	157
C56-H…O11–C	2.44	163
C57-H…O12–C	2.37	151
C63-H…O8=C	2.33	132
C65-H…O4–C	2.49	139
stacking	3.34	

Complex **3@10**<br>Molecule A		
C56a-H56a…O1a	2.40	135
C57a-H57a…C29a	2.80	119
C62a-H62a…C26a	2.76	133
H33a-H33a…C62a	2.78	150
C59a-H59a…O6a	2.38	150

stacking	3.45	
Complex **3@10**<br>Molecule B		
C60b-H60b…O1b	2.45	132
C59b-H59b…C25b	2.75	123
C54b-H54b…C29b	2.78	124
C57b-H57b…O5b	2.58	149
C56b-H56b…O6b	2.38	157
Stacking	3.55	
stacking	3.75	

Complex **5@7**		
C1a-H…O8	2.36	137
C3a-H…O2	2.37	131
C4a-H…C42	2.89	118
C8a-H…O1	2.34	136
C10a-H…O13	2.54	131
C11a-H…O17	2.42	150
C12a-H…O4	2.50	145
C12a-H…O6	2.15	156
stacking	3.59	
stacking	3.64	
